# Protein–lipid charge interactions control the folding of outer membrane proteins into asymmetric membranes

**DOI:** 10.1038/s41557-023-01319-6

**Published:** 2023-09-14

**Authors:** Jonathan M. Machin, Antreas C. Kalli, Neil A. Ranson, Sheena E. Radford

**Affiliations:** 1 Astbury Centre for Structural Molecular Biology, School of Molecular and Cellular Biology, Faculty of Biological Sciences, University of Leeds, Leeds, UK; 2 Leeds Institute of Cardiovascular and Metabolic Medicine, School of Medicine, University of Leeds, Leeds, UK

**Keywords:** Molecular biophysics, Protein folding

## Abstract

Biological membranes consist of two leaflets of phospholipid molecules that form a bilayer, each leaflet comprising a distinct lipid composition. This asymmetry is created and maintained in vivo by dedicated biochemical pathways, but difficulties in creating stable asymmetric membranes in vitro have restricted our understanding of how bilayer asymmetry modulates the folding, stability and function of membrane proteins. In this study, we used cyclodextrin-mediated lipid exchange to generate liposomes with asymmetric bilayers and characterize the stability and folding kinetics of two bacterial outer membrane proteins (OMPs), OmpA and BamA. We found that excess negative charge in the outer leaflet of a liposome impedes their insertion and folding, while excess negative charge in the inner leaflet accelerates their folding relative to symmetric liposomes with the same membrane composition. Using molecular dynamics, mutational analysis and bioinformatics, we identified a positively charged patch critical for folding and stability. These results rationalize the well-known ‘positive-outside’ rule of OMPs and suggest insights into the mechanisms that drive OMP folding and assembly in vitro and in vivo.

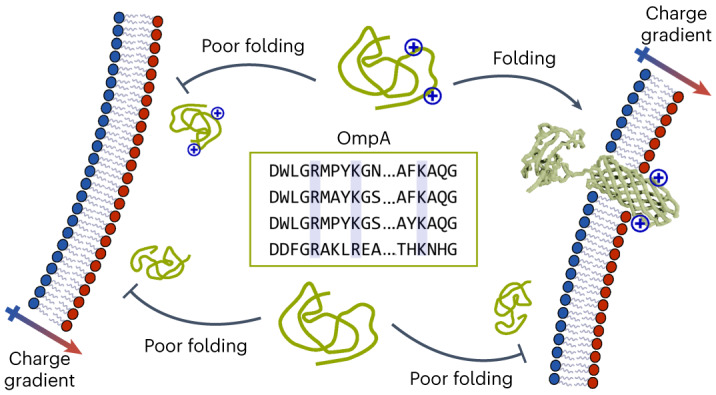

## Abstract (web summary)

Biological membranes are asymmetric bilayers, but little is known about how this asymmetry modulates membrane protein folding or stability. Now, folding and stability assays with bacterial outer membrane proteins reveal an exquisite sensitivity to asymmetric membrane charge distribution and a required matching of protein charge for efficient folding.

## Main

Membrane proteins carry out many essential functions in biology and are therefore major drug targets^1,2^. Recent progress has been made in understanding how membrane proteins fold^3^, but while lipid–protein interactions are known to be important, their precise roles remain unclear, with most information gleaned empirically on a case-by-case basis^4,5^. Most biological membranes have asymmetry in lipid composition between the leaflets of their bilayer^6,7^. This asymmetry is dependent on membrane type and cell status^8^, and the plethora of enzymes dedicated to creating and maintaining bilayer asymmetry^9,10^, as well as disease states featuring mis-regulated asymmetry^11^, demonstrate its importance. However, generating stable, lipid-asymmetric systems of the quality and quantity needed for in vitro folding studies is challenging, so little is currently known about the interplay of membrane asymmetry and protein folding.

A number of methods to generate asymmetric phospholipid bilayers have been developed, including supported bilayers^12,13^, phase-transfer approaches^14,15^ and liposome hemifusion^16^. Asymmetry can also be generated by cyclodextrin (CD)-mediated lipid exchange^17,18^, which has been used to generate liposomes with asymmetric bilayers^19,20^. These methods have been used to study membrane protein folding, with the rate of folding of perfringolysin O (ref. ^21^) and the ‘pH low insertion peptide’^22^ each being modulated by charge asymmetries across the bilayer. However, both of these proteins exist in stable, water-soluble forms that only insert into membranes under specific conditions^23,24^. It is thus difficult to generalize these finding to integral membrane proteins, which require a membrane to adopt their native fold.

Outer membrane proteins (OMPs) from Gram-negative bacteria^25,26^ have a transmembrane β-barrel fold, in which membrane-spanning β-strands are linked by longer extracellular loops and shorter intracellular turns^27^. In vitro folding studies of OMPs of different sizes have shown that the membrane helps to regulate folding^28^. For example, folding is faster when bilayers contain lipids with short acyl chains^29^, less saturated lipids^30^ or more membrane defects^31^. Lipid head groups also modulate folding, with phosphoethanolamine (PE) and phosphoglycerol (PG) introducing a kinetic barrier for folding into C_10:0_ lipid bilayers^32,33^. However, recent work with C_14:0_ lipids did not show this effect, perhaps because the additional kinetic barrier of a thicker membrane dominates folding^34^. The primary structure of an OMP is also critical, perhaps even more than the properties of the membrane^34^, a concept supported by mutational analysis of folding efficiency for OmpA, EspP and OmpC variants in vivo^35^. While OMP folding into membranes of different lipid composition has been studied for decades^25,36^, the role of membrane asymmetry has not been studied in detail to date.

**Extended Data Fig. 1:**
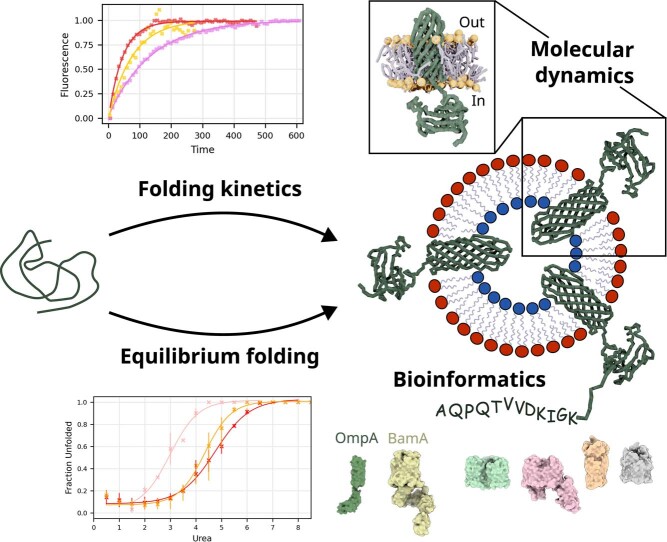
Combining experiments, simulations and bioinformatics to reveal how charge patterning in OMP loops and membrane asymmetry synergise for productive folding and stability. Following generation of charge asymmetric liposomes (depicted here by red and blue headgroups), OMP folding kinetics (top left, measured by Trp fluorescence) and stability (bottom left, measured by cold SDS PAGE) of two model OMPs, OmpA and BamA (green and yellow space fill structures) were measured and compared with the results for the same lipids in symmetric membranes (not shown). Molecular dynamics of OMPs pre-folded into different lipid systems (top right), as well as structural and sequence bioinformatics (bottom right) for 300 and 19000 OMPs, respectively (six are depicted) were then used to identify residues involved in the modulation of folding rates and stabilities upon interaction with the lipid head group.

In this study, we used CD-mediated lipid exchange to generate charge-asymmetric liposomes using dimyristoyl-phosphatidylcholine (DMPC) and dimyristoyl-phosphatidylglycerol (DMPG) lipids, as well as dimyristoyl-phosphatidylethanolamine (DMPE) and dimyristoyl-phosphatidylserine (DMPS) lipids, and validated their asymmetry using measurements and predictions of their ζ-potential. We found for two model OMPs, 8-stranded OmpA and 16-stranded BamA, that folding rate and stability are modulated by a leaflet-specific distribution of negatively charged lipid head groups, irrespective of acyl chain length. Using molecular dynamics (MD), we identified specific, positively charged residues in the extracellular loops of OmpA that interact with lipids and found that they are critical for OmpA folding in vitro. Bioinformatic analysis of >300 structures and >19,000 sequences of OMPs revealed a highly conserved enrichment of positively charged residues in the extracellular loops close to the membrane surface. Collectively using this integrative approach of experiment, bioinformatics and simulation (Extended Data Fig. 1), our results reveal that efficient OMP folding requires a previously uncharted synergy between the lipid charge in each leaflet of the bilayer and a signature region (the ‘patch of external positive residues’) of Lys/Arg in the extracellular loops of the folding OMP. This finding is particularly important given the high charge asymmetry in the lipopolysaccharide-containing outer membrane (OM). The results provide new insights into how lipid organization modulates OMP folding and stability in vitro, have implications for understanding OMP folding in vivo, and suggest new strategies to control OMP folding and stability for biotechnological applications.

## Results

### Generating asymmetric liposomes

Charge distribution in membrane proteins is used to control protein topology and stability in vivo^37^, for example, the ‘positive-inside’ rule, which modulates the orientation of proteins in the plasma membrane/bacterial inner membrane^38,39^. By contrast, the ‘positive-outside’ rule for OMPs, with more Lys/Arg residues in the extracellular loops than in the intracellular turns, is postulated to stabilize OMPs via their interaction with lipopolysaccharide (LPS) in the asymmetric bacterial OM^40,41^. However, reducing positive charge by shortening the extracellular loops of OMPs, either individually or in combination, does not alter the folding topology^42,43^, suggesting that charge has a different, currently unknown, role in OMP assembly.

**Fig. 1:**
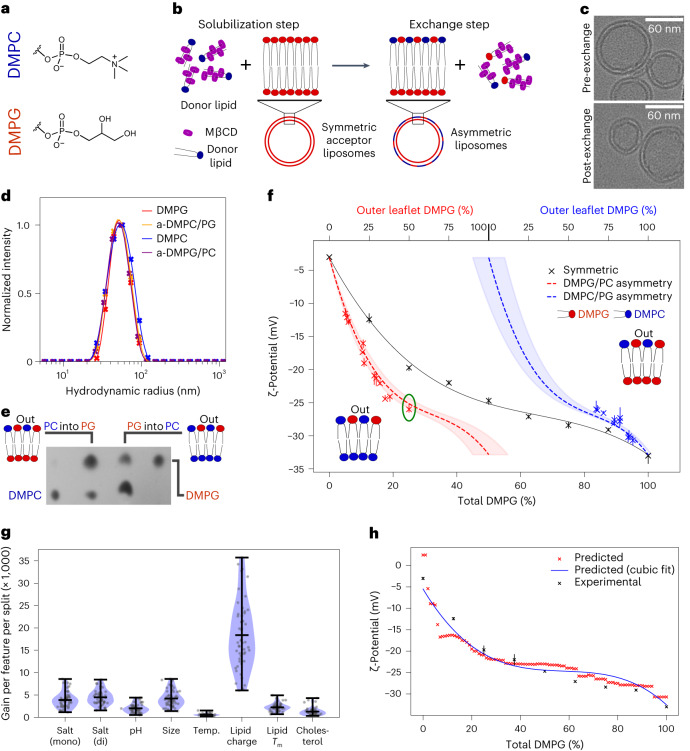
Generating and validating asymmetric LUVs. **a**, Head group structures of the DMPC and DMPG lipids. The same colour code is used throughout. **b**, Overview of asymmetric liposome generation by MβCD-mediated exchange. **c**, Pre- and post-exchange liposomes imaged by cryoEM. The liposomes are smaller than observed using DLS as small liposomes preferentially move into the ice. **d**, Pre- and post-exchange liposome size by DLS. **e**, Sample TLC plate showing the introduction of the DMPC lipid into DMPG liposomes by CD-mediated exchange, and vice versa. Outer two lanes, DMPC (left) and DMPG (right) liposomes before exchange; inner two lanes, exchanging DMPC into DMPG liposomes (left) and DMPG into DMPC liposomes (right). **f**, ζ-Potential by lipid content for symmetric (black line) and asymmetric liposomes DMPC/PG and DMPG/PC. The theoretical asymmetry lines are shown with an error margin of 10% (shaded region). The generated asymmetric liposome samples (DMPC/PG, blue; DMPG/PC, red) show range bars from repeat ζ-potential measurements (the centre is the mean average, *n* ≥ 3). The green-circled measurement is discussed in the text. **g**, Feature importance (gain per feature per split) in the liposome ζ-potential model. The bars represent the data minima, median and maxima (*n* = 50). **h**, Agreement between predicted and experimental ζ-potential values (errors are shown as range bars, with *n* ≥ 3) for DMPC/PG LUVs in buffer solution (100 mM NaCl, 20 mM Tris-Cl, pH 8.5). Source data

**Extended Data Fig. 2:**
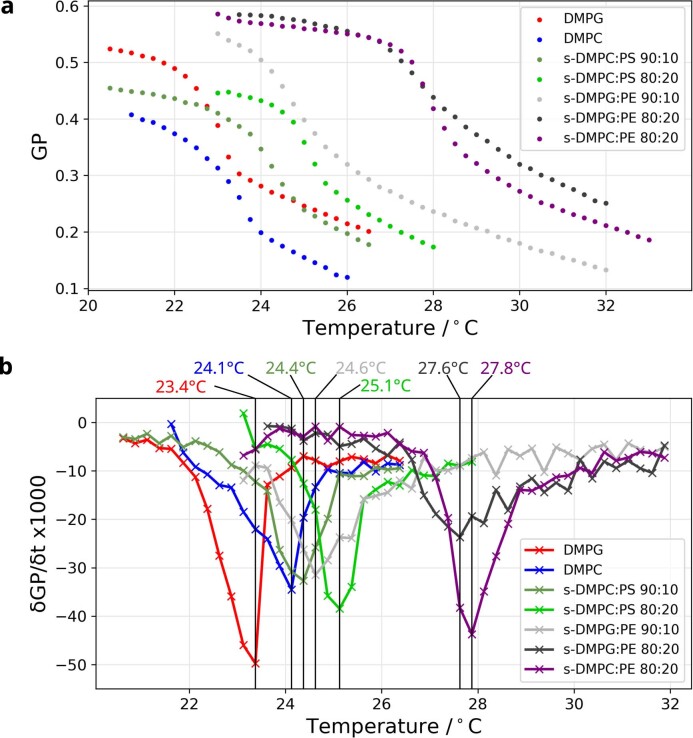
Global lipid phase transition behaviour for liposomes used in this study, measured using laurdan fluorescence. **(a)** The GP (generalised polarisation) ratio of fluorescence at 440 and 490 nm (see Methods) against temperature for pure DMPC and pure DMPG liposomes, and symmetric DMPS-DMPC, DMPE-DMPG and DMPE-DMPC lipid mixes, as indicated, measured using 0.25 °C intervals. **(b)** The first derivative of the GP, with the implied T_m_s. Source data

To determine the effect(s) of lipid charge asymmetry on OMP folding, membrane systems based on DMPC and DMPG were created. These lipids have the same C_14:0_ acyl chains, generating a bilayer with a similar hydrophobic thickness to the native bacterial OM. They also have similar head group sizes and lipid phase transition temperatures (*T*_m_) of ~24 and 23 °C, respectively (Extended Data Fig. 2). Importantly, DMPC is a neutral zwitterion, while DMPG is negatively charged (Fig. 1a). The folding of OMPs into symmetric membranes of dimyristoyl (DM) lipids has been widely used to study OMP folding (for example, see refs. ^29,34^), providing the ideal framework within which to begin to determine the role of bilayer asymmetry in OMP folding and stability. Asymmetric liposomes containing DMPC and DMPG were generated by methyl β-cyclodextrin (MβCD)-mediated exchange (Fig. 1b). Symmetric and asymmetric lipid membranes are henceforth indicated by the prefixes ‘s’ and ‘a’, respectively, and asymmetric liposomes are denoted as donor lipid/acceptor liposome, while symmetric lipid ratios are separated by a colon (:). Thus, a-DMPG/PC indicates DMPG lipids exchanged into the outer leaflet of DMPC liposomes (all lipid ratios are mol/mol unless otherwise indicated). Following lipid exchange, the integrity and size of the final liposomes were confirmed using cryo-electron microscopy (cryoEM) and dynamic light scattering (DLS; Fig. 1c,d), and removal of MβCD by the detection of residual sugar (Supplementary Fig. 1 and Methods). The ability of MβCD to mediate exchange between the DMPC and DMPG lipids was confirmed using the fluorescent marker dipalmitoyl-phosphatidylethanolamine(DPPE)-rhodamine (Supplementary Fig. 2 and Methods). Thin layer chromatography (TLC), quantified by densitometry, was then used to measure the DMPC-DMPG ratio (within an error of <3%; Supplementary Fig. 3), and hence quantify the extent of lipid exchange (for example, Fig. 1e). Label-free DMPC-DMPG liposome asymmetry was then confirmed by determining the ζ-potential, a measure of particle surface charge, allowing quantification of the amount of neutral DMPC and negatively charged DMPG in the solvent-exposed outer leaflet of liposomes (Supplementary Fig. 4). Alongside the total lipid ratio, the ζ-potential thus provides a direct readout of lipid asymmetry (Fig. 1f). For example, the green-circled a-DMPG/PC exchanged sample has a total DMPG fraction of ~25% (lower *x* axis) and a ζ-potential of −26 mV. These data fall on the dashed, theoretical ‘asymmetry’ line, confirming that this liposome is asymmetric and allowing the outer leaflet DMPG content to be read from the upper *x* axis (50%). Asymmetric liposomes with up to ~30% a-DMPC/PG and ~50% a-DMPG/PC in their outer leaflets could be generated and were stable for at least 72 h in the absence and presence of 8 M urea (Supplementary Fig. 4).

### A predictive model for liposome ζ-potential

To improve our ability to define the asymmetry of different lipid compositions by experimental measurement of the ζ-potential, a machine learning model was constructed to predict the ζ-potential of liposomes (Methods). Using 315 data points (from this study and the literature^19,44–56^), lipid composition was parametrized by (1) the average overall charge per lipid, (2) the average *T*_m_ of all lipids and (3) the fraction of cholesterol present. When combined with five additional liposome/buffer features, this yielded an optimized model with an average mean absolute error (MAE) of ~3.0 mV. Lipid charge dominates the model (Fig. 1g), and parameter ablation indicates that lipid charge, *T*_m_ and salt concentration are the most predictive features (Supplementary Fig. 5). The prediction for DMPG and DMPC lipid mixtures (the training set excluded measured data) is consistent with the experimental data (MAE = 0.86 mV, average experimental measurement range = 0.88 mV; Fig. 1h). DMPC and DMPG lipids are well represented in the training data, but the ζ-potential trends of less well-represented lipids and their mixtures, such as DMPS/DMPC and DMPE/DMPG, were also correctly predicted over the regions experimentally validated, but with a larger error (Supplementary Fig. 6).

### Lipid asymmetry modulates OMP folding rate and stability

**Fig. 2:**
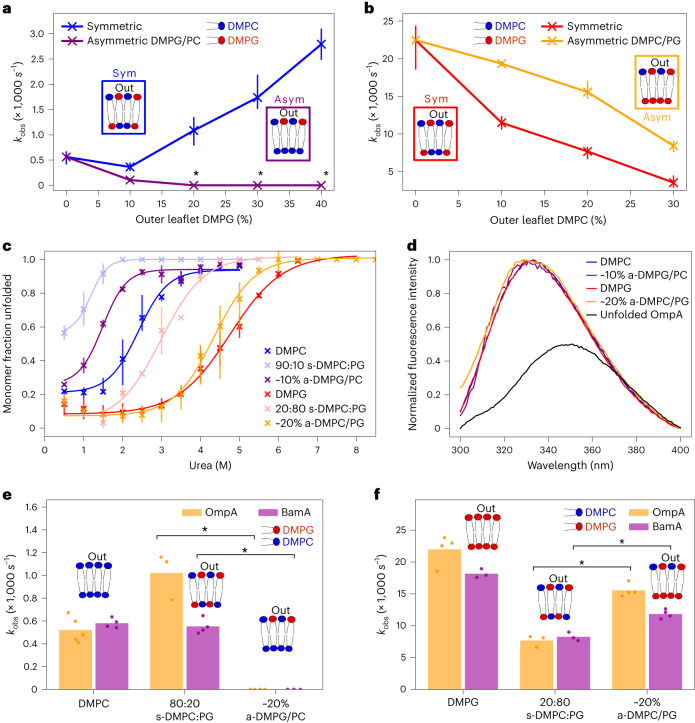
DMPC-DMPG lipid asymmetry significantly affects OMP folding rates. **a**, Folding rate constants (s^−1^) of OmpA into a-DMPG/PC asymmetric liposomes compared with symmetric liposomes with the same outer leaflet composition. The bars represent data ranges (*n* ≥ 3); the asterisks indicate that the folding had not reached completion after 15 h (<75% folded). **b**, Folding rate constants (s^−1^) of OmpA into a-DMPC/PG asymmetric liposomes compared with symmetric liposomes with the same outer leaflet composition. The bars represent data ranges (*n* ≥ 3). **c**, Urea dependence of OmpA folding into DMPC-DMPG symmetric and asymmetric liposomes. The lines are fits to the average of at least two repeats; the bars represent the data range. **d**, Tryptophan fluorescence emission spectra of OmpA folded into LUVs of different composition show that the protein does not unfold after overnight incubation at 30 °C in 8 M urea in any liposome. The spectrum of OmpA unfolded in 7.5 M urea in the absence of lipid is shown for comparison. **e**,**f**, Observed folding rate constant (s^−1^) of OmpA and BamA into DMPC (**e**) and DMPG (**f**) and corresponding asymmetric and symmetric liposomes, demonstrating similar trends for the two proteins in each liposome type (individual data points shown as dots). For ~20% a-DMPG/PC, the folding was not complete (<75% folded) after 15 h and hence a rate constant could not be determined (Methods). Significance levels (left to right): *P = 0.029 and 0.015 in **e** and *P = 0.029 and 0.029 in **f**, determined by permutation testing (Supplementary Table 11). Source data

**Extended Data Fig. 3:**
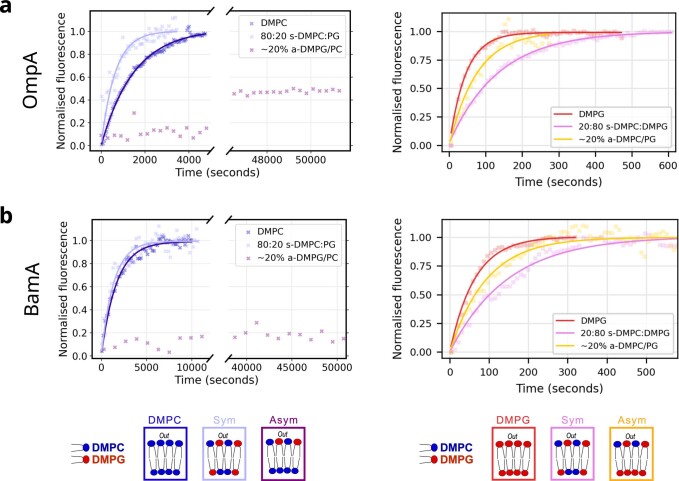
Example kinetic data and fits with representative lipid environments for folding of OmpA and BamA. Sample kinetic data shown for **(a)** OmpA and (**b**) BamA folding into DMPC, DMPG and 20% symmetric and asymmetric liposomes. Data are normalised for comparison, folding into ~20% a-DMPG/PC did not reach completion for either OmpA or BamA and these data were normalised to their respective DMPC traces. Source data

We next studied OMP folding into symmetric and asymmetric bilayers using tryptophan fluorescence (Methods). OmpA is a well-studied model for OMP folding in vitro^57–59^ that contains two domains, an eight-stranded transmembrane β-barrel and a C-terminal (natively periplasmic) water-soluble domain (Extended Data Fig. 1). The water-soluble domain cannot cross the bilayer (thus ensuring unidirectional membrane insertion^60^, confirmed by trypsin cleavage; Supplementary Fig. 7), but has a minimal effect on the folding kinetics of the transmembrane region^57^. This allows the effects of lipid asymmetry on the observed rate of OmpA folding and stability to be determined. Measurements were taken at 30 °C, ensuring that all membranes were in the fluid lipid phase regardless of their composition (Extended Data Fig. 2) and thus have similar mechanical properties^61–63^, although the differential presence of charged lipids will cause small differences^64–66^. Folding kinetics were fitted to a single exponential (Methods) to derive the observed rate constant of folding (Extended Data Fig. 3). As expected^33^, OmpA folds efficiently (folding yield ~79%) into symmetric DMPC liposomes with an observed rate constant (*k*_obs_) of ~0.5 × 10^−3^ s^−1^ (Fig. 2a). The addition of 10% DMPG into both leaflets (that is, maintaining leaflet symmetry) slows folding slightly (40% lower *k*_obs_), while higher (symmetric) concentrations of DMPG accelerate folding (about fivefold higher *k*_obs_ at 40% DMPG; Fig. 2a).

Asymmetric membranes produced strikingly different results. In liposomes containing ≥20% DMPG in their outer leaflets and pure DMPC in their inner leaflet, OmpA failed to fold within 15 h (0.48 M urea; Fig. 2a). By contrast, while OmpA folds more than 40 times more rapidly into symmetric membranes of pure DMPG compared with pure DMPC (Fig. 2a,b), titrating DMPC into the outer leaflet of DMPG liposomes increases the rate constant for folding around twofold relative to symmetric liposomes with equivalent outer leaflet lipid composition at all compositions measured (Fig. 2b). The lipid composition of each leaflet of the bilayer thus affects the rate of OmpA folding. Given the similarity in *T*_m_, area per lipid and acyl chain length of DMPC and DMPG, these effects presumably arise from the different charge of the lipid head groups.

The stability of OmpA in symmetric and asymmetric bilayers was also assessed by cold sodium dodecyl sulfate polyacrylamide gel electrophoresis (SDS–PAGE; Fig. 2c, Methods and Supplementary Fig. 8), which reports on the apparent stability of OMPs within a membrane, in contrast to the kinetic assays reported above, which provide information on the kinetic barrier to membrane insertion (Fig. 2a,b). OmpA is more stable in DMPG liposomes than in DMPC liposomes, as measured by the fraction of unfolded OmpA in a urea titration (urea concentration at the mid-point (half amplitude of curve, *P*_m_) of 4.5 and 2.3 M urea, respectively; Fig. 2c). Similarly to other OMPs^67^, membrane-embedded, native OmpA is resilient to unfolding in 8 M urea in the liposomes studied here (hence equilibrium free energies (Δ*G*°_(eq)_) could not be determined; Fig. 2d and Supplementary Fig. 9). While the addition of small amounts (20%) of DMPC into the outer leaflet of large unilamellar vesicles (LUVs) of DMPG has little effect on *P*_m_ (4.5 M urea), adding 20% DMPC symmetrically into both leaflets destabilizes the protein (*P*_m_ = 3 M urea). While adding 10% DMPG asymmetrically into the outer leaflet of DMPC liposomes also destabilizes OmpA relative to pure DMPC liposomes (*P*_m_ = 1.3 M urea), a symmetric organization of the same lipid composition has an even greater effect (*P*_m_ ≈ 0.8 M urea; Fig. 2c). These data were confirmed by assessing OmpA folding using tryptophan fluorescence (Supplementary Fig. 10). Thus, membrane asymmetry modulates both the rate of folding and the apparent stability of OmpA: excess DMPG (that is, an excess of negative charge) in the outer leaflet slows folding and decreases *P*_m_, while excess DMPG in the inner leaflet accelerates folding and increases *P*_m_ compared with symmetric liposomes with the same outer leaflet lipid composition.

To determine whether these effects are unique to OmpA, we also studied the 16-stranded OMP BamA, which also has a large (47 kDa) water-soluble domain (Extended Data Fig. 1) that ensures the unidirectional folding of its 43 kDa transmembrane β-barrel (Supplementary Fig. 7). BamA folding into symmetric and asymmetric liposomes showed similar trends to OmpA: DMPG/PC asymmetry slows (or abrogates) folding while DMPC/PG asymmetry accelerates folding relative to symmetric systems with the same outer leaflet composition (Fig. 2e,f and Extended Data Fig. 3).

### Charge effects mediate changes in OMP folding and stability

**Extended Data Fig. 4:**
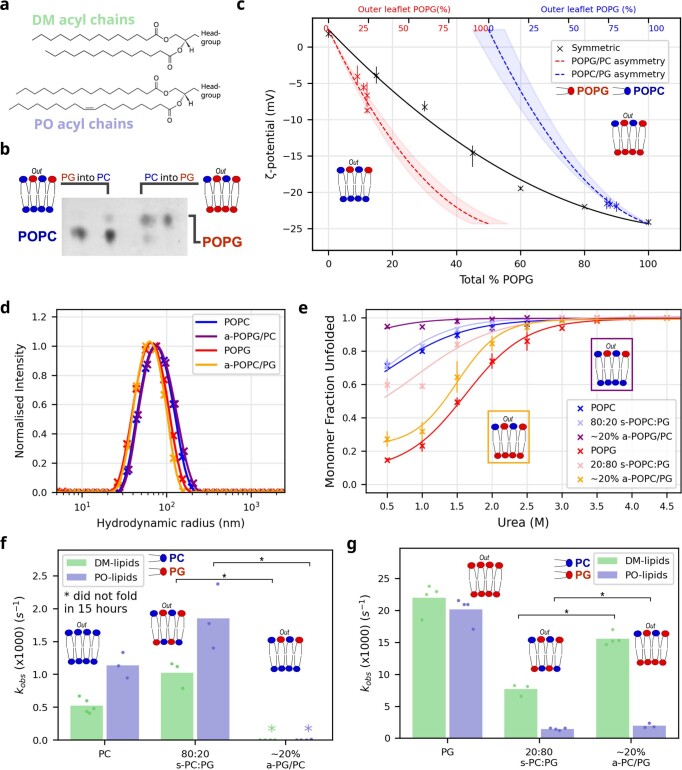
Generating and folding OmpA into POPG-POPC symmetric and asymmetric liposomes. **(a)** Acyl-chain structure of DM- and PO-lipids. **(b)** Sample TLC plate showing the introduction of POPC lipid into POPG liposomes and *vice versa*, as indicated. Outer two lanes, POPC (left) or POPG (right) liposomes before exchange; inner two lanes, exchanging POPG into POPC liposomes (left) or POPC into POPG liposomes (right). **(c)** Experimentally measured ζ-potential calibration curve for asymmetric and symmetric POPC-PG lipid mixes, showing symmetric (black crosses) and asymmetric liposomes (red and blue crosses). Error bars are data range (n ≥ 3). **(d)** Liposome size measured by DLS. **(e)** Urea dependence of OmpA folding into POPC-PG symmetric and asymmetric liposomes. POPG and a-POPC/POPG are fitted to the average of two repeats, all other lines are to guide the eye only as there is insufficient amplitude to enable a fit (bars show the data range of two repeats). **(f, g)** Observed folding rate constant (s^−1^) of OmpA into asymmetric and symmetric liposomes made of DM- or PO-acyl chained lipids, as indicated, demonstrating similar trends to those using DM-lipids for all membrane types (compare with Fig. 2e,f). * Indicates the folding was not complete (<75% folded) in 15 hours and hence a rate constant could not be determined. Significance labels (*) p-values = 0.029, determined by permutation testing (see Supplementary Table 11). Source data

To determine whether these effects of lipid asymmetry on OMP folding and stability are unique to DM lipids, we generated stable asymmetric palmitoyl-oleoyl-phosphatidylglycerol/phosphatidylcholine (POPC-PG) liposomes and found that the folding rates and stability show the same trends as the DMPC-PG lipids, although the magnitudes differ (Extended Data Fig. 4), thus these effects are independent of acyl chain length.

**Fig. 3:**
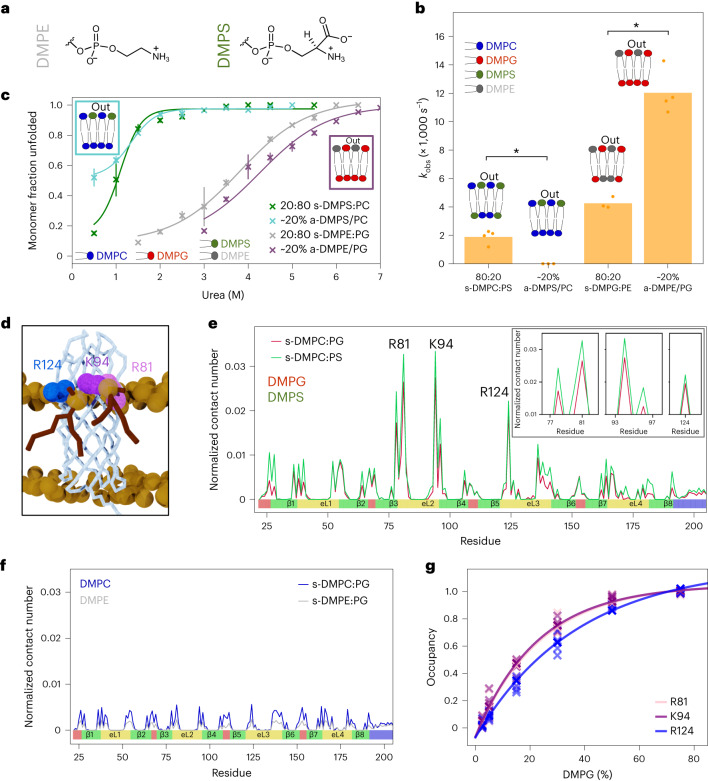
OmpA–lipid charge interactions modulate OmpA folding kinetics and efficiency. **a**, Structures of the DMPE and DMPS head groups, charge analogues of DMPC and DMPG. The same colour code is used throughout. **b**, OmpA folding rate constants (s^−1^) into a-DMPS/PC or a-DMPE/PG LUVs and the equivalent symmetric liposomes (with the same outer leaflet content). For ~20% a-DMPS/PC, the folding had not reached completion after 15 h (<75% folded). Significance levels: *P = 0.029, determined by permutation testing (Supplementary Table 11). **c**, Urea dependence of OmpA folding into DMPS/PC or DMPE/PG symmetric and asymmetric liposomes. The lines are fits to the average of at least two repeats; the bars represent the data range. For 20% a-DMPS/PC, the line has been added to guide the eye, but the amplitude change was too low to accurately fit. **d**, Final frame of a CG-MD simulation of native OmpA in s-DMPG:DMPC membranes, showing two DMPG molecules (red) in the outer leaflet interacting with OmpA at Arg81, Lys94 and Arg124. **e**, Normalized contact count (number of interactions between each type of lipid and each protein residue normalized by lipid concentration and simulation frame number) between residues in the transmembrane region of OmpA and the negatively charged lipids DMPG or DMPS. **Inset:** expanded views of the peaks around the three lipid-interacting residues Arg81, Lys94 and Arg124. **f**, Normalized contact count for interactions between the transmembrane region of OmpA and the zwitterionic lipids DMPC or DMPE. The contact numbers are averages of five replicates. In **e** and **f**, the secondary structure of the OmpA β-barrel is shown below the contact count (green, strands; yellow, extracellular loops; red, intracellular turns; blue, 14 residues of the periplasmic soluble domain). **g**, DMPG occupancy (fraction of time that DMPG interacts with Arg81, Lys94 or Arg124) at different ratios of DMPC:DMPG, determined from the lipid residence time. The data for five replicates are shown. Source data

**Extended Data Fig. 5:**
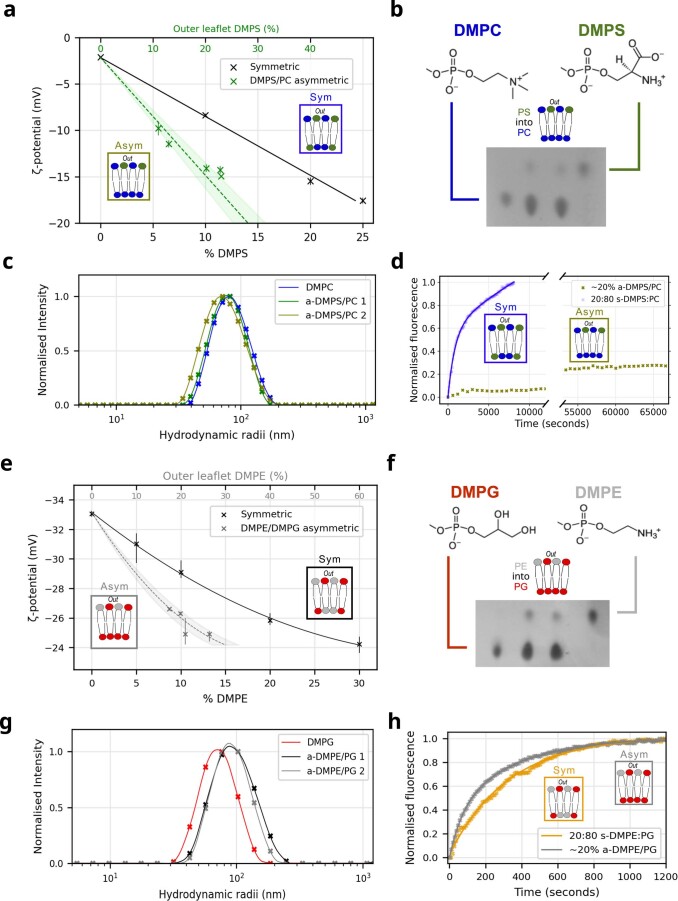
Generating asymmetric DMPS/DMPC and DMPE/DMPG liposomes. **(a)** Experimentally measured ζ-potential calibration curve for symmetric (black) and asymmetric DMPS-DMPG lipid mixes (green) for DMPS concentrations in the outer leaflet of 0–25%. Error bars are data range (n ≥ 3). **(b)** TLC of duplicate ~20% a-DMPS/DMPC exchanged (central two samples) and DMPC (left) or DMPS (right) liposomes. **(c)** DLS of pre-exchange DMPC and duplicate post-exchange DMPS/DMPC liposomes. **(d)** Example OmpA folding kinetic traces, measured by tryptophan fluorescence into 20% s-DMPS:DMPC (double exponential kinetic fit, blue line) and ~20% a-DMPS/DMPC (note that the latter sample did not complete folding (<30% folded over >15 hours, not fitted). **(e)** Experimentally measured ζ-potential calibration curve for asymmetric DMPE-DMPG lipid mixes (grey) for DMPE fractions 0–30% in the outer leaflet. Data for symmetric liposomes are shown in black. Error bars are data range (n ≥ 3). **(f)** TLC of duplicate ~20% a-DMPE/DMPG exchanged liposomes (central two lanes), with DMPG (left) and DMPE (right) unexchanged liposomes. **(g)** DLS of pre-exchange DMPG and duplicate post-exchanged a-DMPE/DMPG liposomes. **(h)** Example OmpA folding kinetic traces, measured by tryptophan fluorescence into 20:80 s-DMPE:DMPG (kinetic fit: yellow line) and ~20% a-DMPE/DMPG liposomes (kinetic fit: grey line). Source data

**Extended Data Fig. 6:**
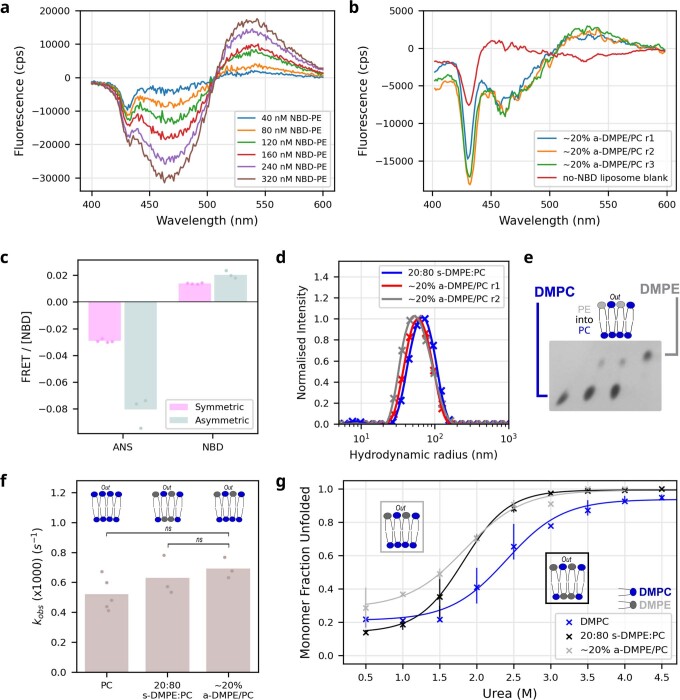
Folding OmpA into symmetric and asymmetric DMPE/DMPC liposomes. **(a)** Background subtracted FRET signal spectrum between BSA-ANS (bovine serum albumin (BSA) bound to the fluorescence donor aniline-naphthalene sulphonate (ANS)) and increasing concentrations of NBD-DPPE lipid (NBD: 7-nitro-2-1,3-benzoxadiazol-4-yl amino, a fluorescent acceptor) in DMPC liposomes (377 nm excitation). **(b)** Background subtracted FRET signal spectrum between BSA-ANS and NBD-DPPE asymmetrically incorporated into the outer leaflet of liposomes and control liposomes (no NBD-DPPE included), indicating clear FRET in the asymmetric liposomes. **(c)** Absolute differences between the fluorophore concentration normalised FRET signals of symmetric and asymmetric liposomes. The larger NBD signal in the asymmetric liposomes is consistent with more of the fluorophore being in the external leaflet and thus closer to the FRET donor ANS bound to BSA in solution. **(d)** DLS of symmetric and two repeats of asymmetric DMPE-PC liposomes with 20% DMPE incorporation. **(e)** TLC of duplicate ~20% a-DMPE/PC exchanged liposomes (central two lanes) with unexchanged DMPC (left) and DMPE (right) liposomes. **(f)** Observed rate constants (s^−1^) of OmpA folding into symmetric and asymmetric DMPC-PE membranes, showing there are no significant differences in symmetric and asymmetric membranes (labelled ns, see Supplementary Table 11). **(g)** Urea dependence of OmpA folding yield into DMPE-PC symmetric and asymmetric liposomes. The data are fitted to the average of two repeats (bars are the data range). Source data

We next studied whether the lipid asymmetry effects were charge-mediated or specific to the PC and PG lipids by folding OmpA into membranes containing DMPS and DMPE (Fig. 3a). Like DMPG, DMPS has a net negative charge, while DMPE, like DMPC, is net neutral. DMPS and DMPE were used at low concentrations (<20%) with DMPG or DMPC to ensure that membranes were in a fluid lipid phase (confirmed by laurdan fluorescence^68^; Extended Data Fig. 2). Asymmetric DMPS/PC and DMPE/PG LUVs were prepared and validated by ζ-potential, TLC and DLS (Extended Data Fig. 5). The kinetics of OmpA folding into a-DMPS/PC shows that the addition of DMPS into the outer leaflet of DMPC liposomes retards folding, akin to a-DMPG/PC (compare Fig. 3b with Fig. 2e). Unlike DMPG/PC lipid mixes, the stability of inserted OmpA is similar in the DMPS/PC symmetric and asymmetric membranes (Fig. 3c and Supplementary Fig. 11a,b). Asymmetric DMPE/PG also mimics the effects of a-DMPC/PG lipid mixtures, with the addition of DMPE to the outer leaflet of DMPG liposomes accelerating folding and stabilizing the inserted protein relative to symmetric DMPE:PG liposomes of equivalent outer lipid composition (compare Fig. 3b,c with Fig. 2c,f). As a final control, stable, charge-similar but head group-dissimilar DMPE/DMPC liposomes were generated and their asymmetry validated using a fluorescence resonance energy transfer (FRET)-based assay (stable DMPS/DMPG liposomes could not be generated; Extended Data Fig. 6a–e and Methods). No difference in OmpA folding kinetics or urea stability was observed using symmetric and asymmetric DMPE/PC liposomes (Extended Data Fig. 6f,g), indicating that lipid asymmetry alone does not modulate OMP folding, whilst asymmetry in charge has a dramatic effect.

### Extracellular loops of OmpA interact with negative lipids

**Extended Data Fig. 7:**
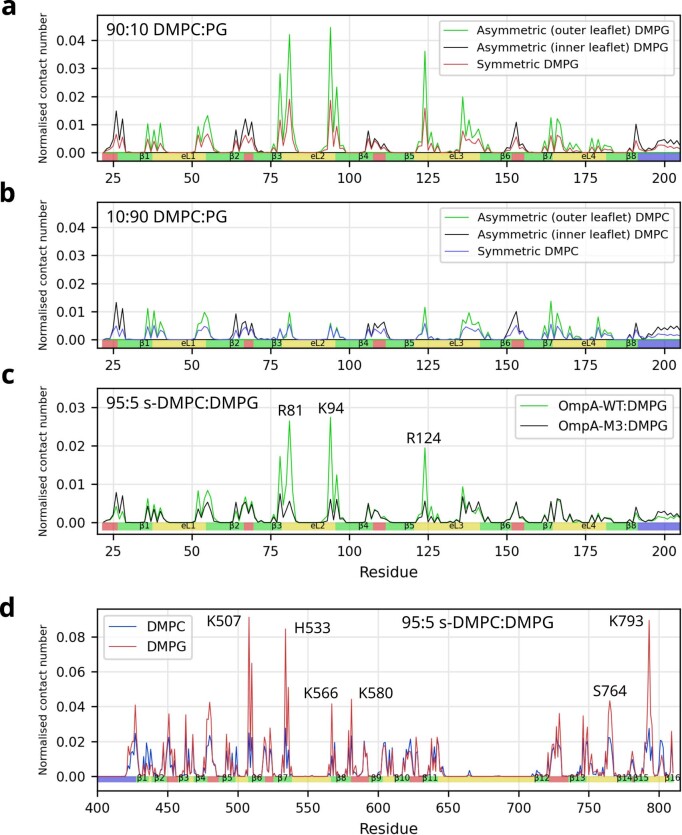
Lipids in the outer leaflet interact with specific residues in OMP loops in simulations of symmetric and asymmetric membranes. Normalised lipid-protein contact counts (number of interactions between each type of lipid and each residue in the transmembrane domain of OmpA normalised by lipid concentration and simulation frame number) for a total of 10% symmetrically or asymmetrically distributed lipid, as indicated in the legend for **(a)** DMPG (in DMPC base membranes) and **(b)** DMPC (in DMPG base membranes). The total lipid composition, indicated on each panel, was the same in both symmetric and asymmetric membranes, only protein-lipid interaction data for the 10% supplemented lipid is shown. The data show that DMPG interacts with OmpA’s R81, K94 and R124 in the outer leaflet of asymmetric and symmetric membranes. **(c)** Normalised contact number for the transmembrane region of OmpA-WT and OmpA-M3 in a 95:5 s-DMPC:PG membrane. Substitution of these three positive residues with Ser eliminates specific DMPG binding. **(d)** Full length BamA was simulated in a 95:5 s-DMPC:PG system. Only the transmembrane region is shown for clarity. Interactions with a normalised contact number >3σ are labelled, and indicated in main text. Structural features are shown at base of plot (strands (green), extracellular loops (yellow) intracellular turns (red) and 24 residues from POTRA5 (blue)). Source data

OMPs commonly contain positive residues in their extracellular loops, which must cross the bilayer for the protein to achieve its native fold, where their interactions with lipid head groups could stabilize the native state. To identify residues that might engage in such stabilizing interactions, we used coarse-grained molecular dynamics (CG-MD) to explore the interplay between membrane asymmetry, lipid head groups of different charge and charged residues in the extracellular loops of natively folded, membrane-embedded OmpA (Supplementary Table 1). Natively folded OmpA was placed in different membranes and the systems were minimized, equilibrated (Extended Data Fig. 1, inset) and simulated in five replicas of 3 µs each in all systems. The membrane and protein properties were assessed to ensure equilibration and stable simulations (Supplementary Figs. 12–17 and Methods). For each system, the number of contacts between the different lipids and each residue of the protein were calculated and normalized by the lipid concentration and simulation time to facilitate comparison. This analysis identified specific interactions between the head groups of DMPG and DMPS and three positively charged residues (Arg81, Lys94 and Arg124) in the extracellular loops of OmpA (Fig. 3d,e). No such interactions were found with DMPC or DMPE (Fig. 3f), further evidenced by calculating the average lipid density around the protein (Supplementary Fig. 18). In silico mutation of these three residues to serine removed these interactions (Extended Data Fig. 7c). Calculation of the average occupancy time of the lipid at each site^69^ showed that the interaction time of DMPG with Arg81, Lys94 and Arg124 also depends on the DMPG concentration (Fig. 3g). Similar lipid–protein interactions were found in the simulations of OmpA in asymmetric DMPC-PG membranes (Extended Data Fig. 7a,b). Simulations of natively folded BamA in s-DMPC:PG membranes also showed specific interactions between DMPG and residues Lys507, His533, Lys566, Ser764 and Lys793 in its extracellular loops (and Lys580 in an intracellular turn; Extended Data Fig. 7d). These results suggest that charge-mediated lipid–protein interactions involving the extracellular loops of OMPs could play a role in stabilizing membrane-embedded OMPs in their natively folded states and, thereby, contribute to the favourable driving force for OMP folding.

### Lipid–OmpA loop charge interactions modulate folding

**Fig. 4:**
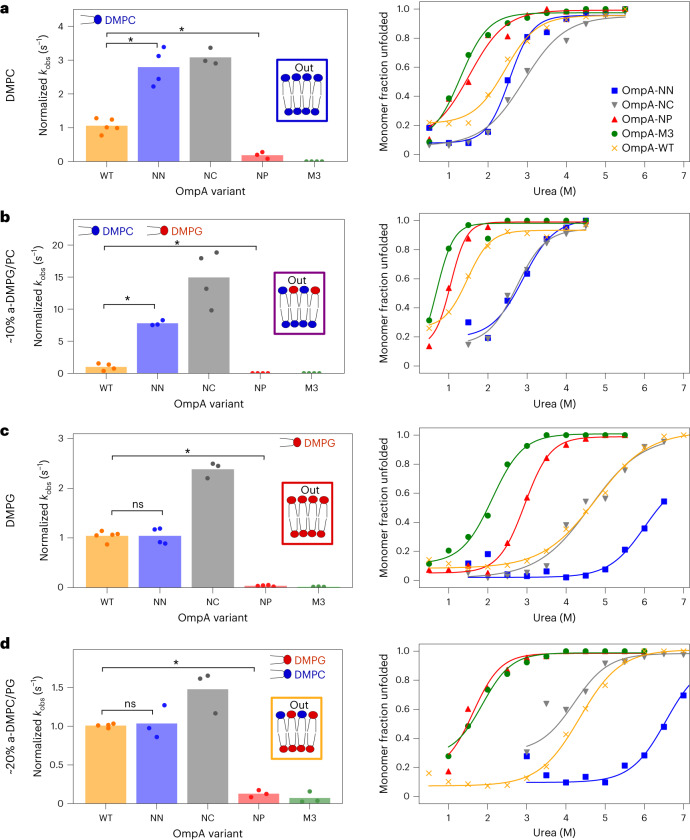
Folding kinetics and stability of OmpA charge variants compared with OmpA-WT for symmetric and asymmetric lipid environments. **a**–**d**, The relative folding rate constants (normalized to WT) (left) and urea titration stability curves (right) measured using cold SDS–PAGE for OmpA variants in DMPC (significance levels: WT–NN, *P = 0.008; WT–NP, *P = 0.018) (**a**), ~10% a-DMPG/PC (the fits for OmpA-NP and OmpA-M3 in urea are included to guide the eye, but the stability was too low to accurately fit the data; significance levels: WT–NN, *P = 0.029; WT–NP, *P = 0.014) (**b**), DMPG (significance levels: WT–NN, ns*P* = 1.0; WT–NP, *P = 0.008) (**c**) and ~20% a-DMPC/PG (significance levels: WT–NN, ns*P* = 0.829; WT–NP, *P = 0.029) (**d**). In **a** and **b**, the folding of OmpA-M3 (**a**) and OmpA-NP and OmpA-M3 (**b**) had not reached completion after 2 h (<75% folded). The OmpA-NC fraction folded at 3.5 M urea was excluded from the fit in **d**. All *P* values were determined by permutation testing (see Supplementary Tables 2–8 for the *P* values of the comparisons described in the text, and Supplementary Tables 11 and 12 for all pairwise tests of significance); ns, no significant difference. Source data

**Extended Data Fig. 8:**
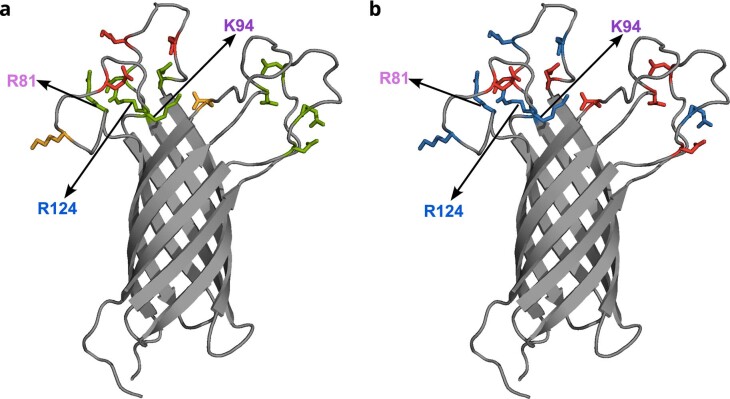
Conservation and location of Lys/Arg positively and Asp/Glu negatively charged residues in the extracellular loops of OmpA. **(a)** Relative conservation of charged residues (green: well conserved (>99%), yellow: partially conserved (>90%), red: less conserved (<90%)) over 2750 OmpA β-barrel sequence homologs. Conservation is the retention of a K/R or D/E at a given position. Note that R81, K94 and R124 are highly conserved (but are not the only highly conserved residues in the loops). **(b)** Spatial distribution of positively (Lys/Arg) and negatively charged (Glu/Asp) residues in the extracellular loops of the NMR structure of OmpA (blue: positive, red: negative). R81, K94 and R124 that specifically interact with negatively charged lipids are labelled. (PDB: IG90, note that the z-axis locations of R81, K94 and R124 are highly consistent across all solved structures, including those solved by NMR and X-ray crystallography (Extended Data Fig. 10c). Source data

**Extended Data Fig. 9:**
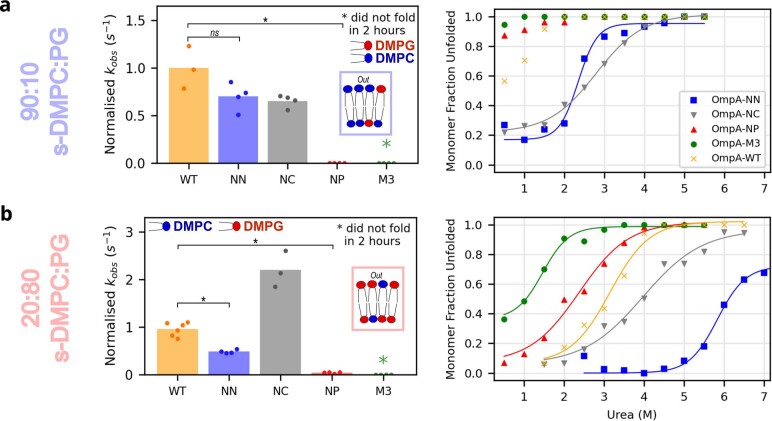
Folding kinetics and urea-titration of OmpA charge variants compared with WT OmpA in symmetric 90:10 or 20:80 s-DMPC:DMPG LUVs. The difference in folding rate constants (normalised to WT OmpA) and apparent stability vs. urea concentration for OmpA-variants in symmetric LUVs of composition **(a)** 90:10 s-DMPC:PG (p-values: WT-NN: 0.086, WT-NP: 0.029) and **(b)** 20:80 s-DMPC:PG (p-values: WT-NN: 0.005, WT-NP: 0.005), as indicated in the key. (All p-values determined by significance testing, see Supplementary Tables 2–8 for p-values of comparisons described in the text, and Supplementary Table 11 and 12 for all pairwise tests of significance). Source data

The extracellular loops of natively folded OmpA contain seven positively charged residues (Arg81, Lys85, Lys94, Arg124, Lys128, Lys134 and Arg177) and seven negatively charged residues (Asp41, Glu53, Glu89, Asp126, Asp137, Asp170 and Asp179). Many of these residues are highly conserved (Extended Data Fig. 8), including the three lipid-interacting residues (Arg81, Lys94 and Arg124) identified by CG-MD above. To investigate the role of OMP loop–lipid charge interactions experimentally, four variants of OmpA that differ in their extracellular loop charge were created: OmpA-NP (no positives, loop charge −7), OmpA-NN (no negatives, loop charge +7), OmpA-NC (no charges) and OmpA-M3 (three mutants, namely R81S, K94S and R124S; see Methods for sequences). The folding rate and apparent stability of these variants folding into symmetric and asymmetric DM liposomes was then determined (Fig. 4 and Extended Data Fig. 9).

These experiments revealed that translocating positively or negatively charged loops of OmpA across a bilayer constitutes a major barrier to folding irrespective of the charge orientation of the bilayer. Thus, OmpA-NC folds more rapidly than wild-type OmpA (OmpA-WT), OmpA-NP and OmpA-NN in the majority of bilayers tested (Fig. 4a–d, Extended Data Fig. 9, left, and Supplementary Table 2). However, and importantly, given that OMP extracellular loops typically contain charged residues, the presence of positive charge favours rapid folding compared with its absence (that is, OmpA-WT and OmpA-NN fold more rapidly than OmpA-NP in all lipid types; Fig. 4a–d, Extended Data Fig. 9, left, and Supplementary Table 3). Indeed, OmpA-NN folds more than nine times more rapidly than OmpA-NP in all bilayer types. Neutralization of the three lipid-interacting OmpA-M3 positive residues retards folding to a similar extent as neutralizing all seven positively charged residues (OmpA-NP; Supplementary Table 4), demonstrating the key importance of these three residues in folding kinetics.

Similar trends were observed for protein stability. An excess of loop negative charge destabilizes OmpA-NP compared with OmpA-WT (Supplementary Table 5), and an excess of positive charge stabilizes OmpA-NN compared with OmpA-WT in all lipid environments (Fig. 4a–d, Extended Data Fig. 9, right, and Supplementary Table 6). Again, OmpA-M3 mirrors the behaviour of OmpA-NP (Fig. 4a–d, Extended Data Fig. 9, right, and Supplementary Table 7). Switching loop charge can also have different effects on the folding rate and apparent stability. For example, OmpA-NN and OmpA-WT fold at similar rates in DMPG-rich membranes (Fig. 4c,d, left), but OmpA-NN is significantly more stable (Fig. 4c,d, right, and Supplementary Table 8), likely due to favourable electrostatic interactions with the negatively charged lipid. Collectively, these results highlight the importance of the positively charged loop residues in facilitating the translocation of OmpA across the bilayer and then stabilizing the native protein once folded into the membrane. For OmpA, this effect is dominated by the three, highly conserved, M3 residues.

**Fig. 5:**
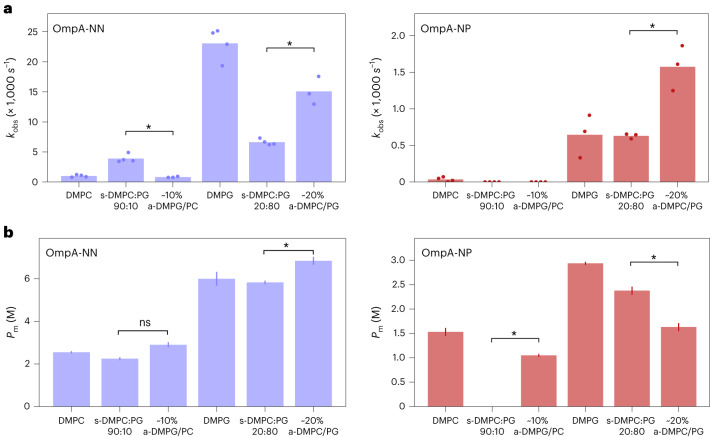
Folding kinetics and stability of OmpA-NN and OmpA-NP. **a**, Non-normalized folding rate constants for OmpA-NN (left) and OmpA-NP (right) into symmetric and asymmetric liposomes, demonstrating the different patterns of folding rate observed for the different OmpA charge variants. The folding of OmpA-NP had not reached completion after 2 h in the s-DMPC:PG 90:10 and a-DMPG/PC liposomes (<75% folded). Note the different *y*-axis scales in the two plots. Significance levels: *P = 0.029, determined by permutation testing (Supplementary Table 9). **b**, *P*_m_ values for the folding of OmpA-NN (left) and OmpA-NP (right) into symmetric and asymmetric liposomes in urea solutions. There was insufficient folding of OmpA-NP into 90:10 s-DMPC:PG to allow a fit. The error bars represent the goodness of fit to the data shown in Fig. 4 (the standard deviation of the *P*_m_ values was estimated from the covariance of fitted parameters); the bar heights are the fitted parameter values. Significance levels (from left to right): ns*P* = 0.423) and *P = 0.020 for OmpA-NN, and *P = 0.031 and 0.016, determined by a two-tailed paired *t*-test (Supplementary Table 10). Source data

Finally, the folding kinetics and urea stability of OmpA-NN and OmpA-NP were directly compared in symmetric and asymmetric DMPC-DMPG bilayers (Fig. 5). OmpA-NN folds more rapidly into 90:10 s-DMPC:PG membranes than into ~10% a-DMPG/PC, in which only the outer leaflet of the bilayer contains the negatively charged lipid (Fig. 5a, left, and Supplementary Table 9). This suggests a rate-enhancing interaction between the protein positive loops and the negative charge of DMPG in the inner leaflet of the bilayer. By contrast, OmpA-NP folds very slowly into both of these membrane types (Fig. 5a, right, and Supplementary Table 9). For both OmpA-NN and OmpA-NP, folding is faster into ~20% a-DMPC/PG than its symmetric counterpart (s-DMPC:PG 20:80), although overall folding is more rapid for OmpA-NN as it contains positively charged loops (Fig. 5a). Bilayer lipid charge asymmetry also affects stability (Fig. 5b). For example, OmpA-NP is more stable in s-DMPC:PG (20:80) than in its asymmetric bilayer counterpart, while the protein is less stable in s-DMPC:PG (90:10) than in a-DMPC/PG (Fig. 5b, right, and Supplementary Table 10). However, OmpA-NN is more stable in ~20% a-DMPC/PG than in the equivalent symmetric membranes, while OmpA-NP shows the opposite effect (Fig. 5b and Supplementary Table 10). While many details of the complex interplay between lipid charge asymmetry and OMP loop charge remain to be determined, these data unambiguously show that the efficient folding and stability of OmpA depend on positive charges in its extracellular protein loops and charge asymmetry between the two leaflets of the target bilayer.

### OMPs have a conserved patch of extracellular positive charge

**Fig. 6:**
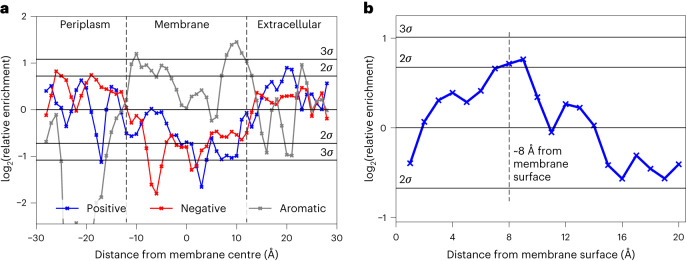
Localised positive charge enrichment of OMP residues. **a**,**b**, OMP residue enrichment perpendicular to the membrane plane shows conserved enrichment of positively charged residues in the extracellular loops ~8 Å from the membrane surface. **a**, Residue enrichments of aligned OMPs from the OPM database (experimentally solved structures) relative to the probability of finding an amino acid randomly, calculated over the whole protein sequence. The membrane thickness is the average of all OPM structures. **b**, Residue enrichments of Lys and Arg in the extracellular loops of OMPs relative to the probability of finding an amino acid from the soluble regions of the protein sequence, calculated from proteins in the OPM database (that is, transmembrane residues have been omitted from this analysis). Source data

**Extended Data Fig. 10:**
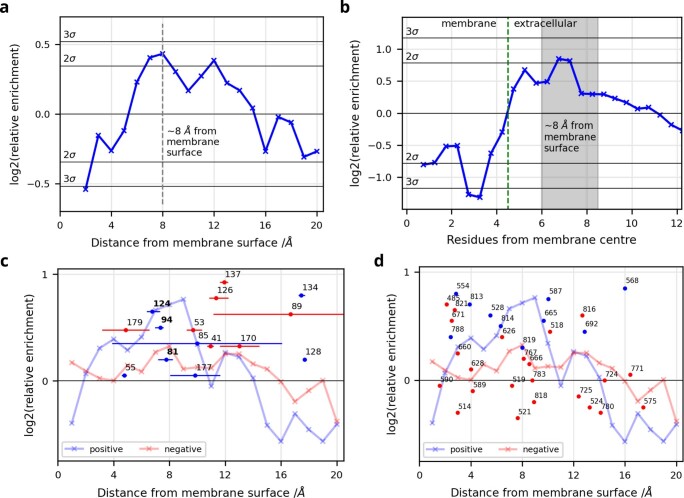
Predicted structures from Alphafold2 and sequences from the OMPdb also show a positive region ~ 8 Å from the membrane that is observed in the charged residues in the extracellular loops of OmpA and BamA. Residue enrichments of Lys/Arg residues in OMPs relative to the probability of finding an amino acid from the soluble regions of the protein randomly for **(a)** predicted structures from the Alphafold2 database, and **(b)** sequence data from the OMPdb (see Supplementary Fig. 19 for the residue count from the membrane centre to approximate distance calibration). The dashed green line in (**b**) indicates the approximate membrane hydrophobic-hydrophilic boundary. **(c)** OmpA and **(d)** BamA charge distribution matches the bioinformatic profile. Residues R81, K94 and R124 in OmpA, identified as lipid interacting by CG-MD (OmpA-M3 cluster), are shown in bold. OmpA distances are calculated as the average of the solved *E*. coli OmpA structures (PDB 1G90, 1QJP, 1BXW). Error bars indicate maximum and minimum values. Positive residues are shown as blue circles, negative residues as red circles and are labelled with residue number above-right of the marker. Source data

The enrichment of positive charges in the extracellular loops of OMPs has been noted previously^41^. We further examined the distribution of positively charged residues in OMPs in the Orientations of Proteins in Membranes (OPM) database^70^ (Methods). The spatial enrichment of different residues was calculated in 1 Å slabs parallel to the membrane plane, identifying the well-characterized OMP aromatic girdle^41^ that flanks the acyl chains on each side of the membrane (Fig. 6a). Patterns of charged residue distributions were not obvious from this analysis, presumably because different residue probabilities in the transmembrane and water-soluble regions of the protein skew the statistics. The analysis was therefore repeated with transmembrane residues excluded (Fig. 6b), revealing a patch of (>2*σ* significant) positive residues 6–10 Å above the plane of the membrane’s outer leaflet, precisely matching the location of OmpA’s M3 residues (Extended Data Fig. 10).

While the OPM database is rich in information, it contains relatively few OMPs. We therefore interrogated OMP structures predicted by AlphaFold2 (refs. ^71,72^) and sequences from the OMPdb^73^ database. Quality filtering and sequence clustering yielded 343 AlphaFold2 structures and 19,055 OMPdb sequences of transmembrane OMPs. While sequence data lack explicit structural information, approximate distances can be estimated using the residue count from the membrane centre (Methods and Supplementary Fig. 19). These analyses also show a peak (≥2*σ*) for enrichment of positive residues at ~8 Å from the membrane surface (Extended Data Fig. 10a,b). No consistent pattern was observed for negatively charged residues (Supplementary Fig. 19). Collectively, these analyses identify an enrichment of positive residues in the extracellular loops at ~6–10 Å from the membrane surface. Given how strongly the M3 set of positive charges impact the folding of OmpA, we suggest that the conservation of these charges facilitates efficient OMP folding into the OM.

## Discussion

More than 35 studies on the folding kinetics of 15 different OMPs have been published over the last 30 years (reviewed in refs. ^25,28^). Despite this extensive literature and the fact that lipid asymmetry between the two leaflets of a bilayer is the norm for biological membranes, very little is known about its implications for OMP folding or stability. Here, we have described a systematic study of OMP folding into asymmetric bilayers. The results are striking, showing that lipid asymmetry has a profound effect on both the observed rates of folding and the apparent stability of the protein in the bilayer. This effect is mediated by charge distribution. Increasing the number of negatively charged lipid head groups (DMPG or DMPS) in the inner leaflet of the liposome (functionally equivalent to the outer leaflet of the OM), progressively reduces the kinetic barrier for folding and thus increases its rate. However, when negatively charged lipids are present only in a liposome’s outer leaflet (equivalent to the inner leaflet of the OM), stabilizing lipid head-group interactions with the natively folded protein loops in the inner leaflet cannot occur, with the result that both OmpA and BamA fold poorly. In addition to altered protein–lipid interactions, folding may be modulated by changing the mechanical properties of membranes^31^. While in symmetric membranes charged lipids decrease global membrane stiffness due to electrostatic repulsion^64–66^, offering a possible explanation for why higher fractions of DMPG facilitate easier protein insertion, the effects of asymmetric charge distribution are unclear^66,74^, and this merits further study. Regardless of the exact underlying physical phenomena, the interplay between protein and lipid charge distribution has major consequences for OMP folding and stability.

The results from liposomes composed of DMPE and DMPG are particularly interesting, as these are the dominant lipids in the inner leaflet of bacterial OM^75^. The symmetric incorporation of DMPE into DMPG liposomes slows folding, consistent with symmetric DMPC/DMPE mixes^33^. However, we have shown that this inhibition can be partially overcome by introducing DMPE asymmetrically into only the liposome outer leaflet. Thus, the reduction in folding rate is mediated by the types of lipid in both leaflets, with the balance of negative charge across the two leaflets forming a rheostat that tunes the folding rate by more than three orders of magnitude in the conditions sampled in this study. In a biological context, our results suggest that the asymmetric presence of negatively charged LPS in the bacterial OM could directly facilitate folding, with the rate being modulated by lipids in the inner leaflet of the OM, as well as the presence of BAM (β-barrel assembly machinery) and other folding factors. Interestingly, the inner membrane (IM) of canonical rod-shaped *Escherichia coli* is also asymmetric, with an approximately threefold excess of PE in the leaflet facing the cytoplasm compared with the periplasm-facing leaflet^76^. This excess of a neutral lipid head group in the inner leaflet would create an effective excess of negative charge on the periplasmic leaflet, which would disfavour aberrant folding of OMPs into the IM and hence could play a role in determining the flux of OMPs into the OM.

Protein charge interactions are known to play a role in folding^37^, and the ‘positive-outside’ rule, first described in 2005 (ref. ^40^), is a well-recognized feature of OMP sequence/structure. Here, we have revealed the molecular detail that underpins this phenomenon. Using MD simulations and mutational analysis, we have identified that a patch of external positive (PEP) residues in the loops of OmpA is critical for productive folding, rather than a general requirement for positive charge. These PEP residues lie at ~6–10 Å from the membrane surface and mediate OMP folding via interactions with the excess negative charge that we have identified above as being a key driver for efficient folding. Using bioinformatics, we have shown that the PEP is a generic feature of OMP sequences, suggesting that it may be a conserved determinant of efficient OMP folding. For the studies in liposomes, the excess negative charge is in the inner leaflet, but in the native OM, the protein would approach the membrane from the periplasm, and excess negative charge, particularly on LPS molecules, would be in the outer leaflet of the OM. Thus, the natural selection of OMP sequences and the machinery for the generation and maintenance of lipid asymmetry might plausibly operate synergistically to maximize the efficiency of OMP folding.

In summary, these results provide new insights into how bilayer charge asymmetry affects the folding and stability of OMPs. Specifically, we have revealed charge-mediated features in both the lipid environment and protein sequences that reduce the kinetic barrier to OMP folding and stabilize the final, membrane-inserted state. Although the exact nature of the modulation and its interplay with other parameters that might modulate folding, such as the membrane’s mechanical properties, will require further studies of a broad range of OMPs, including lipid mixes incorporating LPS, the results suggest routes to manipulate OMP behaviour for biotechnology applications, how bacteria might exploit lipid asymmetry to modulate the efficiency of OMP folding into the highly asymmetric OM and, more broadly, how cells might exploit lipid asymmetry to modulate the efficiency of folding of their membrane proteins.

## Methods

### Liposome preparation

DMPC (dimyristoyl-phosphatidylcholine), DMPG (dimyristoyl-phosphatidylglycerol), DMPE, (dimyristoyl-phosphatidylethanolamine) DMPS (dimyristoyl-phosphatidylserine), POPC (palmitoyl-oleoyl-phosphatidylcholine) and POPG (palmitoyl-oleoyl-phosphatidylglycerol) lipids (Avanti Polar Lipids) were prepared as stock solutions with concentrations of 25 mg ml^−1^ in chloroform. Liposome preparations were all made to ~40 mM lipid concentration. Lipids were placed in amber glass vials and dried under N_2_, vacuum desiccated for >3 h and resuspended in buffer (10 mM Tris-HCl, 100 mM NaCl, pH 8.5). Following complete resuspension, samples were freeze-thawed five times in liquid N_2_ and a 42 °C water bath, then extruded 31 times through 100 nm nucleopore polycarbonate track-etched membranes (Whatman, GE Healthcare and Avanti extruder) at a temperature ~10 °C higher than the *T*_m_. DMPE lipids were sonicated rather than extruded. DPPE-rhodamine (1% (mol/mol), Avanti) was introduced as a fluorescent label where indicated. Liposomes were used within 48 h of their synthesis. The lipid concentrations of DMPC and DMPG liposomes were determined by absorbance, calibrated by the Stewart assay^77^: samples were dissolved in 750 μl chloroform, to which 750 μl guanidine ferric thiocyanate was added (0.4 M guanidine thiocyanate and 0.1 M iron(III) chloride hexahydrate). Samples were vortexed vigorously for 1 min. Following phase separation, the chloroform phase was removed with an 18-gauge needle, its absorbance at 448 nm measured and lipid concentration determined from the calibration prepared (Supplementary Fig. 20).

### Lipid exchange

The following protocol was adapted from a previous publication^19^. Concentrations of the donor (Cd) and acceptor (Ca) lipids were determined by:

1 $${\rm{Cd}}=a\times {\rm{Ca}}\times {\rm{asym}}/(1-{\rm{asym}})$$

where *a* is the fraction of lipid accessible (~0.5) and asym is the desired asymmetry (up to about 0.5). The concentration of MβCD (Cm) was determined by:

2 $${\rm{Cm}}=n\times {\rm{Cd}}+({\rm{Cd}}\times K)\times 1/n;$$

where *n* (set as 4) is the stoichiometry of the CD–lipid complex and *K* is an experimentally derived value (set as 292 M^−3^ for DMPC, DMPG and DMPS donation, and empirically adjusted to 150 M^−3^ for DMPE donation). These values are sensitive to MβCD activity and phospholipid-specific differences can be substantially reduced by using intermediate MβCD–lipid saturation (fixed at 70%). Donor liposomes (or resuspended lipid for DMPE) were first solubilized with MβCD (Sigma) at 50 °C and 1,000 r.p.m. for >20 min. Acceptor liposomes were then added and incubated at 35 °C and 400 r.p.m. for >20 min to allow for exchange. The liposomes were purified by two rounds of ultracentrifugation (105,000 *g*, 4 °C, 30 min, Beckman Coulter, Optima MAX-XP). Following resuspension, the liposomes were centrifuged at 5,000 *g* for 5 min to remove aggregates. To ensure high sample yields, only a single round of exchange was carried out, limiting asymmetry to ~30% DMPC/PG and ~50% DMPG/PC. The generated asymmetric liposomes were grouped to the nearest 10% (±3%) for analysis. The exchanged liposomes were checked for quality and used the day they were made. Stable symmetric and asymmetric DMPE/PC membranes were successfully created with up to 20% DMPE. Attempts to prepare DMPS/PG liposomes consistently resulted in aggregate formation. As DMPS and DMPE have *T*_m_ values of 35 and 50 °C, respectively^78^, liposomes always had <20% of these lipids to ensure that they remained in the fluid phase.

### Liposome absorbance analysis

Liposome absorbance was measured in the range 300–600 nm using quartz cuvettes. The absorbance traces were deconvoluted using a custom script that found the liposome and fluorophore concentrations that minimized the following function:

3 $$\mathop{\sum }\limits_{\lambda = 300}^{\lambda = 600}{({A}_{{\mathrm{reconvoluted}}}-{A}_{{\mathrm{raw}}})}^{2}$$

where *λ* is the wavelength, *A*_raw_ is the raw absorbance trace and *A*_reconvoluted_ is the theoretical absorbance from the deconvoluted data, using reference spectra of the fluorophore alone and unlabelled liposomes.

### Determination of MβCD concentration using anthrone

Each sample (30 μl) was mixed with 100 μl anthrone reagent (0.2% (w/w) anthrone in 50% (v/v) H_2_SO_4_), heated at 95 °C for exactly 10 min and then quenched by cooling on ice. The absorbance of the samples was measured at 630 nm. A calibration curve of 0–200 μM MβCD at intervals of 25 μM was measured every time samples were assayed.

### Thin layer chromatography

Liposome samples were diluted to ~0.5–2 mM and 5 µl samples were dried under nitrogen. Each sample was resuspended in chloroform and spotted onto a TLC plate (silica gel 60 F_254_, Sigma, 1.6834) and run with 40:9:6:3 (v/v) chloroform–methanol–ethanoic acid–water (DMPG/DMPC and POPG/POPC), 60:20:1 (v/v) chloroform–methanol–water (DMPG/DMPE) or 130:20:2 (v/v) chloroform–methanol–water (DMPC/DMPS). The plates were dried at 50 °C, dip-stained into phosphomolybdic acid and developed by heating at 200 °C for exactly 20 min. The plates were imaged with a Q9 alliance imaging system (Uvitec) and densitometric analysis was performed using ImageJ.

### ζ-Potential and DLS

ζ-Potentials and DLS were measured on a Zetasizer Nano ZS instrument (Malvern) using DTS1070 cells at 25 °C (60 s incubation), with 10–100 measurements made in a water dispersant. Each sample was measured in triplicate, and cells were cleaned with 2% (v/v) Hellmanex, 18 MΩ H_2_O and then ethanol and finally dried under nitrogen. Cell quality was ensured approximately every five measurements using a reference standard (Malvern, DTS1235).

### Imaging liposomes using cryoEM

Samples (3 µl) of ~0.5 mM liposomes were placed on glow-discharged quantifoil grids (1.2/1.3, PELCO easiGlow, Ted Pella) and incubated for 30 s. The grids were then blotted for 6 s with Whatman no. 1 filter paper at 4 °C and ~90% relative humidity and then plunge-frozen in liquid ethane using a Vitrobot Mark IV System (ThermoFisher). The grids were imaged with a 300 keV Titan Krios electron microscope (ThermoFisher) using EPU software and a K2 detector.

### ζ-Potential prediction model

A review of the literature^19,44–56^ combined with the data presented here yielded 315 data points that met the following inclusion criteria: (1) the *T*_m_ value of all lipids of each sample must be known (except cholesterol, which was handled separately; any liposomes without defined acyl chain composition were removed), (2) the buffer salt must be NaCl or KCl, and (3) ethanol must not be present in the buffer. The lipid composition of all the liposomes was parametrized by (1) the average overall charge per lipid, (2) the average *T*_m_ of all lipids and (3) the fraction of lipid composition that is cholesterol.

An Extreme Gradient Boosted model (from XGboost library^79^) was used with a root-mean-squared error loss function, a learning rate of 0.05 and an early-stop patience of 25 cycles (as evaluated from the current 25% validation data). The model was trained with the target ζ-potential using eight dataset features: salt concentration (monovalent), salt concentration (divalent), pH, hydrodynamic radii, temperature, overall charge, lipid *T*_m_ and cholesterol fraction. The error associated with each measurement (the standard deviation) was used to weight the features of an individual data point, with the weightings normalized between 0.375 and 0.625. Model hyper-parameters were explicitly optimized to reduce the model overfitting identified in early testing: subsample per node, 0.85; subsample per tree, 0.85; minimum child weight, 2.5; maximum tree depth, 6. The models were validated with fourfold cross-validation. Predictions were made by training a 50 model ensemble (all with MAE < 5 mV) on the fly and averaging their predictions to obtain a final value. The weight or gain per feature was analysed using the Python package scikit-learn^80^.

### DMPE/PC liposome generation and FRET asymmetry assay

DMPC liposomes doped with different concentrations of NBD-DPPE (N-(7-nitro-2-1,3-benzoxadiazol-4-yl)-dipalmitoyl-phosphatidylethanolamine) were generated and their fluorescence spectra measured at excitation/emission wavelengths of 457/530 and 375/530 nm in the absence or presence of BSA–ANS (8-Anilinonaphthalene-1-sulfonic acid) (pre-incubated for 1 h at 37 °C, final concentrations 10 µM BSA and 30 µM ANS). DMPE doped with 1% NBD-DPPE was exchanged into the outer leaflets of DMPC liposomes as described above and the ANS–NBD FRET determined by subtracting the fluorescence spectra from the background spectra (BSA–ANS alone and NBD-DPPE fluorescence excited at 375 nm) and normalized to the concentration of NBD. The FRET of the exchanged samples was substantially greater than the expected symmetric FRET, indicating the retention of asymmetry.

### Plasmids and creation of mutants

Sequence alignment of OmpA homologues identified residue substitutions of charged residues within its extracellular loops. The most common alternative residue was used to generate the OmpA variants, or for residues that are completely conserved, they were replaced with serine. OmpA-NP: R81S, K85T, K94S, R124S, K128G, K134S and R177S; OmpA-NN: D41S, E53N, E89V, D126S, D137S, D180S and D189S; OmpA-NC: a combination of both OmpA-NP and OmpA-NN; OmpA-M3: R81S, K94S and R124S. The genes encoding mutants of OmpA were ordered from GeneWizz, ligated into a pET11a vector using flanking BamHI and NdeI restriction sites, and validated by sequencing.

### Protein expression and purification

Competent BL21(DE3) *E. coli* cells were transformed with the relevant plasmid (carbenicillin-resistant), grown overnight at 37 °C on agar plates, and a single colony was picked and grown overnight in ~20 ml LB (luria broth) containing 100 µg ml^−1^ carbenicillin (37 °C, 200 r.p.m.). Then, 5 ml culture was added to 500 ml LB, grown to an optical density at 600 nm of ~0.6 and protein expression was then induced with 1 mM IPTG (isopropylthiogalactoside). Three hours post-induction, the cells were collected (5,000 *g*, 15 min, 4 °C) and the cell pellet frozen. After thawing, the pellet was resuspended in 20 ml buffer (50 mM Tris-HCl, pH 8.0, 5 mM EDTA, 1 mM phenylmethylsulfonyl fluoride, 2 mM benzamide) and the cells lysed via sonication. Following centrifugation (25,000 *g*, 30 min, 4 °C), the pellet was resuspended in 20 ml buffer (50 mM Tris-HCl, pH 8.0, 2% (v/v) Triton-X-100) and incubated for 1 h (room temperature, 50 r.p.m.). Following centrifugation (25,000 *g*, 30 min, 4 °C), the supernatant and cell debris were removed from the resulting inclusion body pellet. The inclusion bodies were washed twice by resuspending in 50 mM Tris-HCl (pH 8.0) and incubating for 1 h (room temperature, 50 r.p.m.) before pelleting by centrifugation (25,000 *g*, 30 min, 4 °C). The inclusion bodies were solubilized in 25 mM Tris-HCl and 6 M Gdn-HCl (pH 8.0) for 1 h (60 r.p.m. stirring), and following a final centrifugation (25,000 *g*, 30 min, 4 °C), the supernatant was loaded onto a Superdex 75 HiLoad 26/60 size-exclusion chromatography column (GE Healthcare), equilibrated in 25 mM Tris-HCl (pH 8.0) and 6 M guanidinium-HCl. Protein fractions were collected and concentrated to ~100 μM (Vivaspin concentrators) and flash-frozen for storage at −80 °C. Before folding, proteins were buffer-exchanged into Tris-buffered saline (20 mM Tris-HCl, 100 mM NaCl, pH 8.0) and 8 M urea using 0.5 ml Zeba spin desalting columns with a molecular weight cut-off of 7,000 (Thermo Scientific).

### Protein gel electrophoresis

Samples were mixed in a ratio of 1:3 with loading dye (50 mM Tris-HCl, pH 6.8, 6% (w/v) SDS, 0.3% (w/v) bromophenol blue, 40% (v/v) glycerol), boiled if required (>10 min, 100 °C) and ~14 μl sample loaded onto the gel. Precision Plus Protein Dual Xtra Standards (BioRad) were used as molecular weight markers. We prepared 15% Tris-tricine gels that contained 0.1% (w/v) SDS and 1 M Tris-HCl at pH 8.45 with 13.3% (v/v) glycerol included in the resolving layer. The cathode buffer consisted of 100 mM Tris-HCl, 100 mM tricine and 0.1% (w/v) SDS (pH 8.25) and the anode buffer comprised 200 mM Tris-HCl (pH 8.9). Electrophoresis was conducted with constant currents of 30 mA (stacking) and 60 mA (resolving). Following staining (InstantBlue Coomassie, Abcam), the gels were imaged using a Q9 alliance imaging system (Uvitec) and densitometric analysis was performed using ImageJ. Cold SDS–PAGE makes use of the resistance of natively folded OmpA to denaturation by SDS in the absence of heat, enabling the fraction of folded/unfolded OmpA (the apparent stability) at different urea concentrations to be determined by gel densitometry. The folded fraction was calculated using only the monomer bands as folded/(folded + unfolded). (The inclusion of higher order bands as unfolded species or by normalizing folding against the boiled sample made no appreciable difference to the folded fraction, in contrast to the study reported in ref. ^81^, possibly due to the use of full length OmpA here.) All OmpA-WT liposome conditions were tested at least in duplicate, and all OmpA mutants were measured once.

### Determination of the intrinsic folding rates

The kinetics of intrinsic folding were measured using a QuantaMaster fluorimeter (Photon Technology International (PTI)), including a peltier-controlled temperature unit, controlled by FelixGX software (v4.3). Excitation/emission wavelengths of 280/335 nm were used. OmpA was buffer-exchanged from 25 mM Tris-HCl and 6 M Gdn-HCl (pH 8.0) into 10 mM Tris-HCl and 8 M urea (pH 7.4) using Zeba spin desalting columns (Thermo Scientific). Folding was initiated by rapid dilution of a 3.3 μM unfolded OmpA stock in 8 M urea to a final concentration of 0.2 μM OmpA and 0.48 M urea in the presence of 0.32 mM liposomes (lipid/protein ratio of 1,600:1 (mol/mol)) in 10 mM Tris-HCl and 100 mM NaCl at 30 °C. A minimum of three biological samples were measured for each liposome environment, typically with multiple technical repeats of each preparation, and the kinetics fitted to one-phase exponentials using a custom Python script using SciPy^82^ to derive the observed rate constants, which were then used for further analysis. The kinetics for DMPS-containing liposomes were fitted to a two-phase exponential model based on high residual error in one-phase fits. Kinetic traces showing OmpA folding to an amplitude of ≲75% were not fitted. OmpA folding into PO (palmitoyl-oleoyl) lipid liposomes is less efficient than into the shorter-chain DM lipid analogues (folding yields ~80% and ~30% for DMPC and POPC. respectively).

### Measurement of protein stability by urea titration

Tryptophan fluorescence emission spectra (300–400 nm) with excitation at 280 nm were measured on samples that had been incubated overnight in different concentrations of urea at 30 °C to ensure equilibrium was reached. The fraction of folded protein was then determined from the 335/350 nm fluorescence intensity ratio, corresponding to the emission maxima of folded and unfolded protein, respectively.

### Statistical analysis

For all kinetic data, significant differences were determined by permutation testing^83^, which assumes data exchangeability only under the null hypothesis (that is, it makes no assumption about the underlying distribution of the data). The test statistic was defined as the average difference between a pair of datasets. All permutations of the data in these datasets were randomly sampled (without replacement), and the *P* values determined as the proportion of samples with a test statistic larger than that of the measured data. For the urea stability data, significance was tested using a two-tailed paired *t*-test. Where comparisons are described in the text, relevant significance bars are either shown in figures or included in Supplementary Tables 2–10. All pairwise comparison significance values are presented in Supplementary Table 11 (kinetic data) and Supplementary Table 12 (urea stability data).

### CG-MD simulations

A structural model of full length OmpA was predicted using AlphaFold2, and the structural accuracy of the transmembrane and soluble domains was confirmed by comparison with experimental structures (Protein Data Bank (PDB): 1G90 and 2MQE); for BamA, the crystal structure (PDB: 5D0O) was used. Following any in silico mutations (using Modeller^84^), structures were coarse-grained using the martinize script with an elastic spring network of 1,000 kJ mol^−1^ nm^−^^2^ (upper distance cut-off of 0.7 nm). CG-MD was conducted using GROMACS (v5.0.7) (ref. ^85^) with the Martini (v2) force field^86,87^. Bilayers were built around the transmembrane regions of the protein by randomly placing lipids using the insane script with the protein at the centre of the *x*–*y* plane^88^. CG water molecules were added and then the system neutralized with NaCl and 0.1 M NaCl added. The system was energy-minimized (steepest descent algorithm) and equilibrated with the protein backbone particles position-restrained for 3 ns. The equilibrated system was used to generate production systems for 3 μs (Supplementary Table 1), with a 20 fs time step and frames generated at intervals of 200 ps. The Parinello–Rahman barostat (1 bar)^89^ and velocity rescale thermostat^90^ were applied. A compressibility of 3 × 10^−4^ bar^−1^ was used. The LINCS algorithm constrained bond lengths^91^. Lipid–protein contact analysis used a 0.55 nm distance cut-off to define contacts, performed on merged data from all replicas using gmx mindist. All lipid–protein contacts were normalized to lipid concentrations and simulation time. For lipid density analysis, the trajectories of all simulation replicas were concatenated, the protein orientation was centred and fixed (gmx trjconv), and the densities were calculated using gmx densmap. Residence time was calculated using PyLIPID^69^, with short and long distance cut-offs of 0.475 and 0.8 nm, respectively. The simulations were validated by determining the average area per lipid (using FATSLIM^92^) and surface tension (as in ref. ^93^) over the simulation time course, the *z*-axis average density of the membrane components (gmx density), the protein RMSF (root mean square fluctuation, gmx rmsf) and the convergence of the lipid–protein contacts between repeats (Supplementary Figs. 12–16).

### Laurdan assay

Lipid transition temperatures were measured by laurdan fluorescence using a method adapted from ref. ^68^. Laurdan, dissolved in dimethylsulfoxide, was added to pre-formed liposomes in a lipid/laurdan ratio of 3,200:1 (mol/mol) for a final dimethylsulfoxide concentration of 0.1% (v/v). The liposomes were incubated near their transition temperature overnight. Laurdan fluorescence was excited at 340 nm, and its emission at 440 and 490 nm measured for 10 s using a PTI fluorimeter as described above. Spectra were acquired in steps of either 1 or 0.25 °C at temperatures spanning roughly ±10 °C around the transition temperature, with 3 min equilibration at each temperature. General polarization (GP) was determined from the intensity (*I*) at 440 and 490 nm (averaged over 10 s acquisition) using the following equation:

4 $${\rm{GP}}=(I_{440}-I_{490})/(I_{440}+I_{490})\,$$

Mid-points were determined by numerically taking the first differential of the data. At 30 °C (temperature of the folding and stability assays), all liposomes used in this study were in the fluid phase. They should thus have similar mechanical properties as the Young’s modulus and bending modulus are dominated by the lipid phase^61–63^, although some changes could occur depending on the distribution of the charged lipids^64–66^.

### Bioinformatics

For the experimental structure analysis, 394 OM-annotated proteins from the OPM database^70^, of which 198 have transmembrane regions, were sequence-clustered to 70% sequence identity using CD-HIT^94^ and manually inspected, resulting in 75 structures. Proteins from the OPM database are already aligned in the membrane, and three-dimensional space was split into 1 Å slabs parallel to the membrane plane, with residues assigned on the basis of their Cα position (see Supplementary Fig. 21 for the number of residues per slab). The enrichment/depletion of residues was calculated relative to either the total amino acid content in the protein or in the soluble regions. The 2*σ*/3*σ* significance was calculated separately for enrichment and depletion by finding the standard deviation of all positive and negative enrichments. See Supplementary Table 13 for a list of the proteins used.

For the predicted structure analysis, 2,285 OM-annotated proteins were identified in the European Bioinformatics Institute’s AlphaFold2 database^72^ (accessed December 2021). Signal peptides were predicted (SignalP v5.0 (ref. ^95^) and removed from the structures (proteins with <90% prediction confidence were rejected). The proteins were filtered with pLDDT in AlphaFold2 (>80%), leaving 1,765 proteins. The transmembrane regions and membrane orientation were predicted using the Immers software^70^, and 842 proteins were identified with >0 transmembrane regions (693 proteins with >8 strands, that is, full barrels). Sequences were clustered to 70% sequence identity using CD-HIT^94^, leaving 343 structures, which were processed as for the OPM dataset. See Supplementary Table 13 for a list of the proteins used.

For the sequence data analysis, ~1.3 × 10^6^ sequences in the OMPdb database (accessed August 2021) (ref. ^73^) were quality filtered by topology prediction and pHMM coverage score (both >95%) and sequences missing residues were removed, leaving 71,181 sequences. These were sequence-clustered to 70% sequence identity using CD-HIT^94^, leaving 17,931 sequences. Residue enrichment was carried out as above using residue count away from the centre of the membrane to split the protein into slabs. A distance calibration for residue count was determined from the OPM structures combined with sequence topology prediction (Supplementary Fig. 19). See Supplementary Table 13 for a list of the proteins used.

## Online content

Any methods, additional references, Nature Portfolio reporting summaries, source data, extended data, supplementary information, acknowledgements, peer review information; details of author contributions and competing interests; and statements of data and code availability are available at 10.1038/s41557-023-01319-6.

### Supplementary information

Supplementary Information Supplementary Figs. 1–21, Tables 1–10, legends for Tables 11–13, and unprocessed TLCs and gels for Supplementary Figs. 3, 7, 8 and 11.

Supplementary Table 11 Statistical *P* values for all pairwise difference comparisons of kinetic data. *P* values were determined using permutation testing and the number of permutations above the threshold is indicated (Methods). Data are organized by the figure the data were presented in. Data are provided as an Excel file.

Supplementary Table 12 Statistical *P* values for all pairwise difference comparisons of urea stability data. *P* values were determined using paired two-tail *t*-test for all (OmpA mutant, lipid environment) pairs. Data are provided as an Excel file.

Supplementary Table 13 Datasets used for bioinformatics analysis. PDB accession codes for all OPM structures and uniport accession codes for all AlphaFold2 structures and OMP sequences considered in the bioinformatic analysis. Datasets are shown before and after sequence clustering (70% identity). Data are provided as an Excel file.

### Source data

Source Data Fig. 1 Numerical source data for Fig. 1.

Source Data Fig. 1 Unprocessed TLC for Fig. 1e.

Source Data Fig. 2 Numerical source data for Fig. 2.

Source Data Fig. 3 Numerical source data for Fig. 3.

Source Data Fig. 4 Numerical source data for Fig. 4.

Source Data Fig. 5 Numerical source data for Fig. 5.

Source Data Fig. 6 Numerical source data for Fig. 6.

Source Data Extended Data Fig. 2 Numerical source data for Extended Data Fig. 2.

Source Data Extended Data Fig. 3 Numerical source data for Extended Data Fig. 3.

Source Data Extended Data Fig. 4 Numerical source data for Extended Data Fig. 4.

Source Data Extended Data Fig. 4 Unprocessed TLC for Extended Data Fig. 4b.

Source Data Extended Data Fig. 5 Numerical source data for Extended Data Fig. 5.

Source Data Extended Data Fig. 5 Unprocessed TLC for Extended Data Fig. 5b,f.

Source Data Extended Data Fig. 6 Numerical source data for Extended Data Fig. 6.

Source Data Extended Data Fig. 6 Unprocessed TLC for Extended Data Fig. 6e.

Source Data Extended Data Fig. 7 Numerical source data for Extended Data Fig. 7.

Source Data Extended Data Fig. 8 Numerical source data for Extended Data Fig. 8.

Source Data Extended Data Fig. 9 Numerical source data for Extended Data Fig. 9.

Source Data Extended Data Fig. 10 Numerical source data for Extended Data Fig. 10.

## Data Availability

Source data are provided with this paper. The Source Data comprise all folding kinetics, EM images, gels/TLCs, DLS and ζ-potential data, thinned MD trajectories and fluorescence curves, including for all Supplementary Figures. They are also freely available at the University of Leeds Data Repository (10.5518/1168).

## Code availability

Code for the ξ-prediction model can be accessed at https://github.com/JonMarks29/zeta-potential-prediction.
