# Supplementary material for: Protein–lipid charge interactions control the folding of outer membrane proteins into asymmetric membranes
Source: Nat Chem. 2023 Sep 14;15(12):1754–64. doi: 10.1038/s41557-023-01319-6 (PMC10695831; doi:10.1038/s41557-023-01319-6)
Supplement: Supplementary file 1 — Supplementary Figs. 1–21, Tables 1–10, legends for Tables 11–13, and unprocessed TLCs and gels for Supplementary Figs. 3, 7, 8 and 11. [file 41557_2023_1319_MOESM1_ESM.pdf]

nature chemistry

Article https://doi.org/10.1038/s41557-023-01319-6

Protein–lipid charge interactions control the folding of outer membrane proteins into asymmetric membranes

In the format provided by the authors and unedited

Contents

Supplementary Figures 1-21 2

Supplementary Tables 1-13 29

Unprocessed TLCs and gels for supplementary figures 40

Supplementary figure 3 40

Supplementary figure 7 41

Supplementary figure 8 42

Supplementary figure 11 46

Supplementary Figures 1-21

A

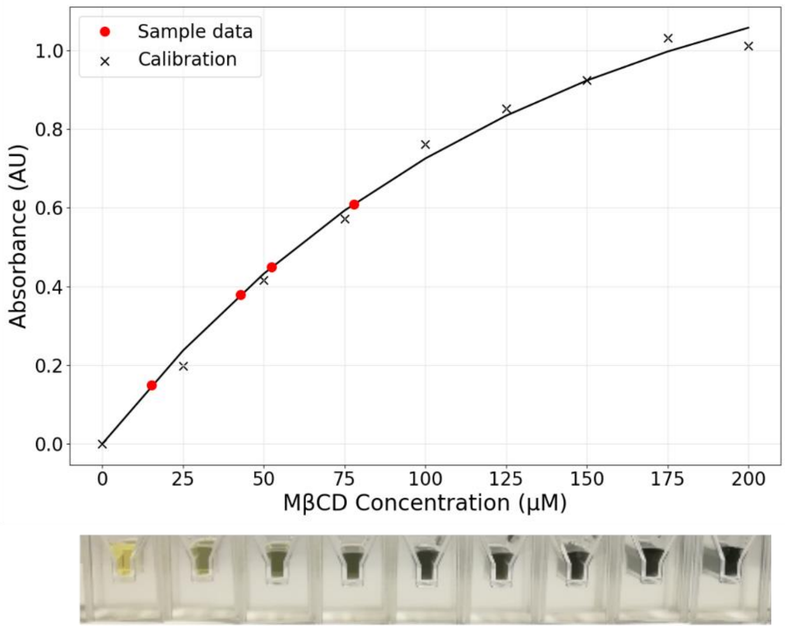

B

Sample	Initial concentration (μM)	Final Concentration (μM)
1	67 000	21
2	67 000	44
3	67 000	52
4	67 000	77

Supplementary Figure 1: MβCD is effectively removed by ultracentrifugation. (A) The sugar anthrone assay can colorimetrically detect MβCD at µM concentrations (black crosses: calibration, red circles: sample data). Calibrant samples are shown below**. (B)** Example measurements of MβCD concentrations before and after ultracentrifugation (red circles in panel (A)). Typical reductions are >1000x, reducing free MβCD, and hence its associated lipid, to insignificant quantities for the assays described. Four replicates with an identical initial concentration of 67,000 µM are shown.

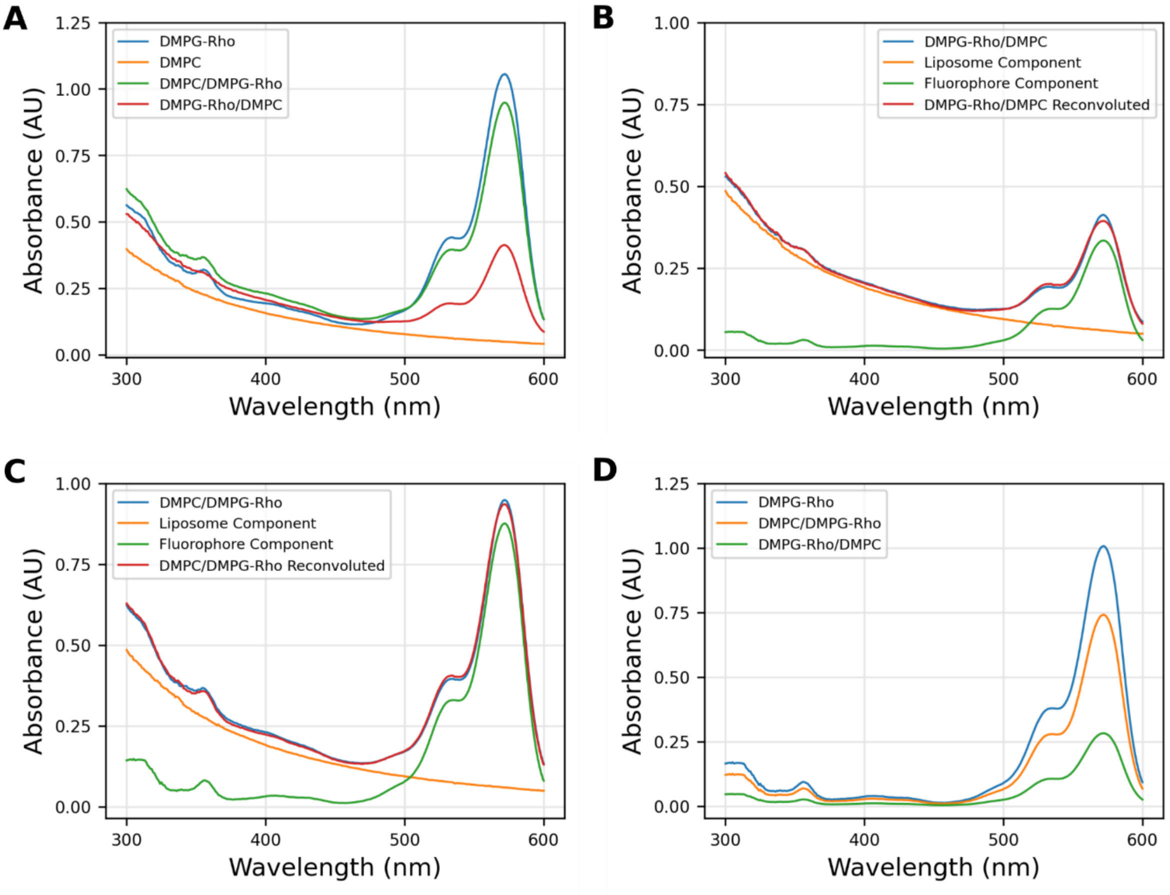

Supplementary Figure 2: DMPC and DMPG lipids are competent to exchange through MβCD-mediated exchange. (A) Raw absorbance spectra of unexchanged DMPG-Rho liposomes (DMPG + 1% (mol/mol) DPPE-rhodamine) and DMPC liposomes, and exchanged liposomes, as indicated. **(B)** Deconvoluted absorbance spectra for DMPG-Rho/DMPC exchange and **(C)** DMPC**/**DMPG-Rho exchange, separating the liposome and fluorophore absorbance components. **(D)** Liposome concentration normalised fluorophore components of DMPG-Rho and the exchanged samples, showing loss of fluorescence from the DMPC/DMPG-Rho and gain of fluorescence in the DMPG-Rho/DMPC samples, indicating successful lipid exchange.

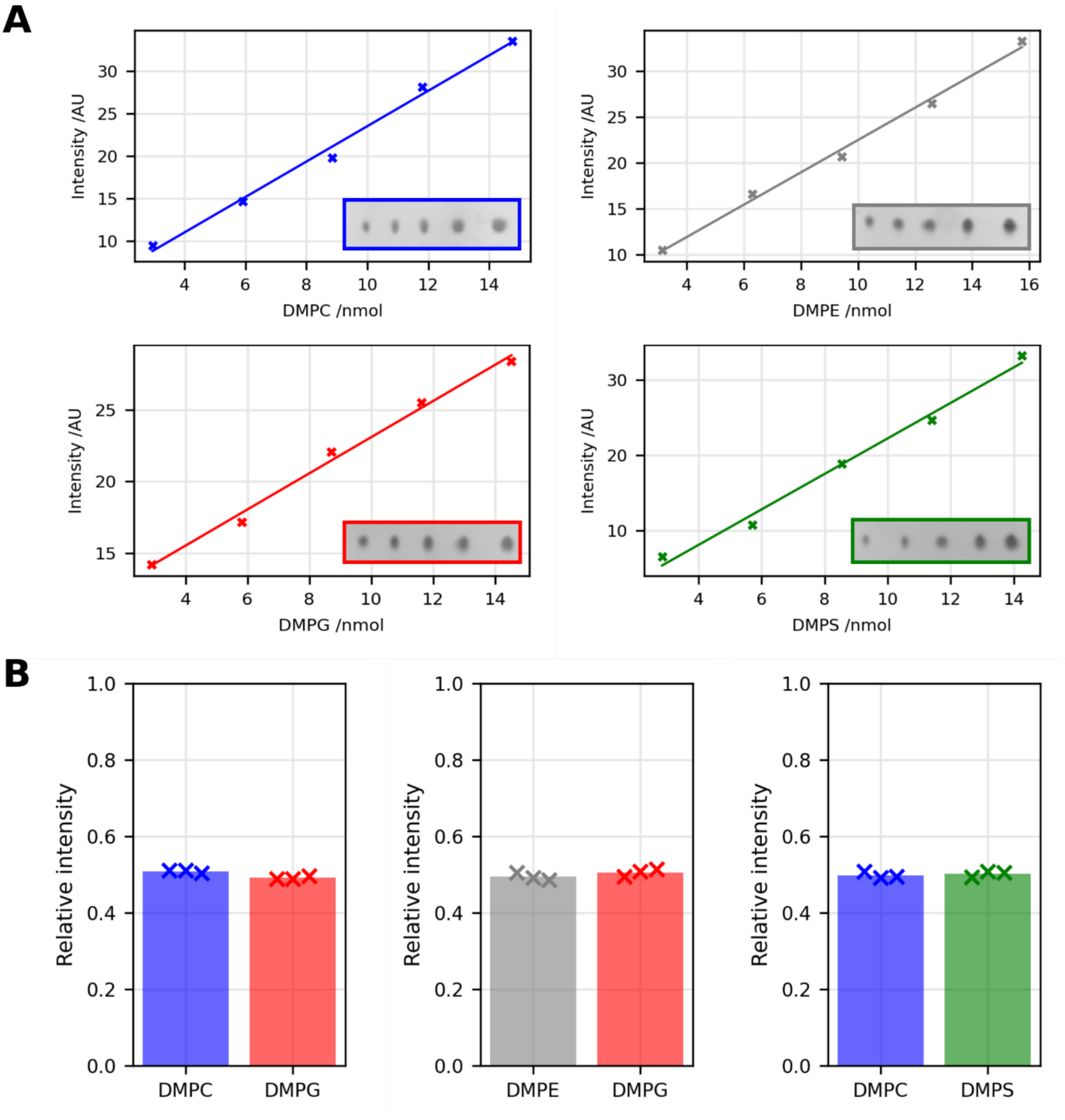

Supplementary Figure 3: Thin layer chromatography (TLC) can be used to determine relative lipid ratios. (A) DMPC (blue), DMPG (red), DMPE (grey) and DMPS (green) lipid staining depth is directly and linearly proportional to amount of lipid loaded. One example from three replicates shown. An example TLC plate is shown inset in each. **(B)** DMPC-DMPG, DMPE-DMPG and DMPC-DMPS lipids stain to equivalent depths for the same molar lipid amounts (10 nmol of each lipid loaded).

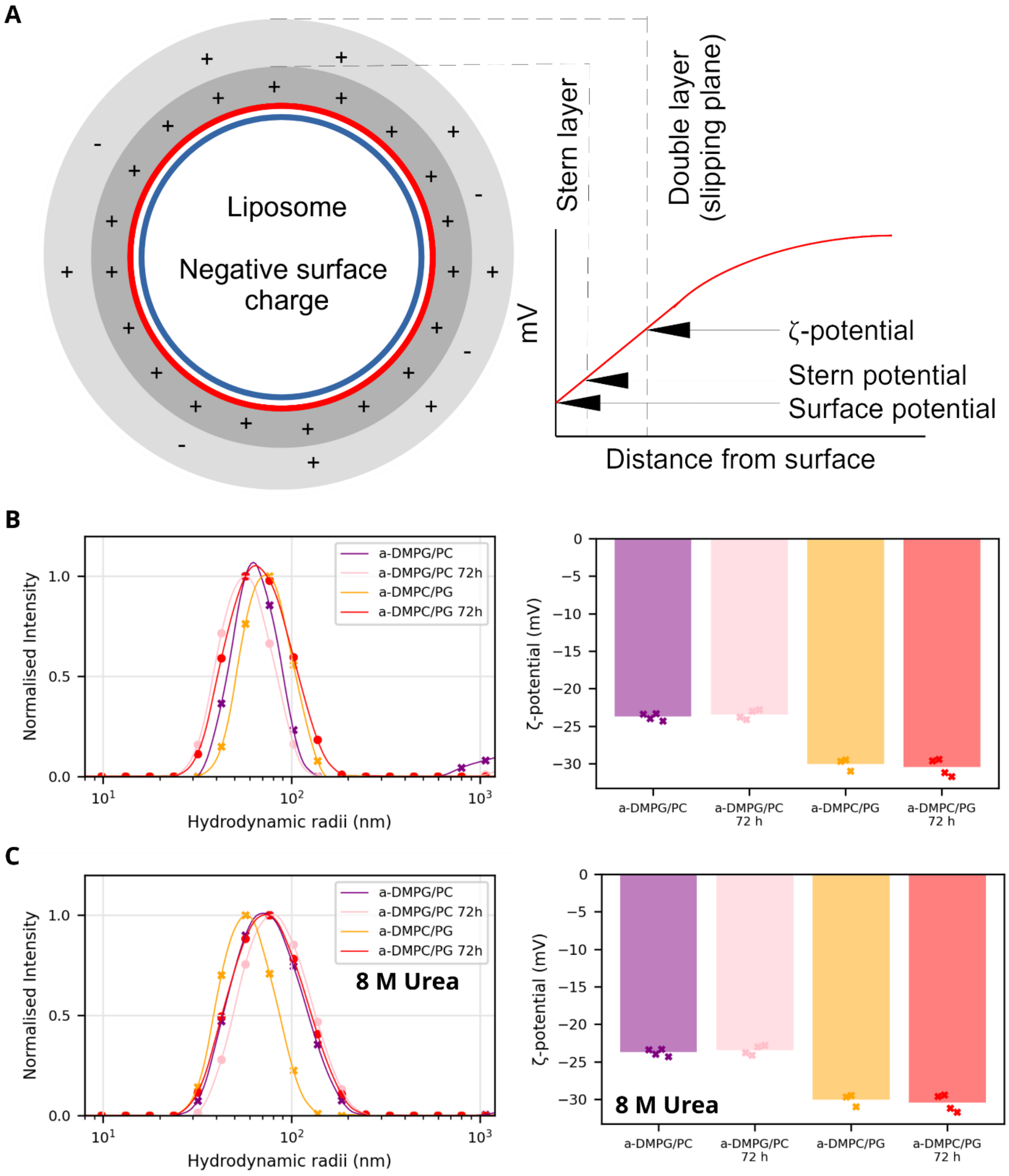

Supplementary Figure 4: Liposome ζ-potential can be used to measure outer leaflet lipid composition. (A) Physical basis of the ζ-potential as the charge on the liposome slipping plane through solution (double layer). Liposome hydrodynamic radius and ζ-potential, and hence asymmetry, is stable over at least 72 hours in the absence **(B)** or presence **(C)** of 8 M urea. Note that asymmetry was determined without urea present.

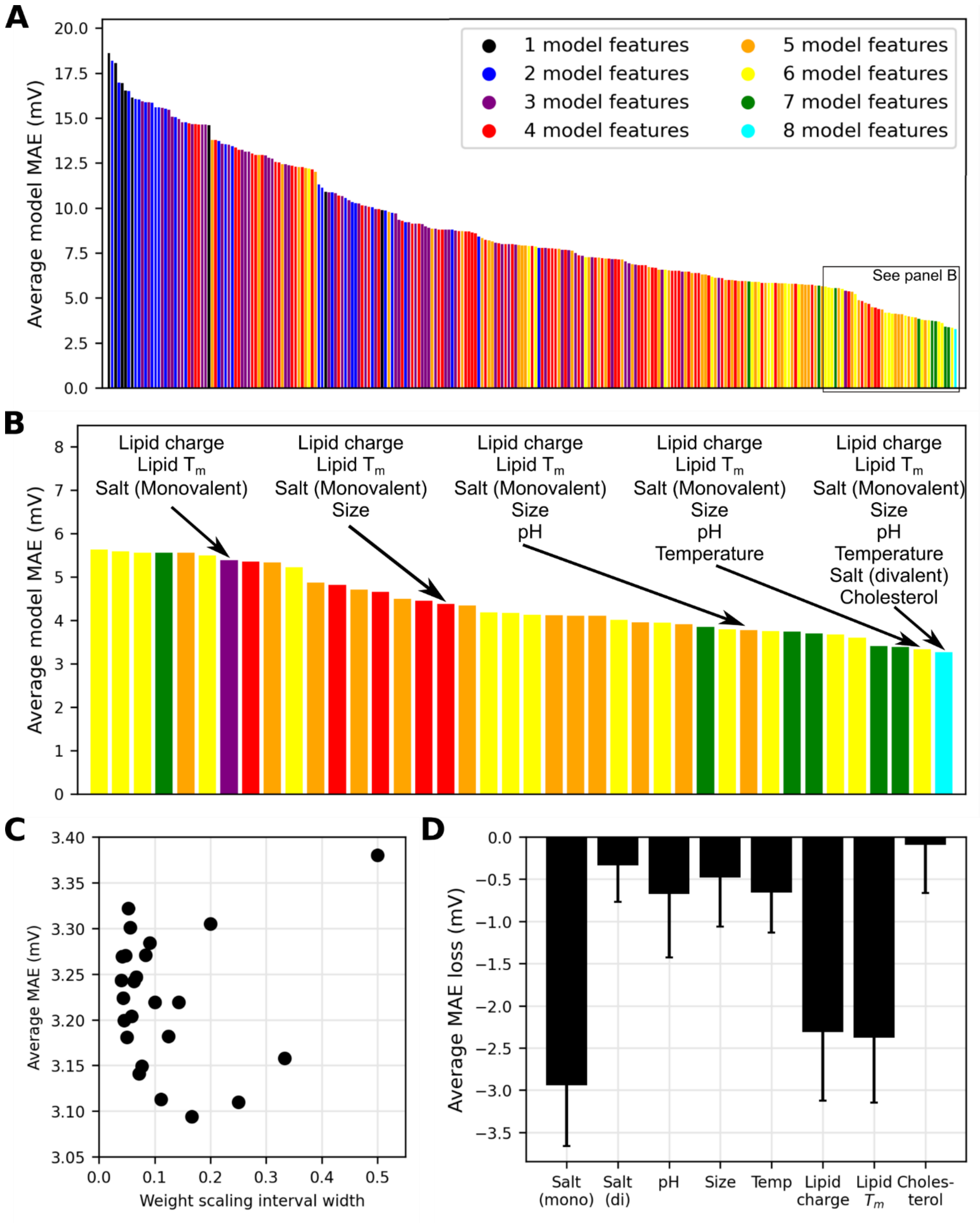
A
Average model MAE (mV)
1 model features
2 model features
3 model features
4 model features
5 model features
6 model features
7 model features
8 model features
See panel B
B
Average model MAE (mV)
Lipid charge
Lipid Tm
Salt (Monovalent)
Size
pH
Temperature
Salt (divalent)
Cholesterol
C
Average MAE (mV)
Weight scaling interval width
D
Average MAE loss (mV)
Salt (mono)
Salt (di)
pH
Size
Temp
Lipid charge
Lipid Tm
Choles-terol

Supplementary Figure 5: Understanding the ζ-potential prediction model. (A) The average MAE of models generated using all combinations of the eight dataset features (monovalent/divalent salt concentrations, pH, liposome size, temperature, cholesterol fraction and overall lipid charge/T_m). **(B)** Expanded boxed region in (A). Considering the best model generated with reduced feature count indicates relative feature importance. **(C)** Effect of weighting the ζ-potential target values by their associated measurement error. Scaling intervals are centred on 0.5. An interval of 0.18 yields the best model improvement – i.e. all target values are weighted between 0.41 and 0.59 according to their error. **(D)** Average MAE loss from single parameter ablation from the final model. All averages MAEs from 50 trained models, error bars are standard deviation.

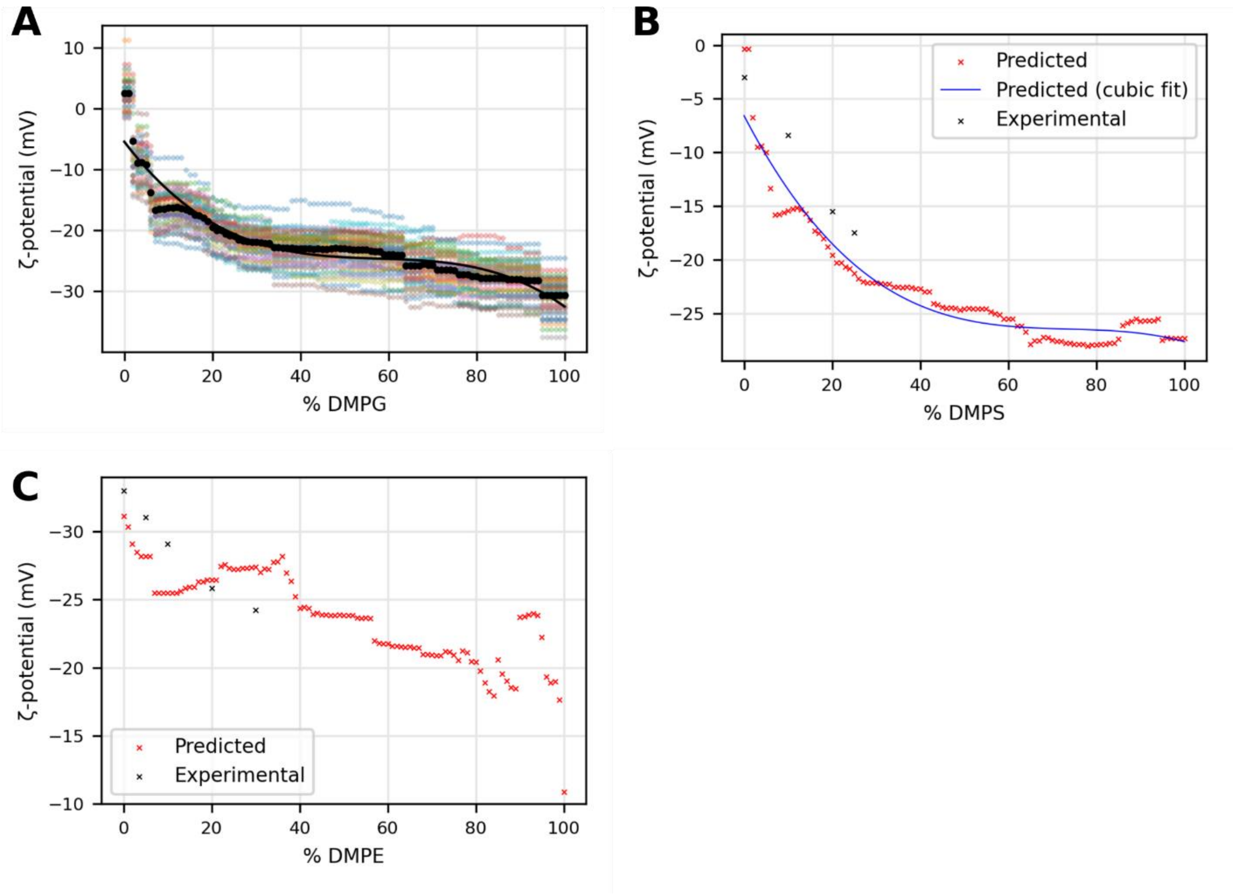

Supplementary Figure 6: ζ-potential prediction model. (A) Individual predictions (coloured crosses) and their average (black circles) from the model ensemble for prediction in Figure 1H (DMPC-PG). The fitted cubic is also shown. **(B)** Average prediction for DMPS-PC and **(C)** DMPE-PG lipid mixes compared with the experimental data. Note that high-DMPE content LUVs (> 30-40% mol/mol) cannot be synthesised due to the strong negative curvature of DMPE.

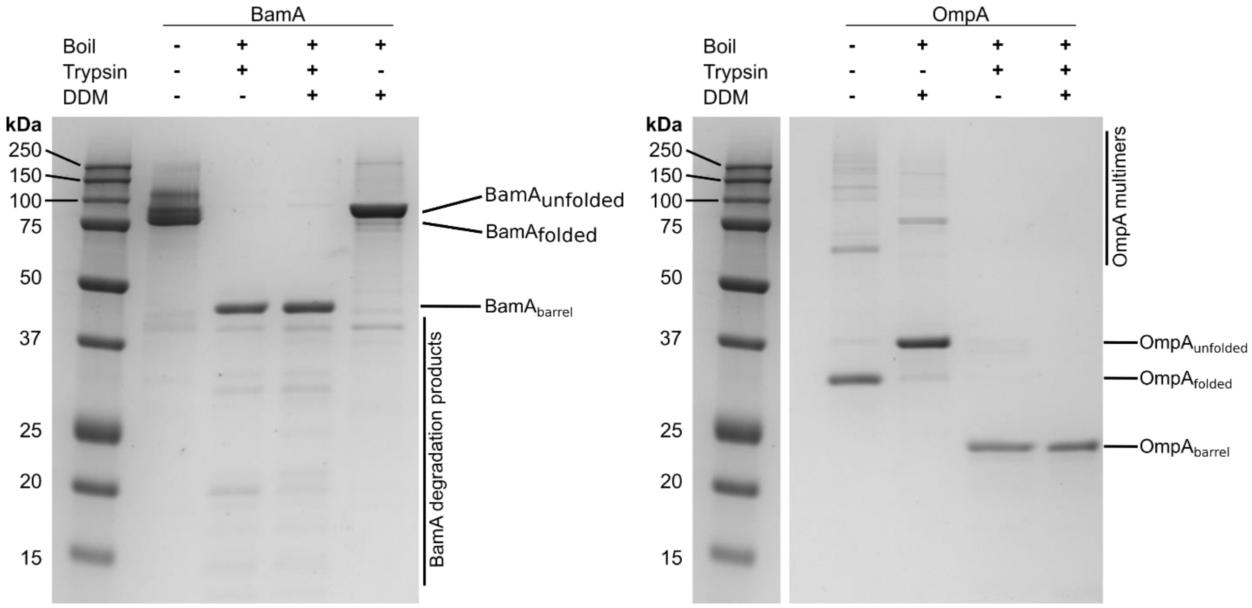

Supplementary Figure 7: OmpA and BamA fold unidirectionally. OmpA and BamA were folded into DMPC liposomes and each were then incubated with trypsin (1000:1 molar ratio substrate:trypsin) overnight and compared to DDM-solubilised and trypsin cleaved samples treated identically. OmpA and BamA each show complete cleavage of their periplasmic domains in DMPC liposomes, indicating that they have folded unidirectionally into the bilayer with their water-soluble domains exposed to the bulk solvent.

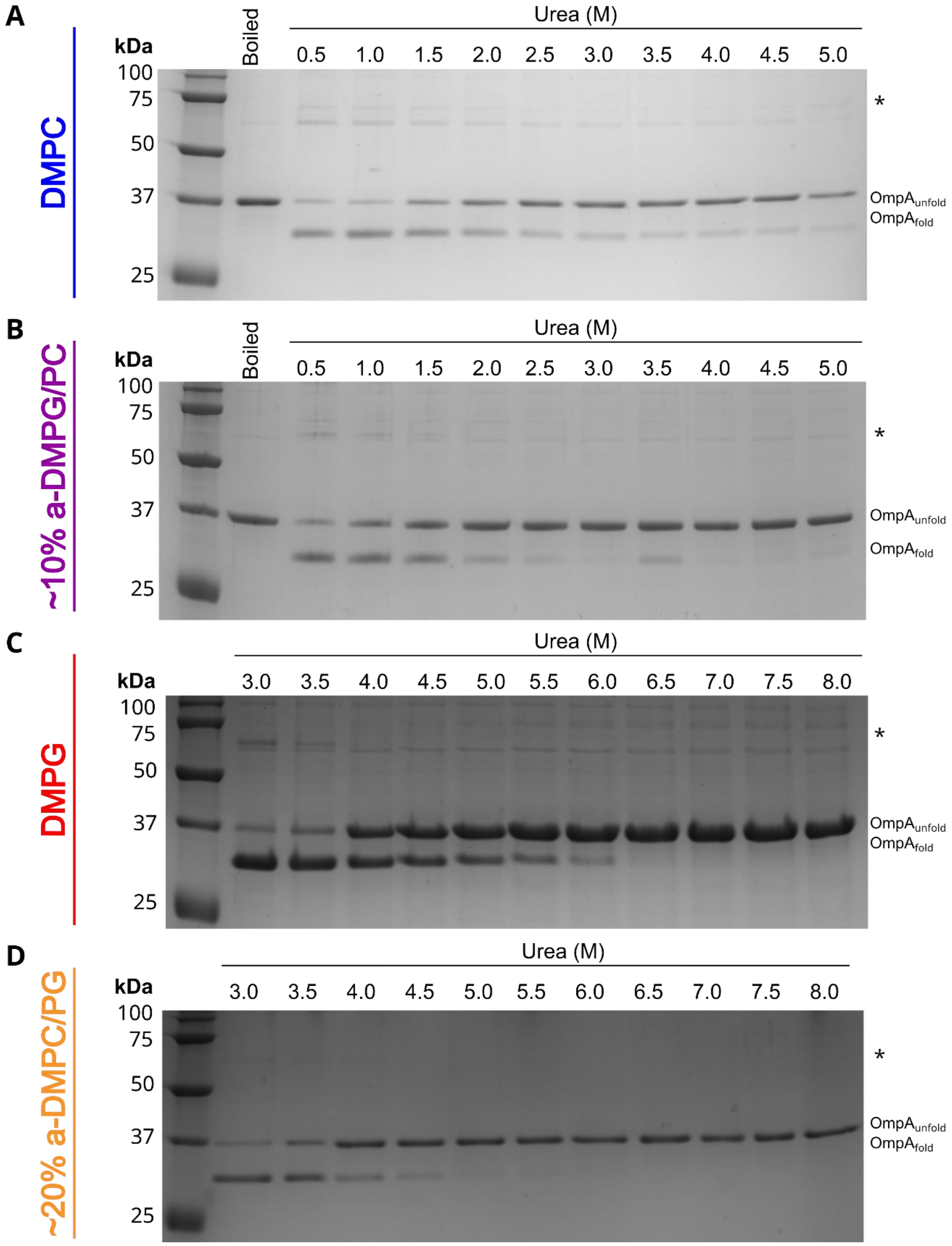

Supplementary Figure 8: Example OmpA folding yields over 0.5 – 5 M urea in different liposome compositions. OmpA (initially unfolded in 8 M urea) was incubated with the relevant liposomes at each final concentration of urea indicated overnight at 30 °C. The proportion of folded protein was then analysed by cold SDS-PAGE (Methods) and the fraction folded determined from the folded and unfolded monomer bands only. Inclusion of higher order bands (indicated by *) in the densitometry analysis, or normalising against the boiled sample, showed minimal difference to final fraction folded (Methods).

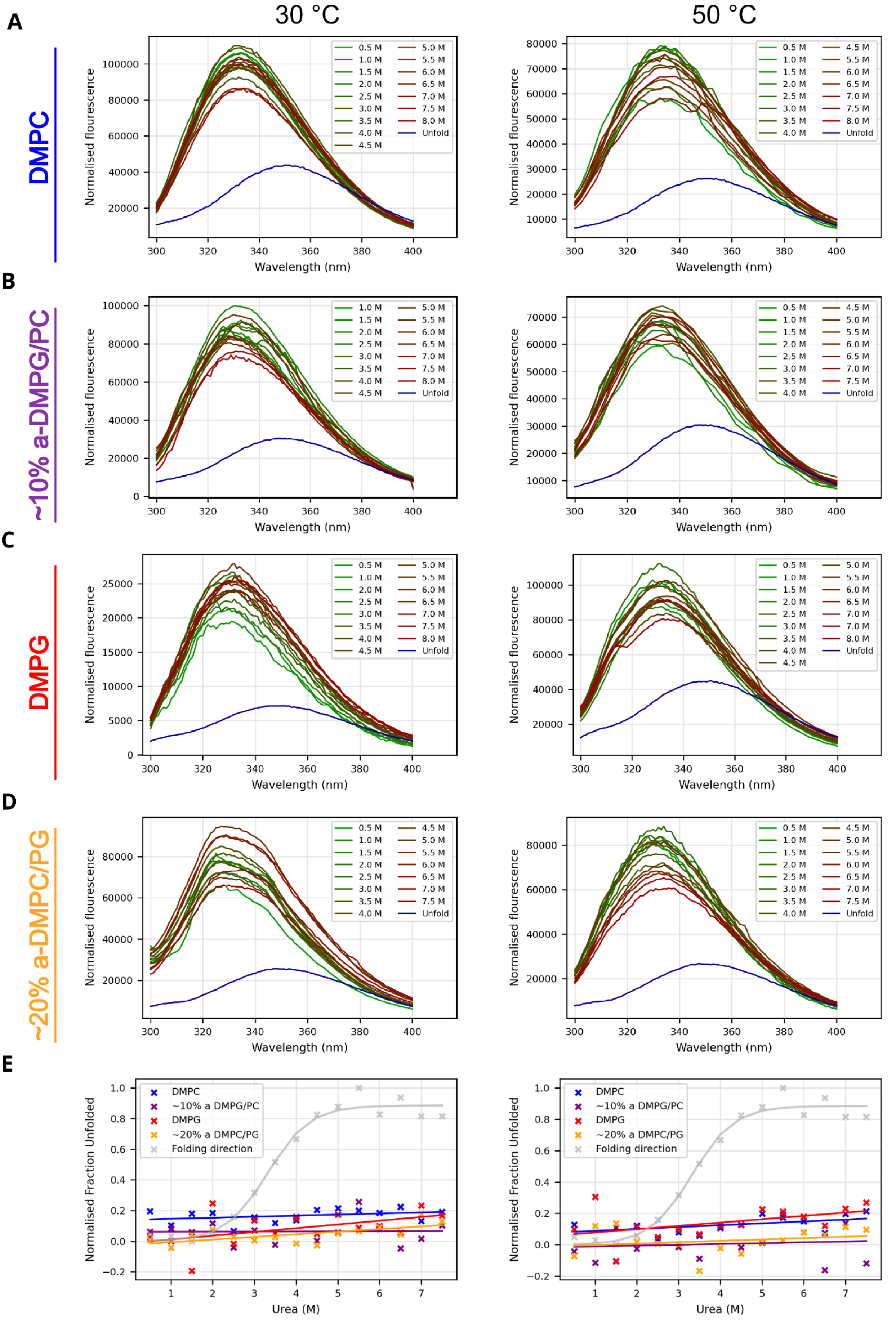

A
30 °C
50 °C
DMPC
B
~10% a-DMPG/PC
C
DMPG
D
~20% a-DMPC/PG
E
Normalised flourescence
Wavelength (nm)
0.5 M
1.0 M
1.5 M
2.0 M
2.5 M
3.0 M
3.5 M
4.0 M
4.5 M
5.0 M
5.5 M
6.0 M
6.5 M
7.0 M
7.5 M
8.0 M
Unfold
Normalised Fraction Unfolded
Urea (M)
DMPC
~10% a DMPG/PC
DMPG
~20% a DMPC/PG
Folding direction

Supplementary Figure 9: Urea-unfolding curves of OmpA measured by tryptophan fluorescence. (A-D) Native OmpA folded into LUVs of different lipid composition and organisation does not unfold following incubation at 30 °C (left) or 50 °C (right) in different concentrations of urea overnight. The slight reduction in intensity indicates that the liposomes have started to aggregate during the overnight reaction. The spectrum of unfolded OmpA in 7.5 M urea in the absence of lipid is shown for comparison. **(E)** The intensity ratio (335/350 nm) for each condition shows no evidence for unfolding of natively folded and membrane embedded OmpA at all urea conditions measured. By contrast, OmpA folds into 10:90 DMPC:DMPG LUVs with a midpoint of ~ 3.5M urea (grey) (folding was measured at 30 °C, both panels).

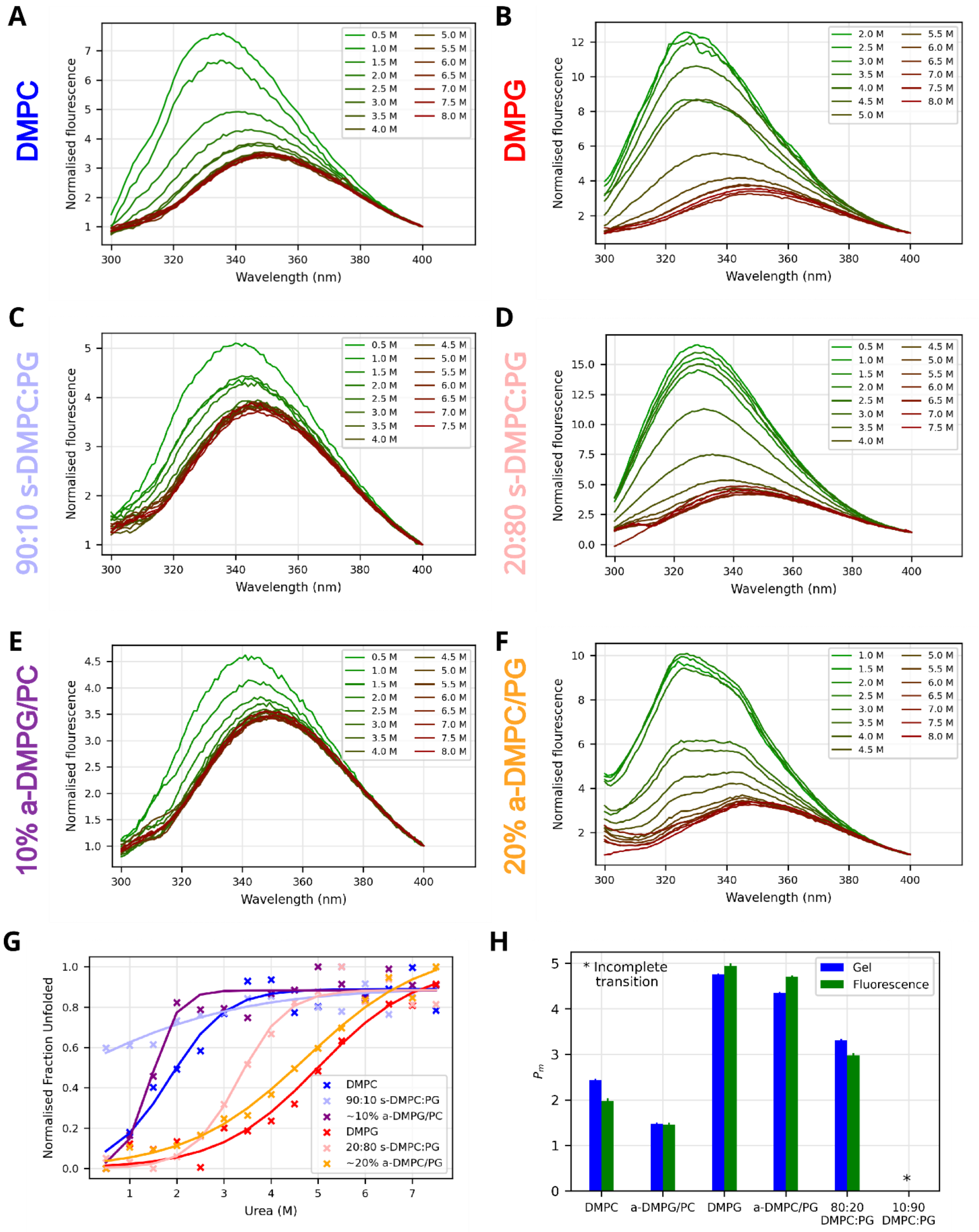

Supplementary Figure 10: Urea-folding curves for OmpA in different liposome conditions measured by tryptophan fluorescence agree well with cold SDS-PAGE data. **(A-F)** Tryptophan fluorescence emission spectra of OmpA folded into LUVS of different liposome composition in 0.5 – 8 M urea. **(G)** Extracting the 335/350 nm ratio of each spectrum and normalising to each liposome condition shows urea transition curves that **(H)** agree well with the midpoints of folding (P_m) determined using cold SDS-PAGE (the differences are < 0.5 M urea, the increment size). Error bars indicate +/- goodness of fit (average difference between observed and fitted data) (n = 2 (gel) or n = 1 (fluorescence)).

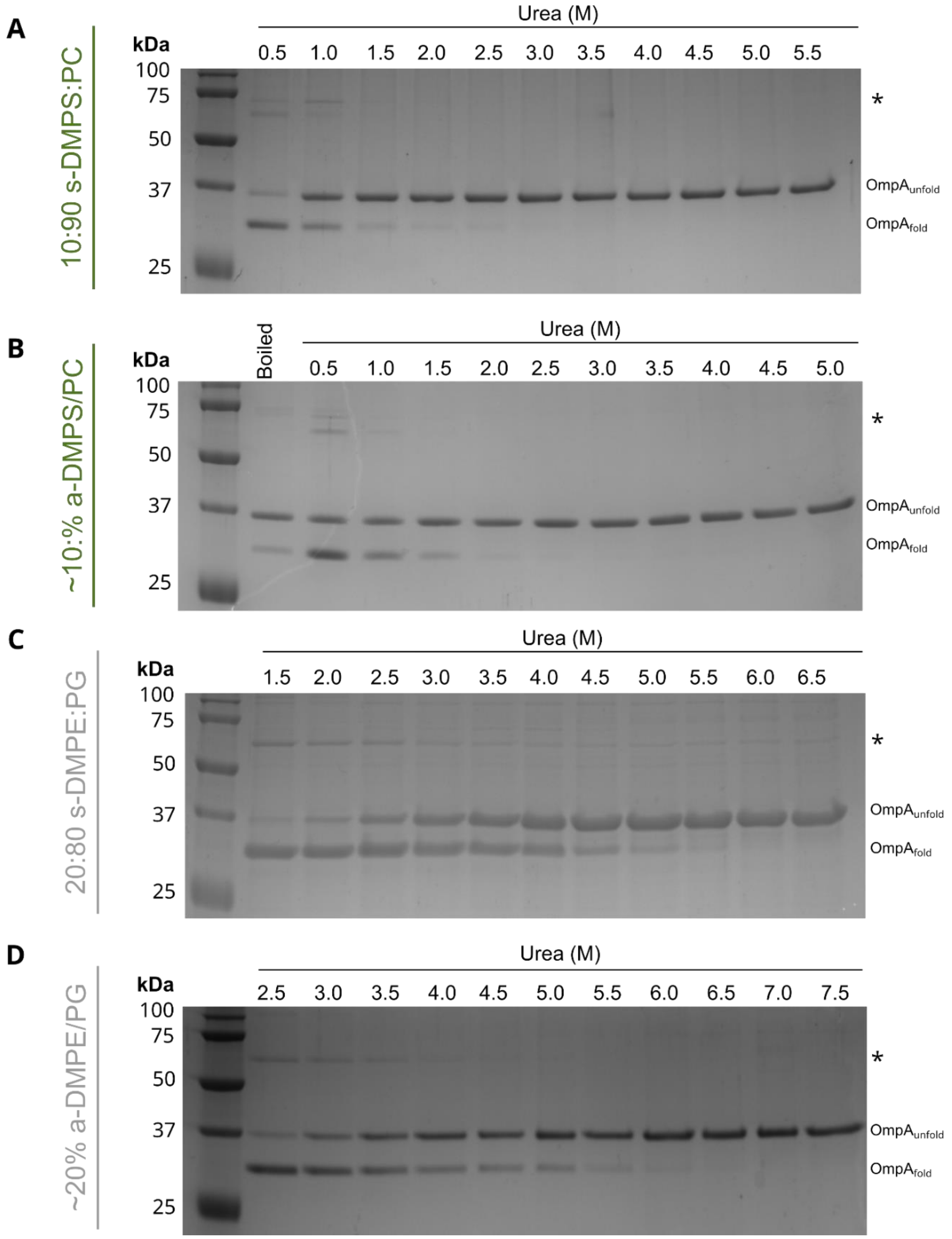

Supplementary Figure 11: Example cold SDS PAGE gels of the folding yield OmpA into DMPE and DMPS-containing liposomes at different concentrations of urea. (A,B) Example gels for OmpA folding into DMPS-DMPC symmetric and asymmetric liposomes at urea concentrations 0.5-5.5 M. **(C,D)** Example gels for OmpA folding into DMPE-DMPG symmetric and asymmetric liposomes at different urea concentrations. Note higher bands (*) were not used to calculate midpoints, see Methods.

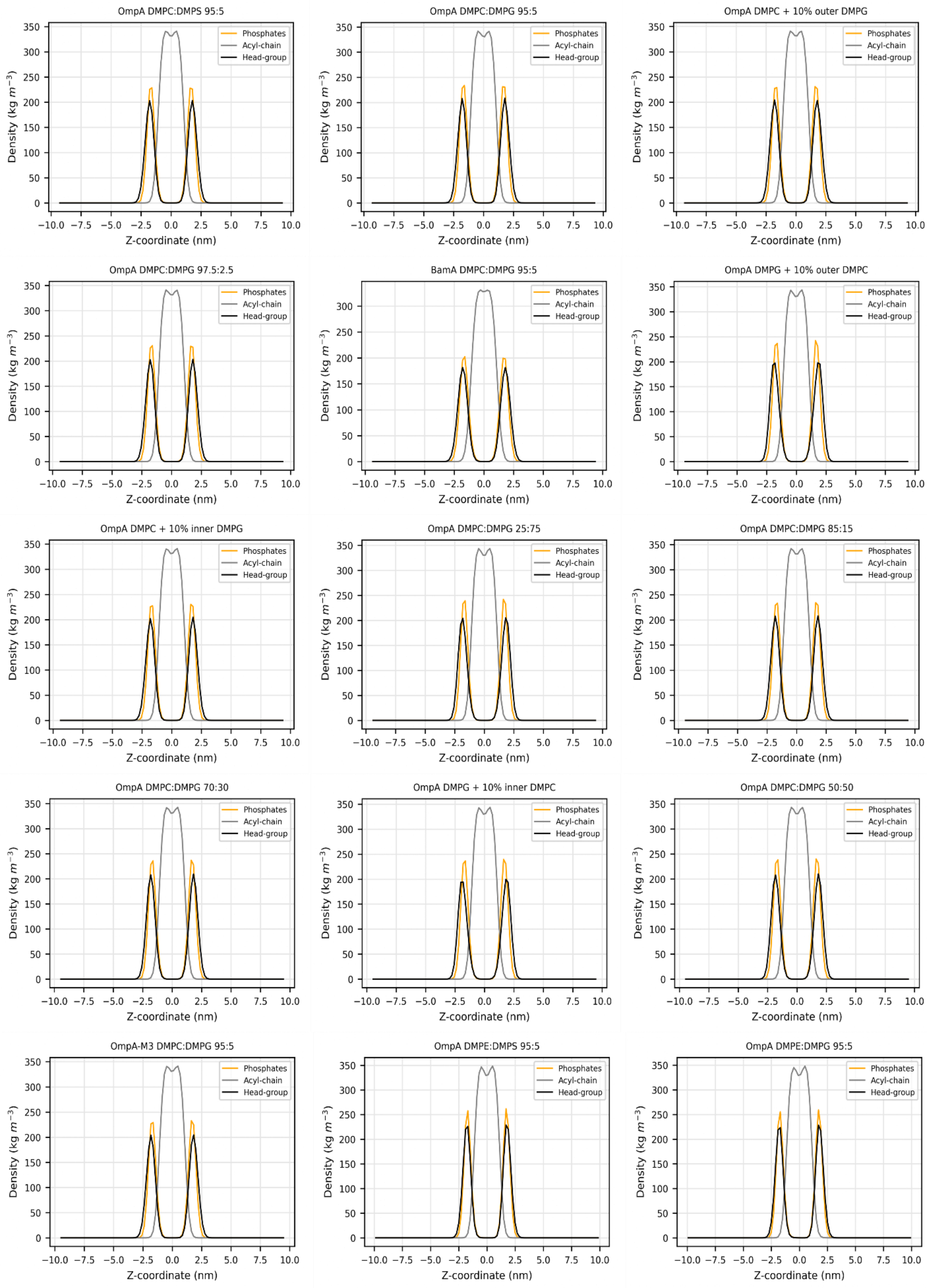

Supplementary Figure 12: Average transverse membrane density profiles for major lipid components shown for the membrane of each simulation. Densities calculated over the whole trajectories; acyl-chain density has been scaled by 1/3 to allow for comparison.

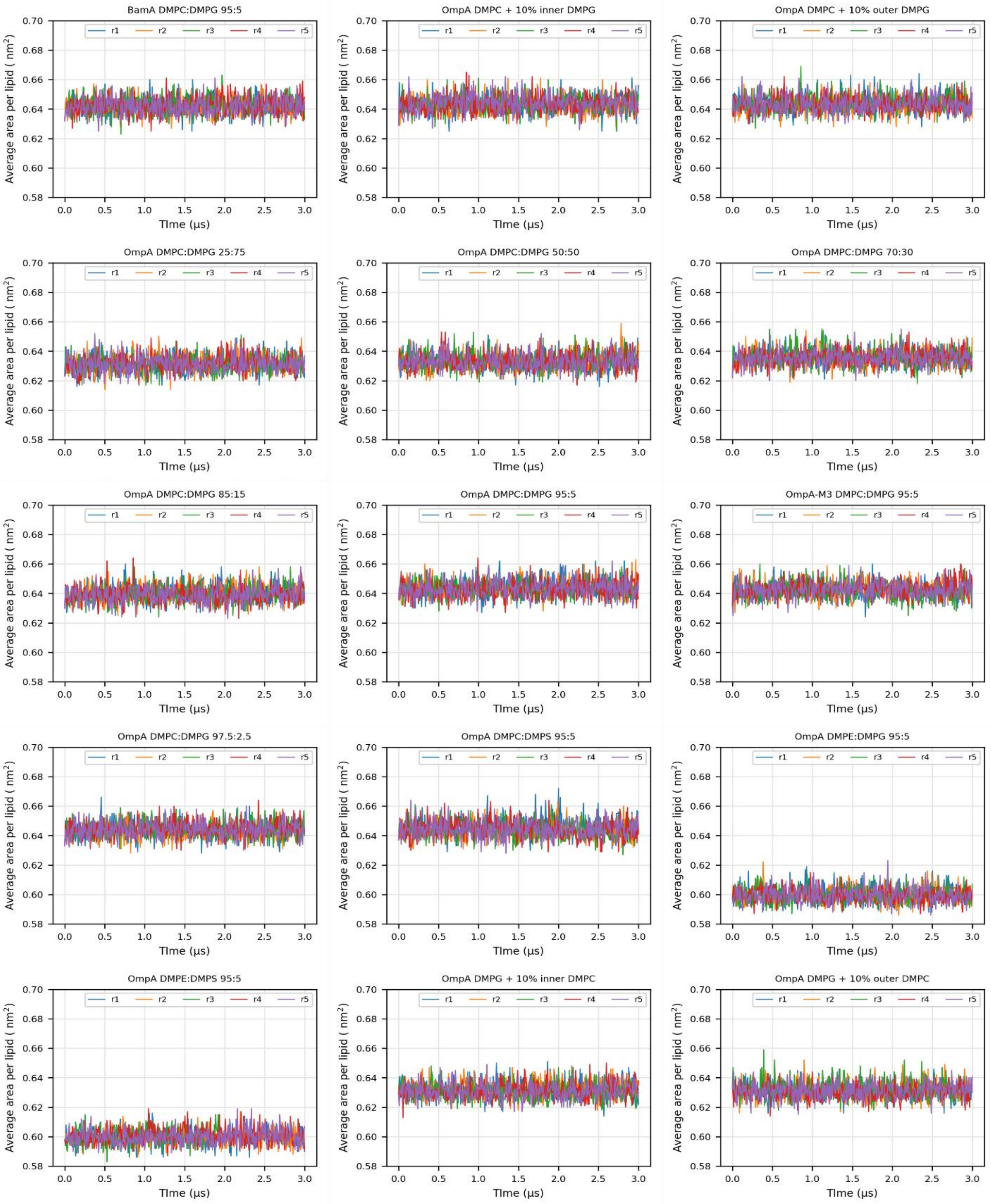

Supplementary Figure 13: Average area per-lipid for the membrane of each simulation membrane and replicate, shown for the 3 μs trajectory. Note that the coarse-grained representation of DMPE has a smaller area than DMPC/PG/PS.

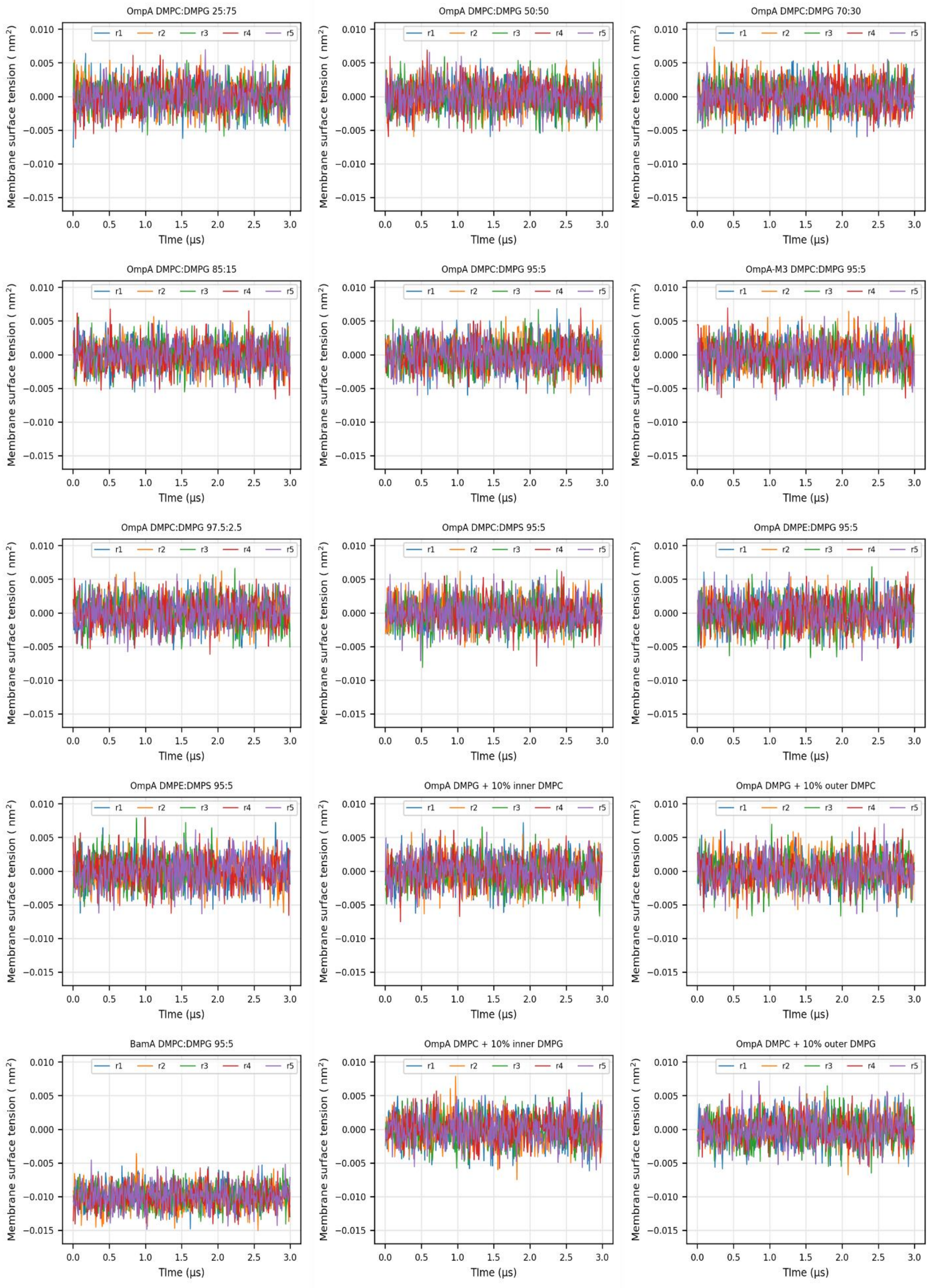

Supplementary Figure 14: Average membrane surface tension shown over the 3 μs simulation time for each lipid membrane and replicate. Note that BamA induces a slight, stable negative tension into the membrane.

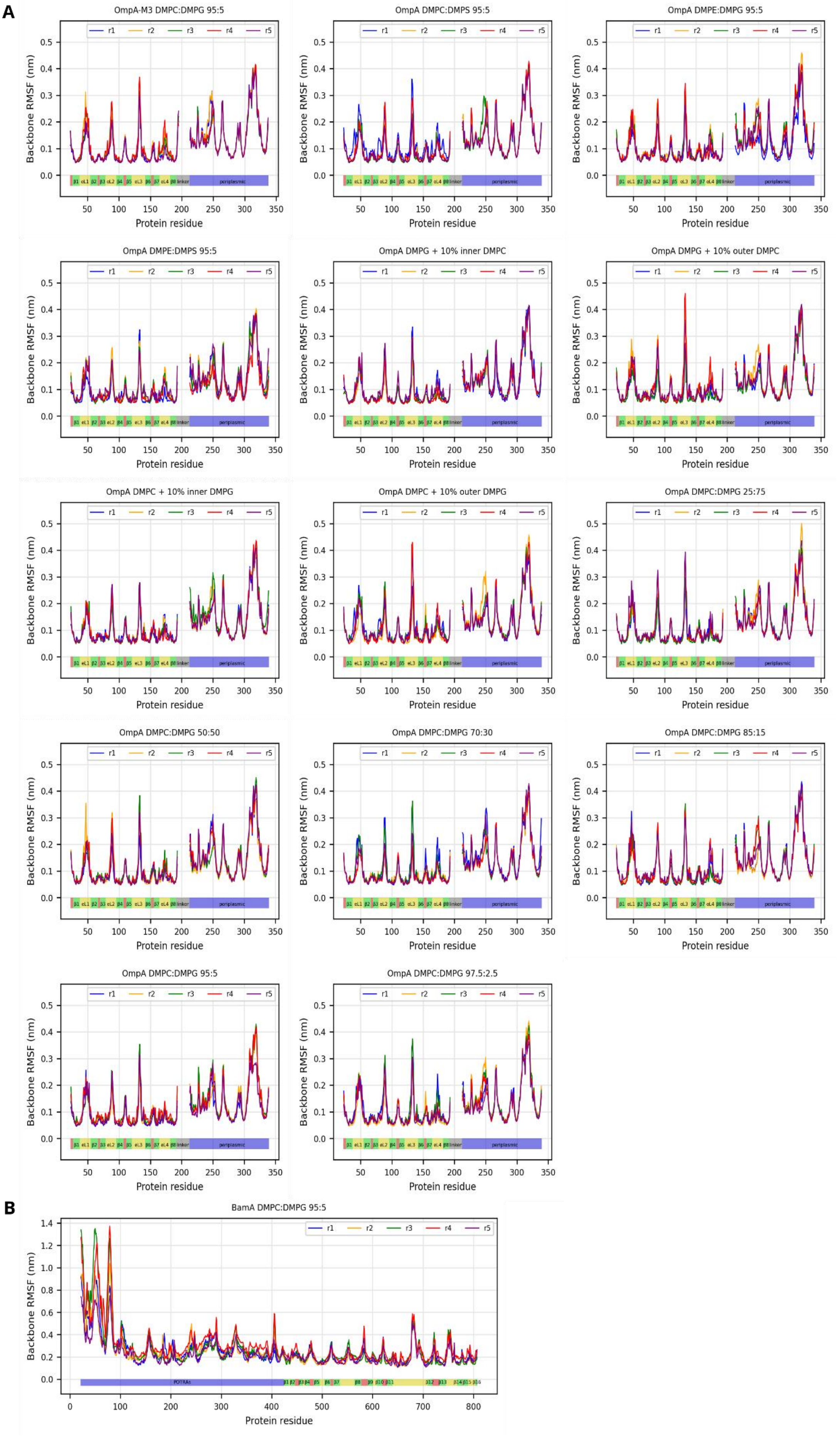
A
OmpA-M3 DMPC:DMPG 95:5
OmpA DMPC:DMPS 95:5
OmpA DMPE:DMPG 95:5
OmpA DMPE:DMPS 95:5
OmpA DMPG + 10% inner DMPC
OmpA DMPG + 10% outer DMPC
OmpA DMPC + 10% inner DMPG
OmpA DMPC + 10% outer DMPG
OmpA DMPC:DMPG 25:75
OmpA DMPC:DMPG 50:50
OmpA DMPC:DMPG 70:30
OmpA DMPC:DMPG 85:15
OmpA DMPC:DMPG 95:5
OmpA DMPC:DMPG 97.5:2.5
r1
r2
r3
r4
r5
Backbone RMSF (nm)
Protein residue
periplasmic
B
BamA DMPC:DMPG 95:5
POTRAs
Backbone RMSF (nm)
Protein residue

Supplementary Figure 15: Protein RMSF for (A) OmpA and (B) BamA in each lipid membrane. The flexible linker between the transmembrane and soluble C-terminal domains of OmpA allow these two domains to move independently and thus they are considered separately.

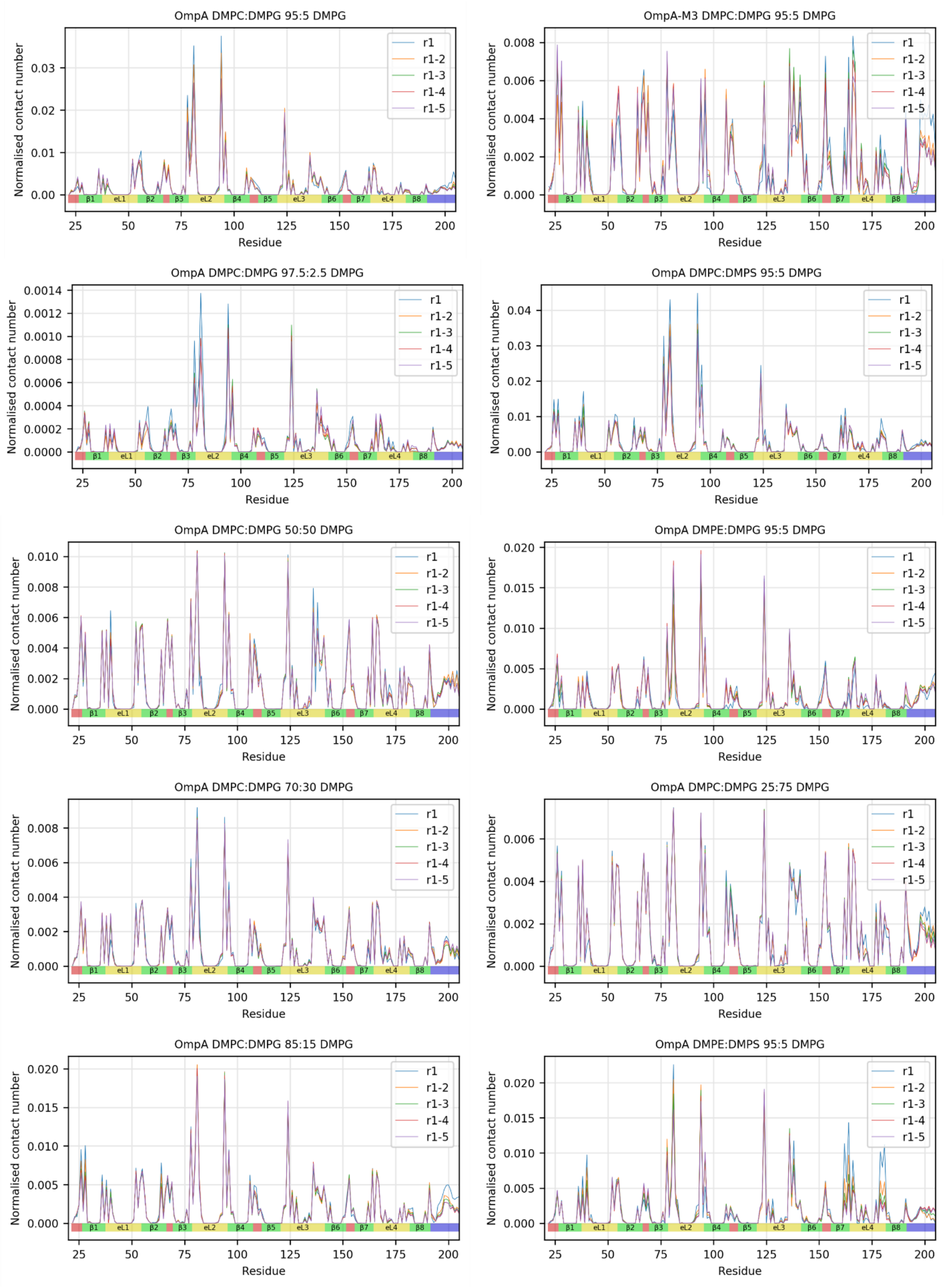

Supplementary Figure 16: Lipid-protein contacts have converged. The normalised lipid-protein interactions (number of interactions between each type of lipid and each protein residue normalised by lipid concentration and simulation frame number) are the same for individual replicates alone or combined, shown here for OmpA in symmetric membranes.

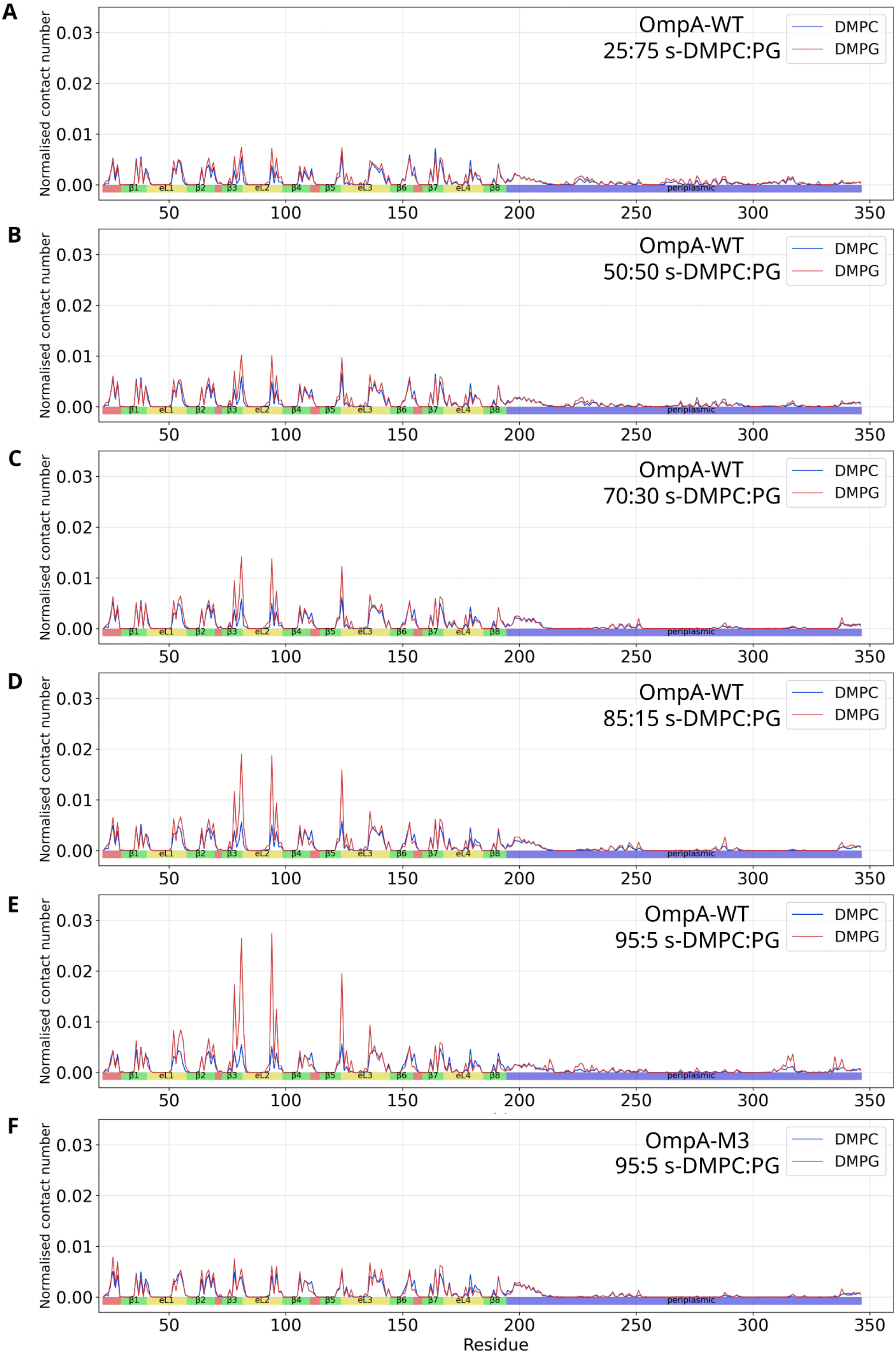

A
OmpA-WT
25:75 s-DMPC:PG
B
OmpA-WT
50:50 s-DMPC:PG
C
OmpA-WT
70:30 s-DMPC:PG
D
OmpA-WT
85:15 s-DMPC:PG
E
OmpA-WT
95:5 s-DMPC:PG
F
OmpA-M3
95:5 s-DMPC:PG
DMPC
DMPG
Normalised contact number
0.00
0.01
0.02
0.03
β1
eL1
β2
β3
eL2
β4
β5
eL3
β6
β7
eL4
β8
periplasmic
50
100
150
200
250
300
350
Residue

Supplementary Figure 17: OmpA specifically interacts with DMPG in a titratable manner. (A-E) Normalised lipid-protein contacts (number of interactions between each type of lipid and each protein residue normalised by lipid concentration and simulation frame number) between OmpA (WT) and DMPC or DMPG lipids with a total DMPG fraction of 75%, 50%, 30%, 15% or 5% in symmetric membranes (as indicated on panels). The sequence of OmpA is shown below (strands (green), extracellular loops (yellow) intracellular turns (red), and the C-terminal water soluble domain (blue)). Peaks are more prominent with less DMPG due to a reduction of background signal. **(F)** Normalised lipid-protein contacts between OmpA-M3 (R81S, K94S, R124S) and DMPC or DMPG lipids with total DMPG fraction of 5%. Compared with WT OmpA (panel E), it is clear that the specific interactions with R81, K94 and R124 are abrogated in the variant.

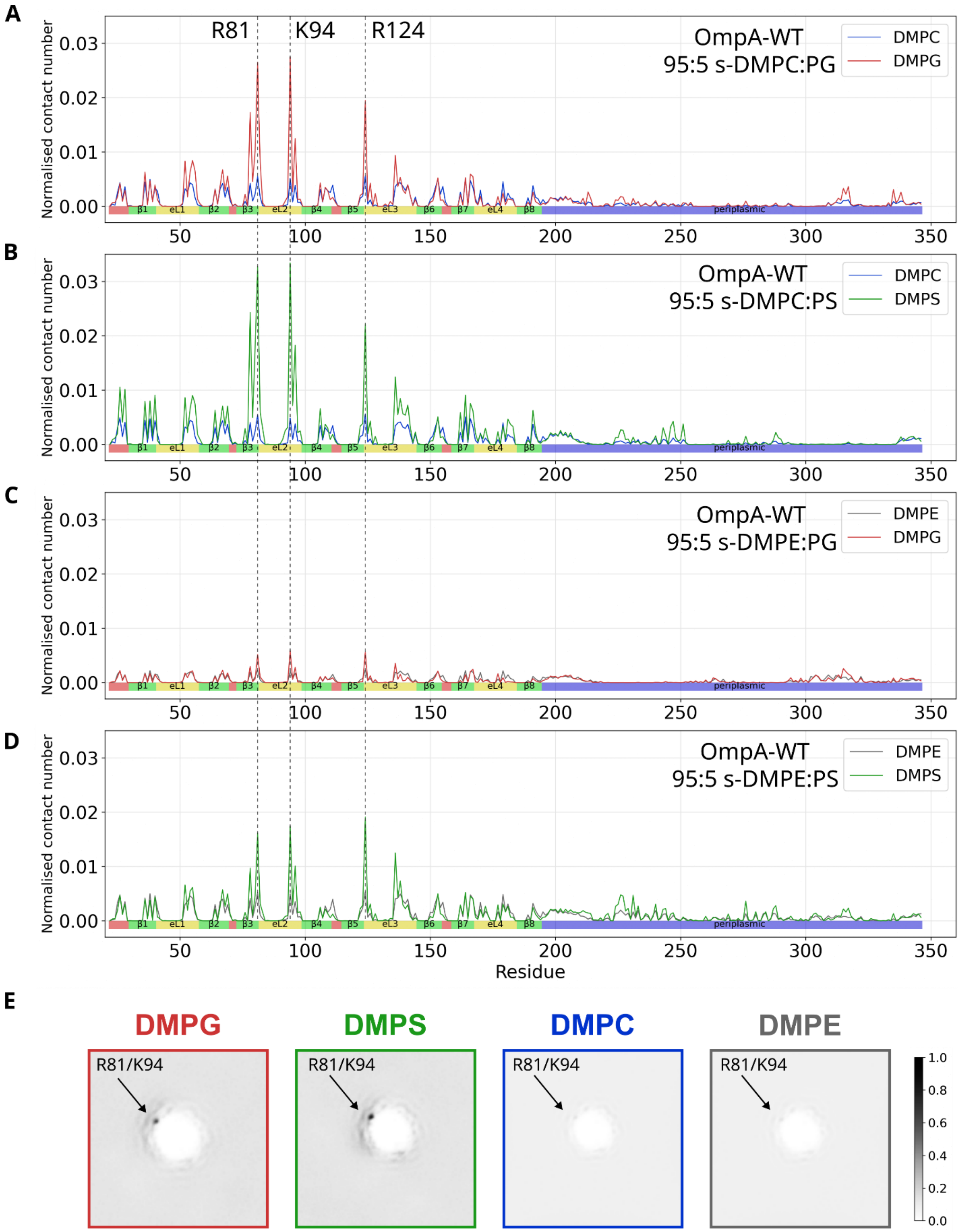

A
B
C
D
E
R81
K94
R124
OmpA-WT
95:5 s-DMPC:PG
95:5 s-DMPC:PS
95:5 s-DMPE:PG
95:5 s-DMPE:PS
DMPC
DMPG
DMPS
DMPE
Normalised contact number
Residue
periplasmic
R81/K94

Supplementary Figure 18: Normalised contact plots and lipid densities for full-length OmpA interaction with DMPC, DMPG, DMPS and DMPE symmetric lipid mixes. Normalised contact plots (number of interactions between each type of lipid and each protein residue normalised by lipid concentration and simulation frame number) for full length OmpA in **(A)** 95:5 s-DMPC:DMPG, **(B)** 95:5 s-DMPC:DMPS, **(C)** 95:5 s-DMPE:DMPG and **(D)** 95:5 s-DMPE:DMPS symmetric lipids (as indicated on panels). The lipid interacting residues R81, K94 and R124 are indicated by the dashed lines, and show consistent lipid-protein interactions with negatively charged lipids, although at different magnitudes. The sequence of OmpA is shown below (strands (green), extracellular loops (yellow) intracellular turns (red), and includes its C-terminal water soluble domain (blue)). **(E)** Average lipid density plots (scale bar on the right hand side, values normalised for lipid concentration) for each of the four lipids considered in the simulations (DMPE, DMPG, DMPS and DMPC), calculated for the lipid phosphates over the trajectory timeframe, following centring and fitting of the transmembrane region of OmpA. DMPC and DMPG calculated from 95:5 s-DMPC:DMPG, DMPS from 95:5 s-DMPC:DMPS and DMPE from 95:5 s-DMPE:DMPG systems. The data show a clear enrichment of lipids localised to OmpA for DMPG and DMPS, while no such enrichment occurs with DMPC or DMPE.

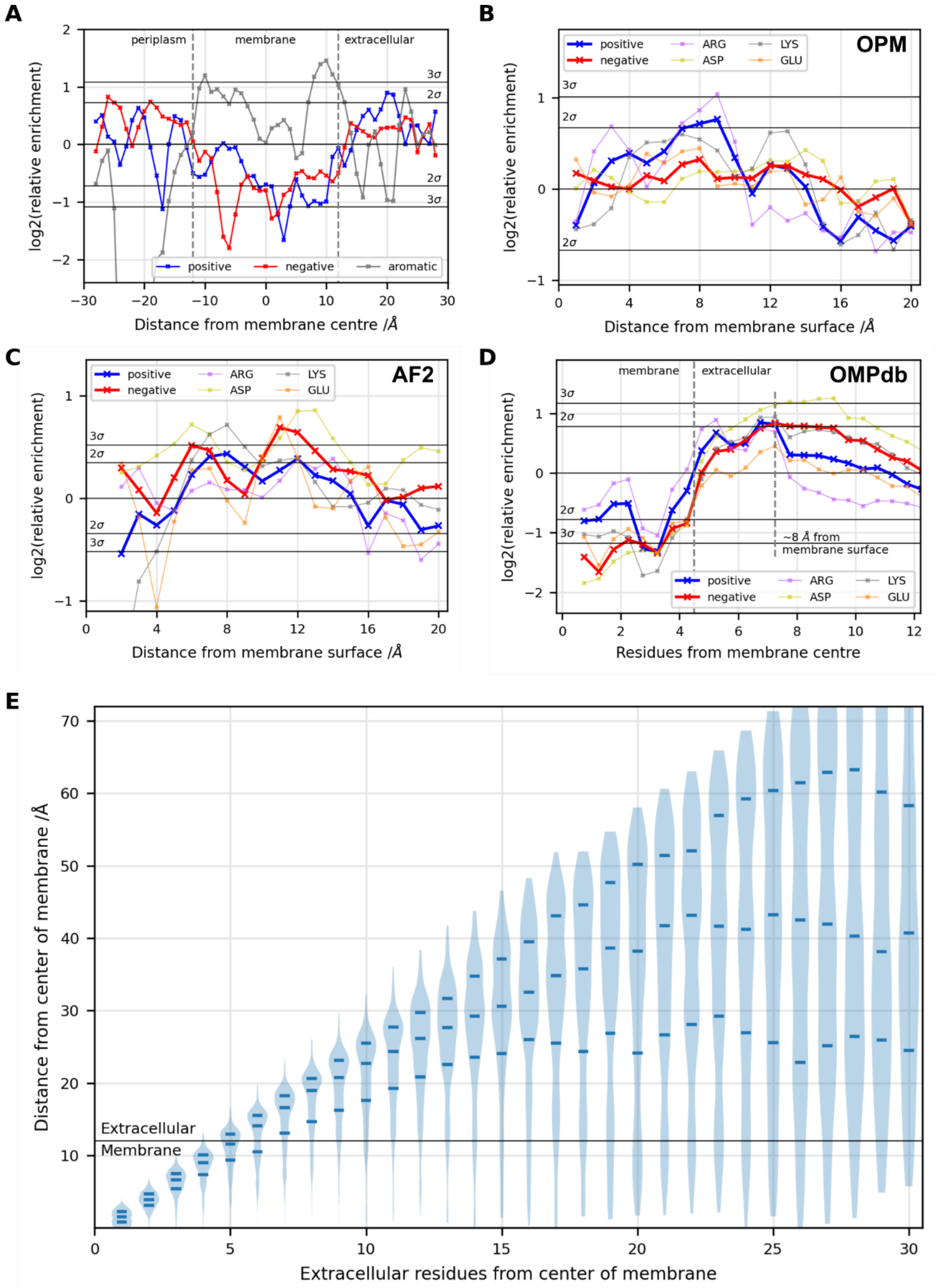
A
periplasm
membrane
extracellular
positive
negative
aromatic
Distance from membrane centre /Å
log2(relative enrichment)
B
OPM
positive
negative
ARG
ASP
LYS
GLU
Distance from membrane surface /Å
log2(relative enrichment)
C
AF2
positive
negative
ARG
ASP
LYS
GLU
Distance from membrane surface /Å
log2(relative enrichment)
D
OMPdb
membrane
extracellular
~8 Å from
membrane surface
positive
negative
ARG
ASP
LYS
GLU
Residues from membrane centre
log2(relative enrichment)
E
Extracellular
Membrane
Distance from center of membrane /Å
Extracellular residues from center of membrane

Supplementary Figure 19: All charged residue enrichments relative to membrane proximity. Average enrichment of positively and negatively charged residues, and the underlying enrichment of arginine/lysine and aspartic/glutamic acid relative to random chance for the **(A-B)** OPM, **(C)** Alphafold2 (AF2) and **(D)** OMPdb datasets. **(E)** Violin plots indicating the average distance from the centre of the membrane for the Cα of residues in extracellular loops in the OPM dataset (n = 75), showing the average and first and third quartile. Loops were truncated at their midpoints, and each part considered separately.

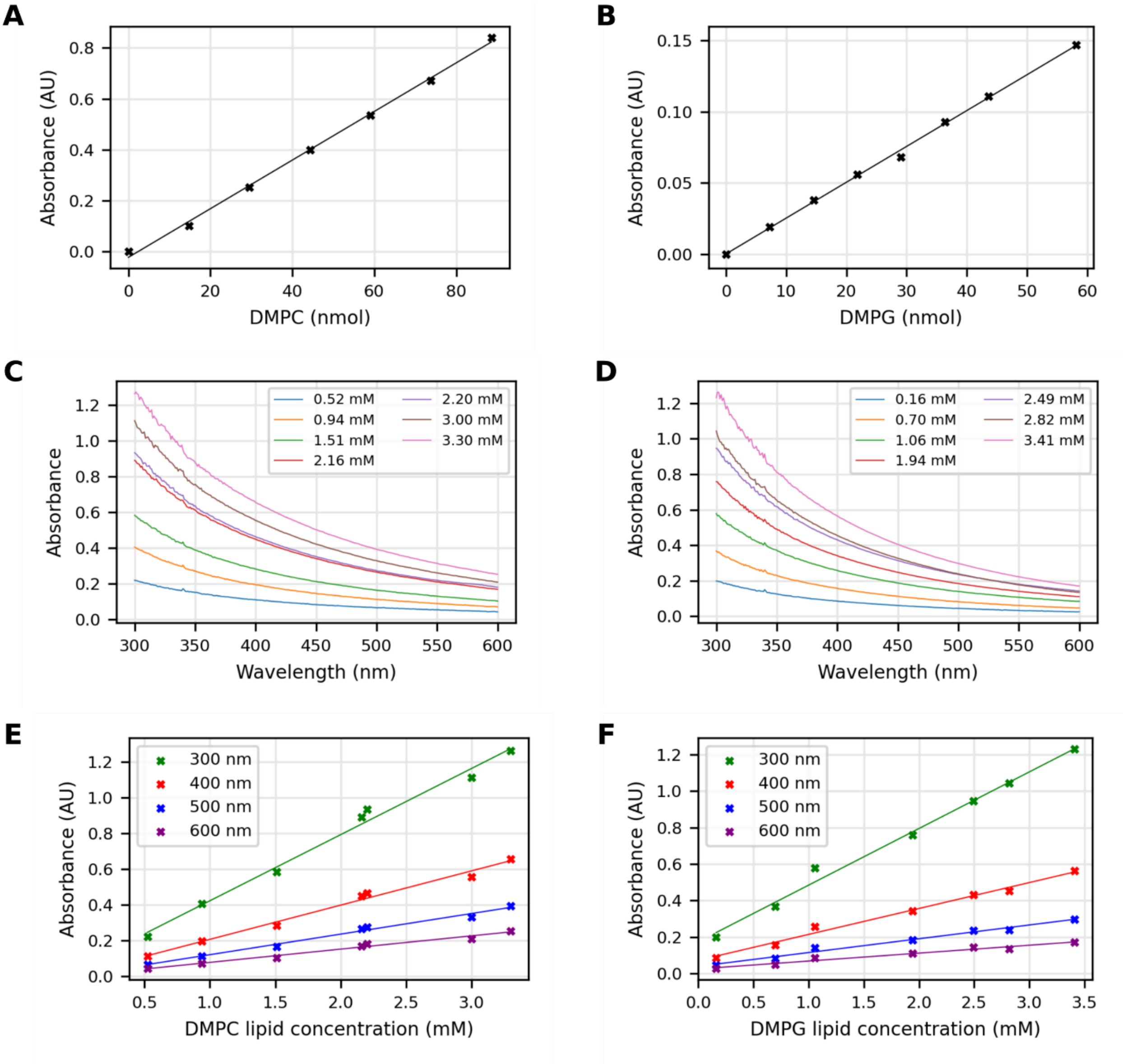

Supplementary Figure 20: Determining liposome concentration. (A,B) Liposome absorbance calibration lines to absolute lipid amount, determined using the Stewart assay for DMPC and DMPG. Absorbance curves for **(C)** DMPC and **(D)** DMPG liposomes at different measured lipid concentration (measured using the Stewart assay). **(E,F)** The absorbance values at 300 nm, 400 nm, 500 nm and 600 nm of DMPC and DMPG LUVs, respectively, with fitted straight lines, to generate absorbance-liposome concentration calibration curves.

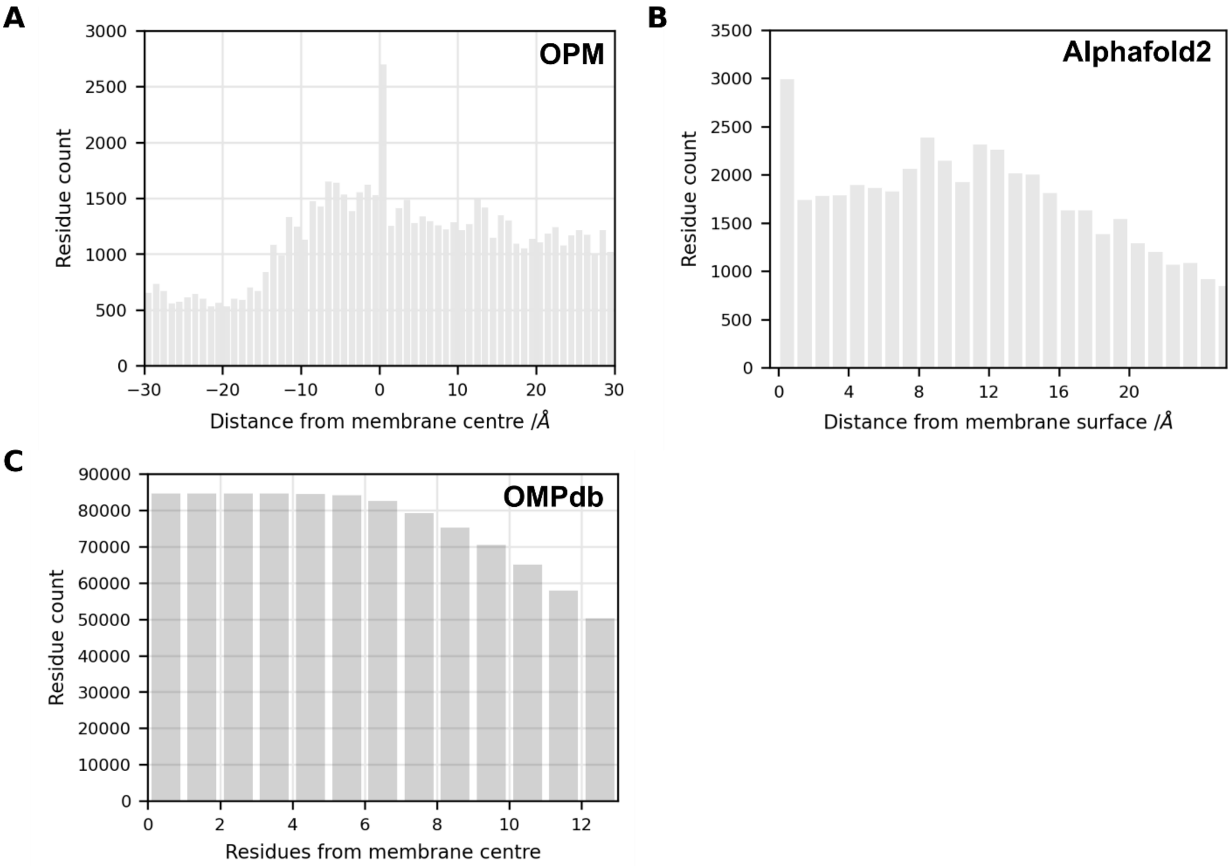

Supplementary Figure 21: Number of residues per z-axis slab for (A) OPM, (B) Alphafold2 and (C) OMPdb residue enrichment analysis. Residue count used to calculate enrichments in each z-axis slab (per Å for OPM and Alphafold2 datasets, per residue for OMPdb).

Supplementary Tables 1-13

Protein	Membrane Composition	Box size /nm	Time /µs	Replicas
OmpA WT	10:90 DMPC:DMPG	16x16x20	3	5
	25:75 DMPC:DMPG			
	50:50 DMPC:DMPG			
	30:70 DMPC:DMPG			
	85:15 DMPC:DMPG			
	95:5 DMPC:DMPG			
	97.5:2.5 DMPC:DMPG			
	95:5 DMPC:DMPS			
	95:5 DMPE:DMPG			
	95:5 DMPE:DMPS			
	Inner leaflet: DMPC<br>Outer leaflet: 90:10 DMPC:DMPG			
	Inner leaflet: 90:10 DMPC:DMPG<br>Outer leaflet: DMPC			
	Inner leaflet: DMPG<br>Outer leaflet: 10:90 DMPC:DMPG			
	Inner leaflet: 10:90 DMPC:DMPG<br>Outer leaflet: DMPG			
OmpA-M3	95:5 DMPC:DMPG			
BamA	95:5 DMPC:DMPG	20x20x20	3	5

Supplementary Table 1: Summary of all simulations run in this study. Membranes are lipid symmetric unless specific compositions for inner and outer leaflets are given. Membrane composition fraction is by lipid number. OmpA-M3 is OmpA with R81S, K94S and R124S mutations.

	p-values		
Liposome	OmpA-NC vs OmpA-WT	OmpA-NC vs OmpA-NP	OmpA-NC vs OmpA-NN
DMPC	0.018	0.05	0.457
DMPG	0.018	0.029	0.029
~10% a-	0.014	0.014	0.029
~20% a-	0.029	0.050	0.200
s-DMPC:PG	0.029	0.029	0.600
s-DMPC:PG	0.012	0.029	0.029

Supplementary Table 2: Statistical difference testing p-values showing that OmpA-NC folds faster than OmpA-WT, OmpA-NP and OmpA-NN in the majority of lipid environments. Significance was determined by permutation testing.

	p-values	
Liposome	OmpA-NP vs OmpA-WT	OmpA-NP vs OmpA-NN
DMPC	0.018	0.029
DMPG	0.008	0.014
~10% a-DMPG/PC	0.014	0.029
~20% a-DMPC/PG	0.029	0.050
s-DMPC:PG 90:10	0.018	0.029
s-DMPC:PG 20:80	0.005	0.014

Supplementary Table 3: Statistical difference testing p-values showing that OmpA-NP folds slower than OmpA-WT and OmpA-NN in all lipid environments. Significance was determined by permutation testing.

	p-values
Liposome	OmpA-M3 vs OmpA-NP
DMPC	0.029
DMPG	0.086
~10% a-DMPG/PC	1.000
~20% a-DMPC/PG	0.300
s-DMPC:PG 90:10	0.143
s-DMPC:PG 20:80	0.029

Supplementary Table 4: Statistical difference testing p-values showing that OmpA-NP folds at a similar rate to OmpA-M3 in most lipid environments. DMPC and s-DMPC:PG 20:80 are statistically different as OmpA-M3 did not fold over the experiment time while OmpA-NP did, albeit very slowly. Significance was determined by permutation testing.

	p-values
Liposome	OmpA-WT vs OmpA-NP
DMPC	0.0083
DMPG	0.0107
~10% a-DMPG/PC	0.0331
~20% a-DMPC/PG	0.0006
s-DMPC:PG 90:10	-
s-DMPC:PG 20:80	0.0007

Supplementary Table 5: Statistical difference testing p-values showing that OmpA-NP is significantly destabilised compared to OmpA-WT in all lipid environments. OmpA-NP did not unfold sufficiently in s-DMPC:PG 90:10 to allow for rigorous comparison. Significance was determined by permutation testing.

	p-values
Liposome	OmpA-WT vs OmpA-NN
DMPC	0.0257
DMPG	0.0006
~10% a-DMPG/PC	0.0286
~20% a-DMPC/PG	0.0007
s-DMPC:PG 90:10	0.0075
s-DMPC:PG 20:80	4E-05

Supplementary Table 6: Statistical difference testing p-values showing that OmpA-NN is significantly stabilised compared to OmpA-WT in all lipid environments. Significance was determined by permutation testing.

	p-values		
Liposome	OmpA-NP vs OmpA-M3	OmpA-WT vs OmpA-M3	OmpA-WT vs OmpA-NP
DMPC	0.20920	0.0198	0.0083
DMPG	0.00760	0.0004	0.0107
~10% a-DMPG/PC	0.21790	0.0304	0.0331
~20% a-DMPC/PG	0.39920	0.0005	0.0006
s-DMPC:PG 90:10	-	-	-
s-DMPC:PG 20:80	0.00580	0.015	0.0007

Supplementary Table 7: Statistical difference testing p-values showing that OmpA-NP and OmpA-M3 are similarly destabilised in most lipid environments. While OmpA-M3 is more significantly more destabilised than OmpA-NP in DMPG and s-DMPC:PG 20:80 membranes, both are significantly destabilised compared to OmpA-WT. OmpA-M3 and OmpA-NP did not unfold sufficiently in s-DMPC:PG 90:10 to allow for rigorous comparison. Significance was determined by permutation testing.

Liposome	p-values: OmpA-NN vs OmpA-WT	
	Folding kinetics	Urea stability
DMPG	1	0.0006
~20% a-DMPC/PG	0.826	0.0007
s-DMPC:PG 20:80	0.005	4E-05

Supplementary Table 8: Statistical difference testing p-values showing that OmpA-NN and OmpA-WT tend to be fold similar in inner-leaflet DMPG rich membranes, but have different stabilities. Significance was determined by permutation testing.

Protein	Liposome 1	Liposome 2	p-values
OmpA-NN	~10% a-DMPG/PC	s-DMPC:PG 90:10	0.029
OmpA-NN	~20% a-DMPC/PG	s-DMPC:PG 20:80	0.029
OmpA-NP	~10% a-DMPG/PC	s-DMPC:PG 90:10	1.000
OmpA-NP	~20% a-DMPC/PG	s-DMPC:PG 20:80	0.029

Supplementary Table 9: Statistical difference testing p-values showing for Figure 5, showing the folding rate differences/similarities between symmetric and asymmetric liposomes for OmpA-NN and OmpA-NP. Significance was determined by permutation testing.

Protein	Liposome 1	Liposome 2	p-values
OmpA-NN	~10% a-DMPG/PC	s-DMPC:PG 90:10	0.42361
OmpA-NN	~20% a-DMPC/PG	s-DMPC:PG 20:80	0.01959
OmpA-NP	~10% a-DMPG/PC	s-DMPC:PG 90:10	0.03125
OmpA-NP	~20% a-DMPC/PG	s-DMPC:PG 20:80	0.01648

Supplementary Table 10: Statistical difference testing p-values showing for Figure 5, showing the urea stability differences/similarities between symmetric and asymmetric liposomes for OmpA-NN and OmpA-NP. Significance was determined by permutation testing.

Supplementary Table 11: Statistical P-values for all pairwise difference comparisons of kinetic data. P-values were determined using permutation testing and the number of permutations above the threshold is indicated (Methods), Data are organised by the figure the data was presented in. Data are provided as an Excel file.

Supplementary Table 12: Statistical P-values for all pairwise difference comparisons of urea stability data. P-values were determined using paired two-tailed t-test for all (OmpA mutant, lipid environment) pairs. Data are provided as an Excel file

Supplementary Table 13: Datasets used for bioinformatics analysis. PDB accession codes for all OPM structures and uniport accession codes for all Alphafold2 structures and OMP sequences considered in the bioinformatic analysis. Datasets are shown before and after sequence clustering (70% identity). Data are provided as an Excel file.

Unprocessed TLCs and gels for supplementary figures

Supplementary figure 3

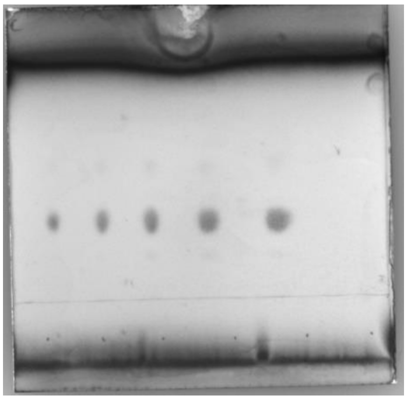

DMPC

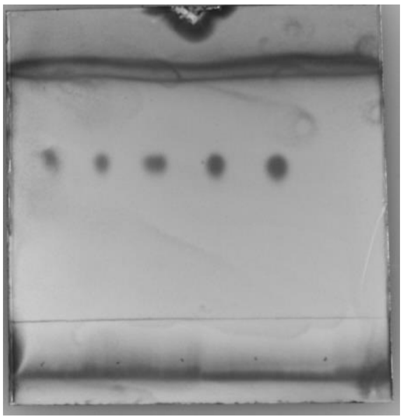

DMPE

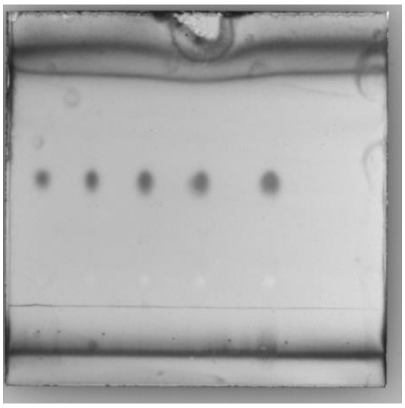

DMPG

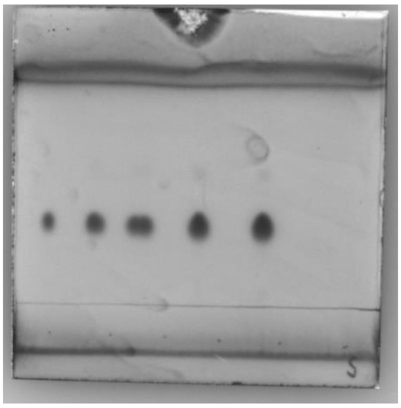

DMPS

Supplementary figure 7

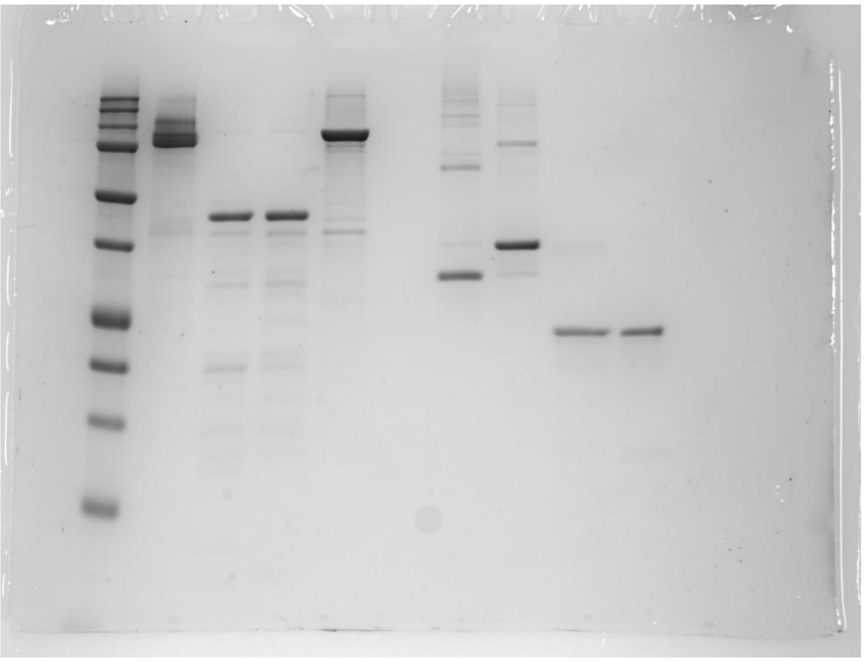

ladder (kDa: 250, 150, 100, 75, 50, 37, 25, 20, 15, 10, 5, 2)

Lanes	protein	boil	trypsin	DDM
1	*ladder*			
2	bama	-	-	-
3	bama	+	+	-
4	bama	+	+	+
5	bama	+	-	+
6	(*empty*)			
7	ompa	-	-	-
8	ompa	+	-	+
9	ompa	+	+	-
10	ompa	+	+	+

Supplementary figure 8

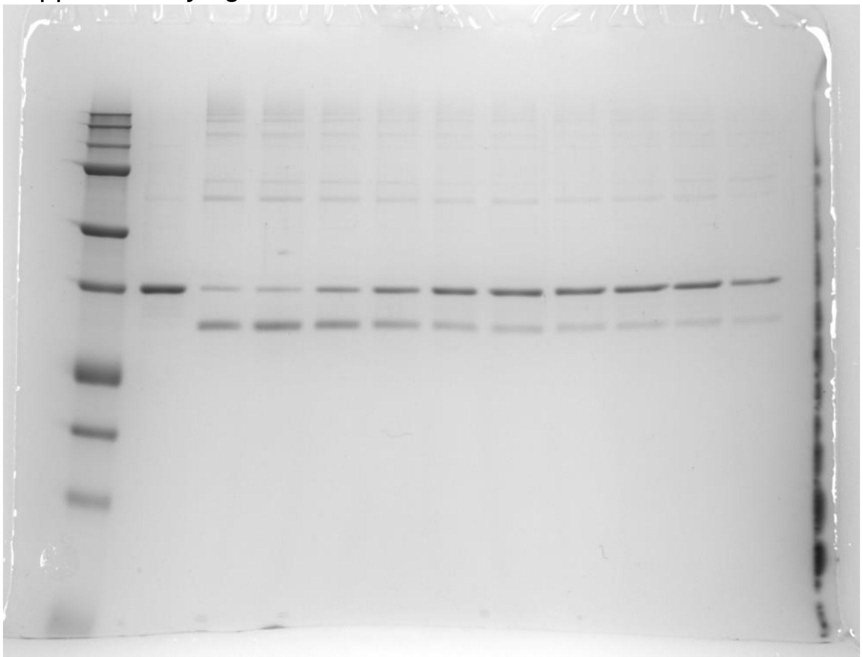

Protein: OmpA-WT (1.5 uM, in 100 mM NaCl and 10 mM Tris-Cl pH 8.5)

Liposome: DMPC (symmetric)

Lanes:

1 ladder (kDa: 250, 150, 100, 75, 50, 37, 25, 20, 15, 10, 5, 2)

2 boiled OmpA

3 0.5 M Urea

4 1.0 M Urea

5 1.5 M Urea

6 2.0 M Urea

7 2.5 M Urea

8 3.0 M Urea

9 3.5 M Urea

10 4.0 M Urea

11 4.5 M Urea

12 5.0 M Urea

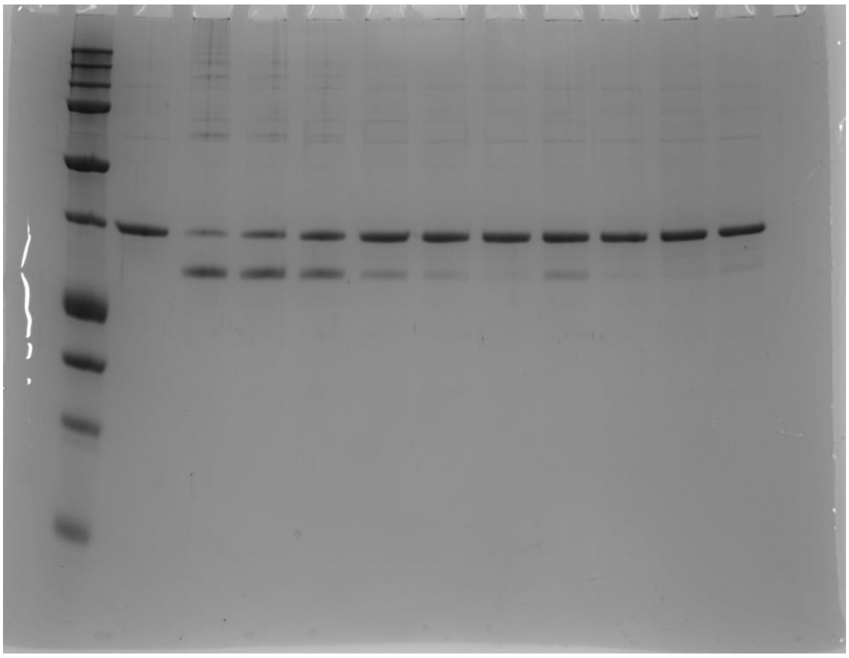

Protein: OmpA-WT (1.5 uM, in 100 mM NaCl and 10 mM Tris-Cl pH 8.5)

Liposome: ~10% DMPG/DMPC (asymmetric)

Lanes:

1 ladder (kDa: 250, 150, 100, 75, 50, 37, 25, 20, 15, 10, 5, 2)

2 boiled OmpA

3 0.5 M Urea

4 1.0 M Urea

5 1.5 M Urea

6 2.0 M Urea

7 2.5 M Urea

8 3.0 M Urea

9 3.5 M Urea

10 4.0 M Urea

11 4.5 M Urea

12 5.0 M Urea

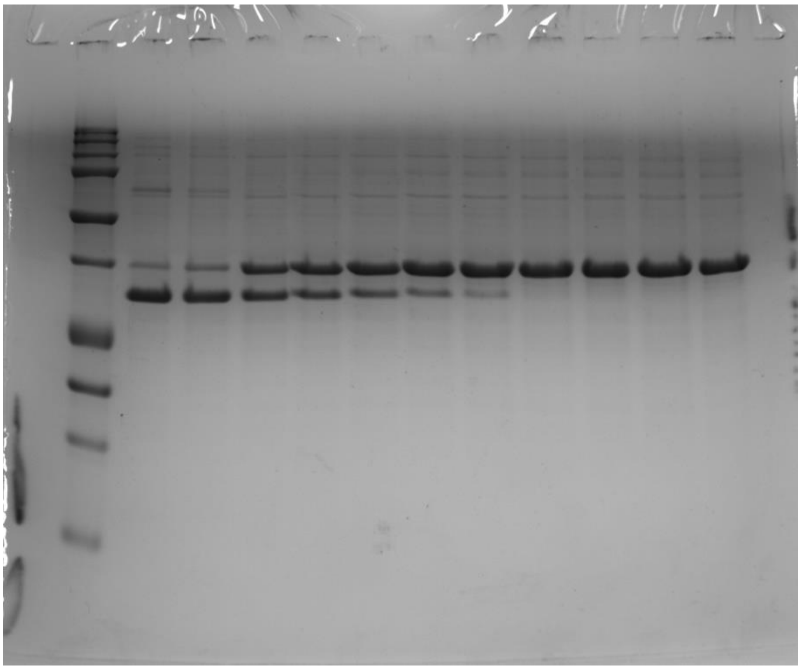

Protein: OmpA-WT (1.5 uM, in 100 mM NaCl and 10 mM Tris-Cl pH 8.5)

Liposome: DMPG (symmetric)

Lanes:

1 ladder (kDa: 250, 150, 100, 75, 50, 37, 25, 20, 15, 10, 5, 2)

2 3.0 M Urea

3 3.5 M Urea

4 4.0 M Urea

5 4.5 M Urea

6 5.0 M Urea

7 5.5 M Urea

8 6.0 M Urea

9 6.5 M Urea

10 7.0 M Urea

11 7.5 M Urea

12 8.0 M Urea

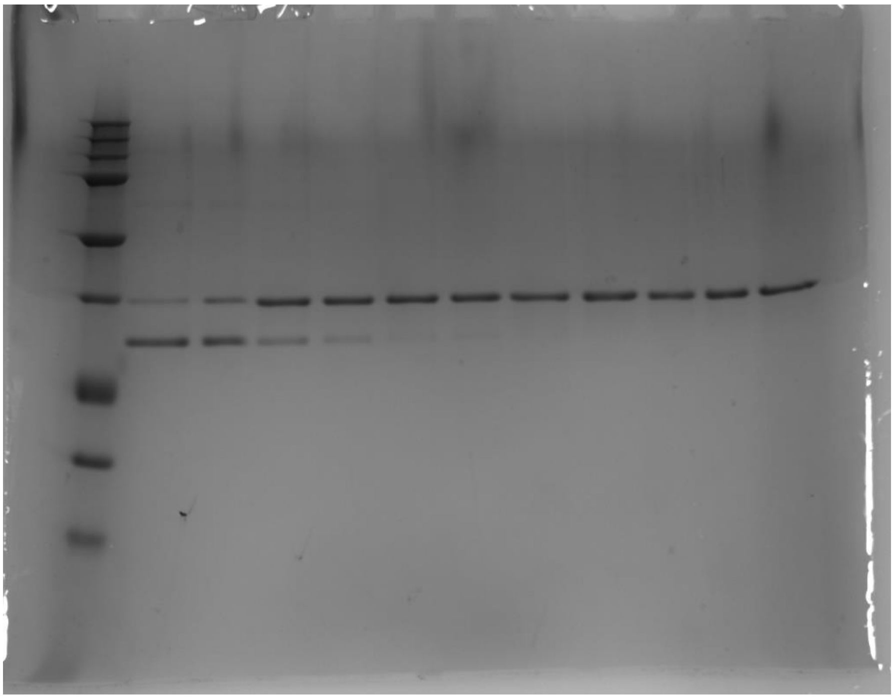

Protein: OmpA-WT (1.5 uM, in 100 mM NaCl and 10 mM Tris-Cl pH 8.5)

Liposome: ~20% DMPC/DMPG (asymmetric)

Lanes:

1 ladder (kDa: 250, 150, 100, 75, 50, 37, 25, 20, 15, 10, 5, 2)

2 3.0 M Urea

3 3.5 M Urea

4 4.0 M Urea

5 4.5 M Urea

6 5.0 M Urea

7 5.5 M Urea

8 6.0 M Urea

9 6.5 M Urea

10 7.0 M Urea

11 7.5 M Urea

12 8.0 M Urea

Supplementary figure 11

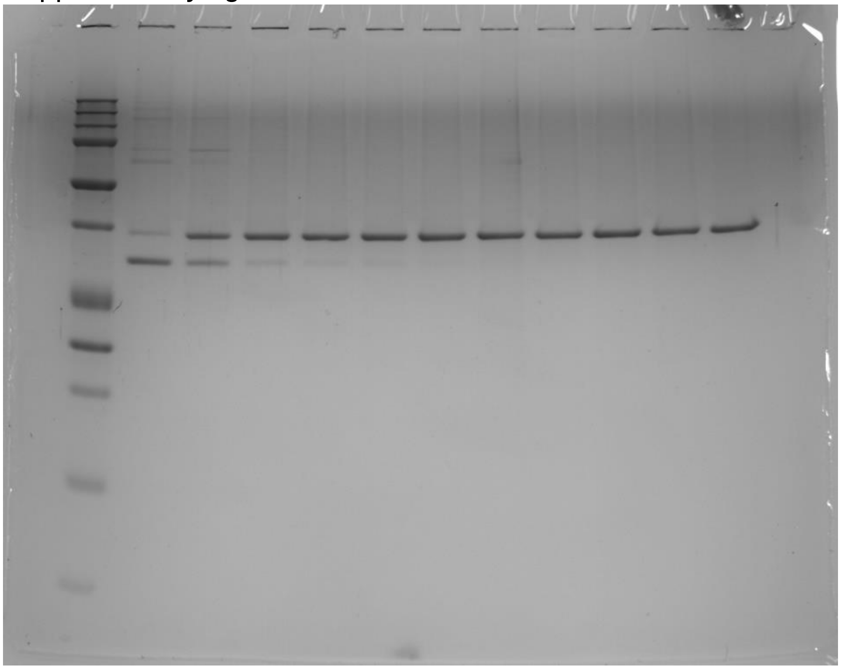

Protein: OmpA-WT (1.5 uM, in 100 mM NaCl and 10 mM Tris-Cl pH 8.5)

Liposome: DMPC:DMPS 90:10 (symmetric)

Lanes:

1 ladder (kDa: 250, 150, 100, 75, 50, 37, 25, 20, 15, 10, 5, 2)

2 0.5 M Urea

3 1.0 M Urea

4 1.5 M Urea

5 2.0 M Urea

6 2.5 M Urea

7 3.0 M Urea

8 3.5 M Urea

9 4.0 M Urea

10 4.5 M Urea

11 5.0 M Urea

12 5.5 M Urea

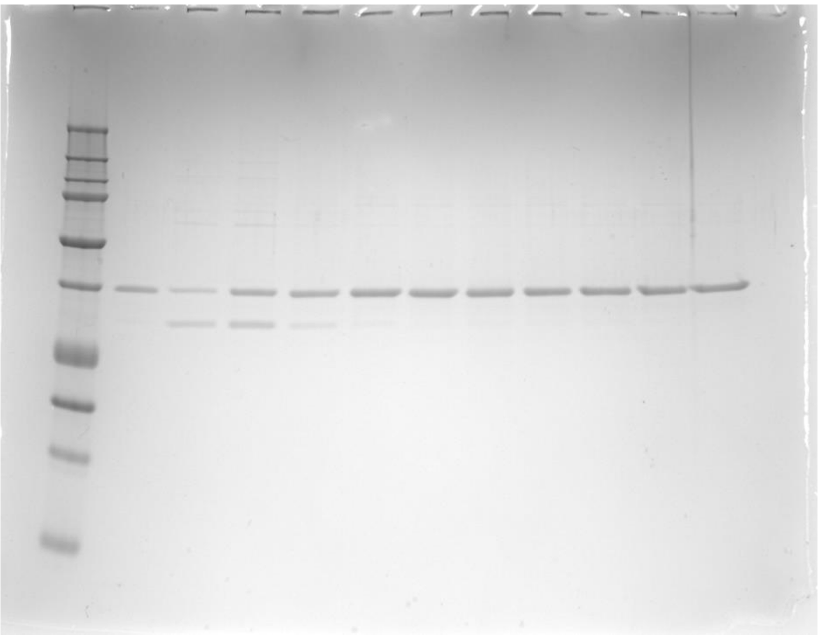

Protein: OmpA-WT (1.5 uM, in 100 mM NaCl and 10 mM Tris-Cl pH 8.5)

Liposome: ~20% DMPS/DMPC (asymmetric)

Lanes:

1 ladder (kDa: 250, 150, 100, 75, 50, 37, 25, 20, 15, 10, 5, 2)

2 boiled OmpA

3 0.5 M Urea

4 1.0 M Urea

5 1.5 M Urea

6 2.0 M Urea

7 2.5 M Urea

8 3.0 M Urea

9 3.5 M Urea

10 4.0 M Urea

11 4.5 M Urea

12 5.0 M Urea

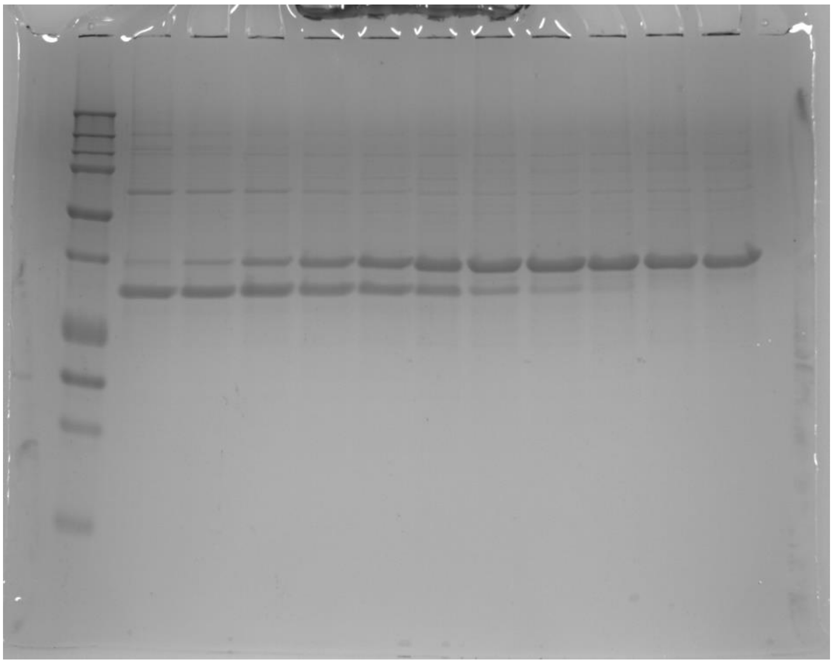

Protein: OmpA-WT (1.5 uM, in 100 mM NaCl and 10 mM Tris-Cl pH 8.5)

Liposome: DMPE:DMPG 20:80 (symmetric)

Lanes:

1 ladder (kDa: 250, 150, 100, 75, 50, 37, 25, 20, 15, 10, 5, 2)

2 1.5 M Urea

3 2.0 M Urea

4 2.5 M Urea

5 3.0 M Urea

6 3.5 M Urea

7 4.0 M Urea

8 4.5 M Urea

9 5.0 M Urea

10 5.5 M Urea

11 6.0 M Urea

12 6.5 M Urea

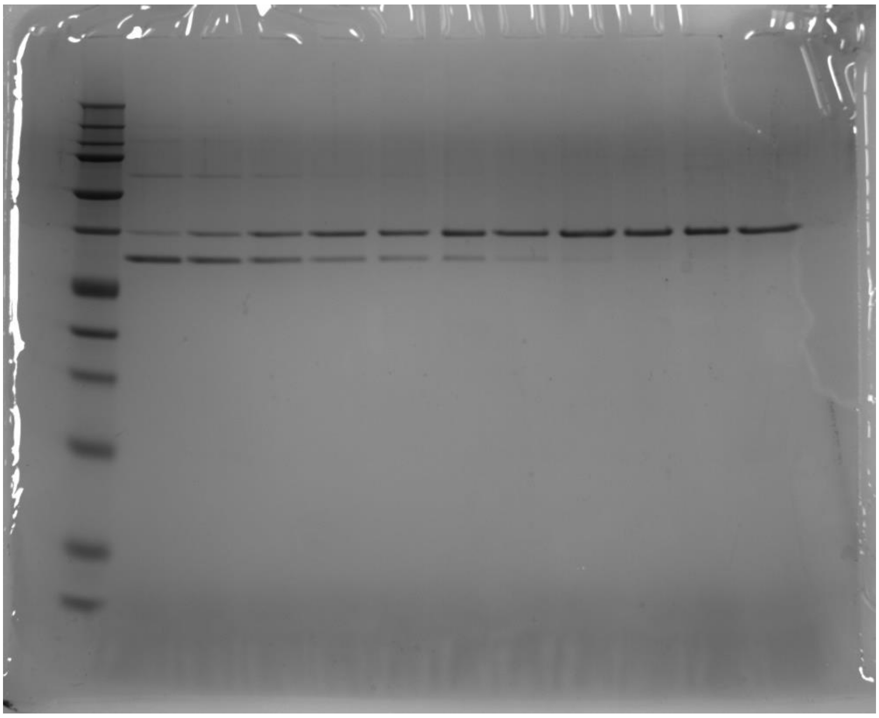

Protein: OmpA-WT (1.5 uM, in 100 mM NaCl and 10 mM Tris-Cl pH 8.5)

Liposome: ~20% DMPE/DMPG 20:80 (asymmetric)

Lanes:

1 ladder (kDa: 250, 150, 100, 75, 50, 37, 25, 20, 15, 10, 5, 2)

2 3.0 M Urea

3 3.5 M Urea

4 4.0 M Urea

5 4.5 M Urea

6 5.0 M Urea

7 5.5 M Urea

8 6.0 M Urea

9 6.5 M Urea

10 7.0 M Urea

11 7.5 M Urea

12 8.0 M Urea
