# Supplementary figures and images for: Protein–lipid charge interactions control the folding of outer membrane proteins into asymmetric membranes
Source: Nat Chem. 2023 Sep 14;15(12):1754–64. doi: 10.1038/s41557-023-01319-6 (PMC10695831; doi:10.1038/s41557-023-01319-6)

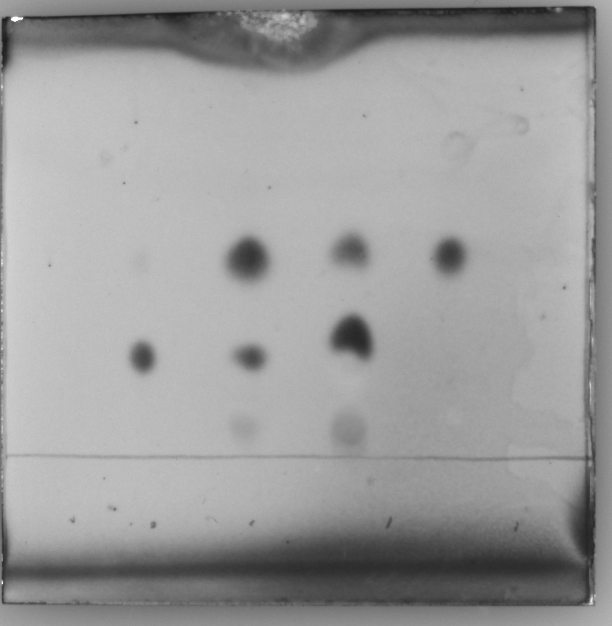

Supplement: Supplementary file 6 — Unprocessed TLC for Fig. 1e. [file 41557_2023_1319_MOESM6_ESM.tif]

(b)

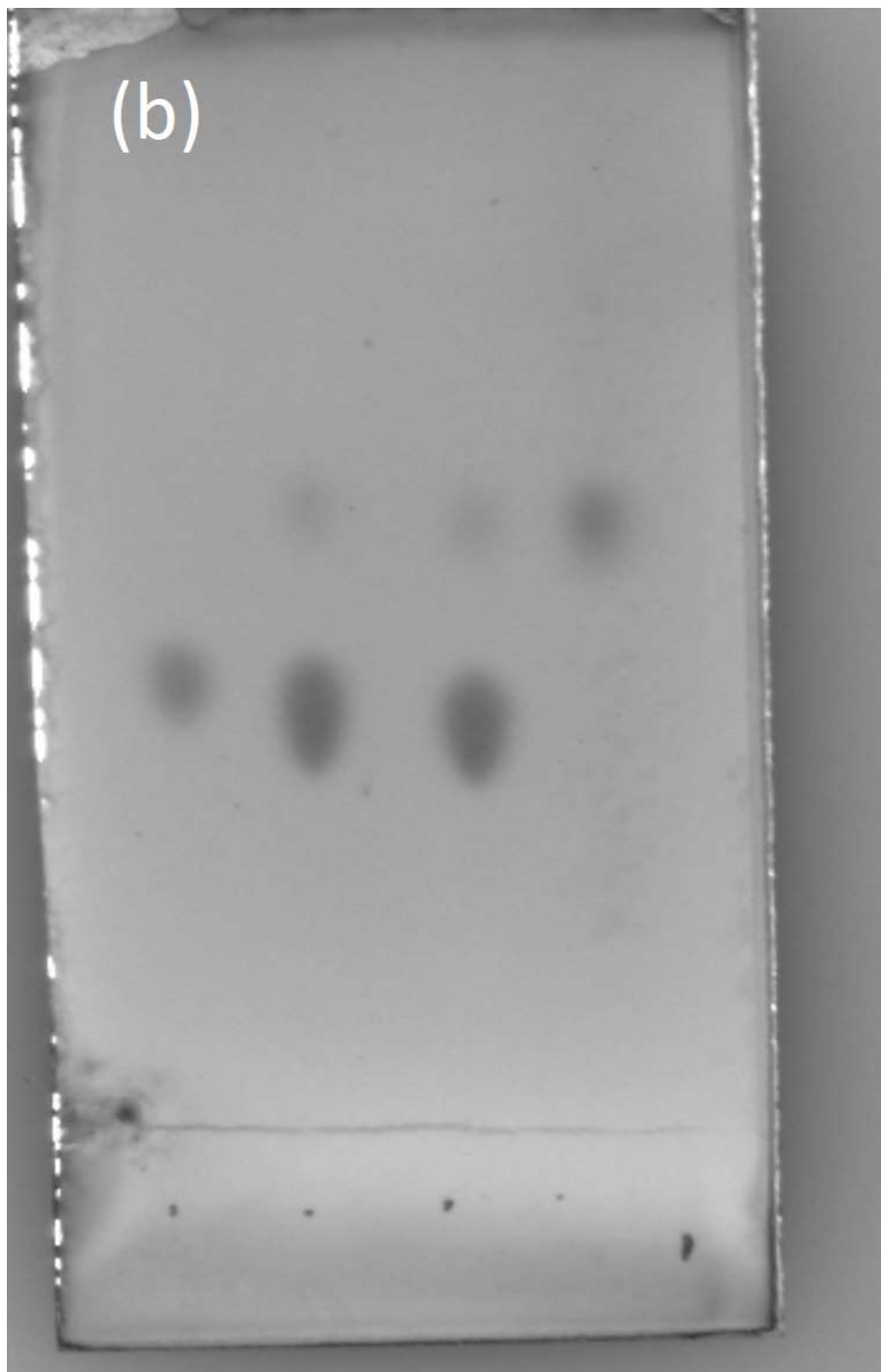

(f)

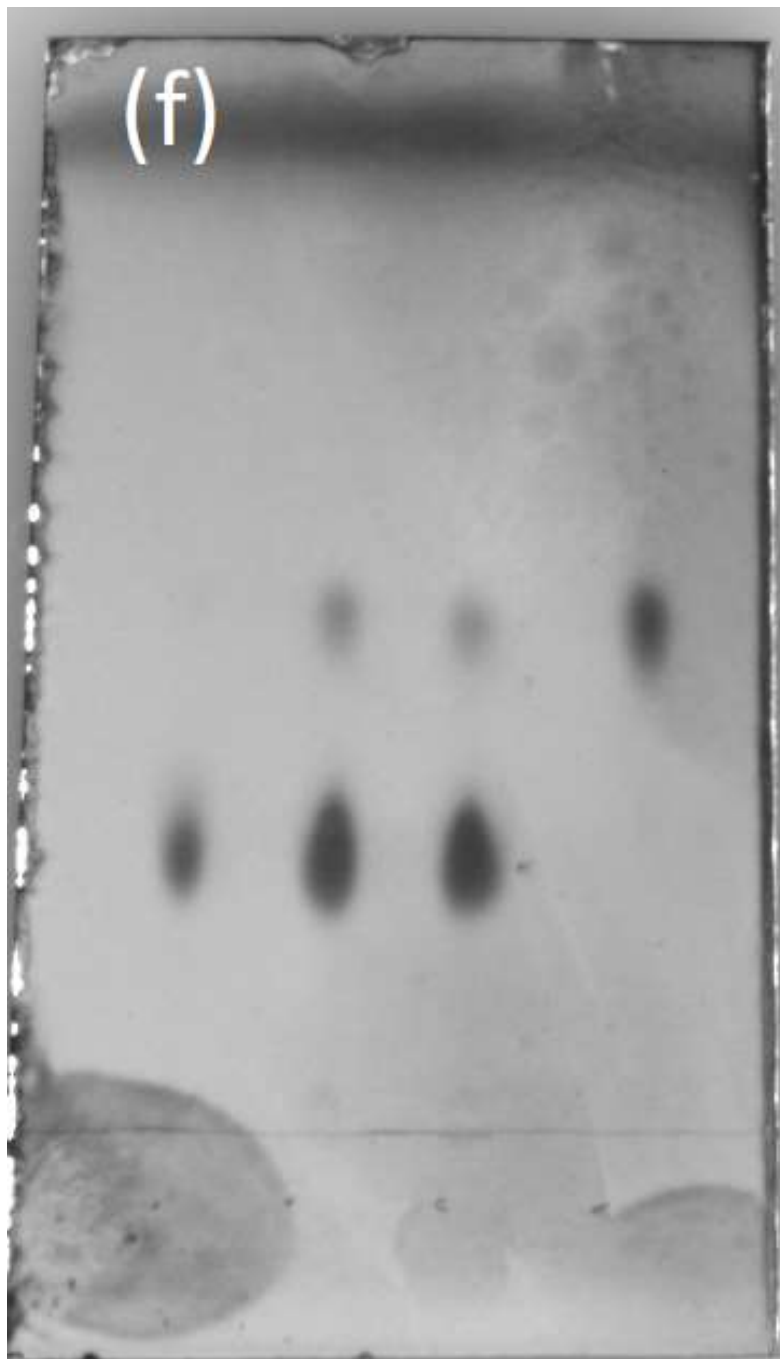

Supplement: Supplementary file 17 — Unprocessed TLC for Extended Data Fig. 5b,f. [file 41557_2023_1319_MOESM17_ESM.pdf]

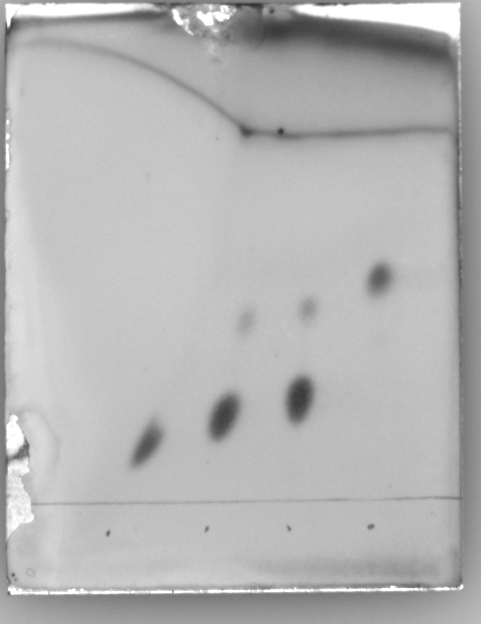

Supplement: Supplementary file 19 — Unprocessed TLC for Extended Data Fig. 6e. [file 41557_2023_1319_MOESM19_ESM.tif]
